# Optimizing urban bus network based on spatial matching patterns for sustainable transportation: A case study in Harbin, China

**DOI:** 10.1371/journal.pone.0312803

**Published:** 2024-10-28

**Authors:** Boya Gao, Jie Liu

**Affiliations:** School of Civil Engineering and Transportation, Northeast Forestry University, Harbin, China; Southwest Jiaotong University, CHINA

## Abstract

The rapid economic development and accelerating urbanization have led to a significant mismatch between the urban bus network allocation and the population flow. Therefore, this paper investigates this challenge by exploring the intricate relationship between the population flow dynamics, traffic congestion conditions, and the efficient allocation of bus resources. In response, two key indexes were introduced based on spatial matching patterns to assess the urban bus network: the Population-Bus Match Index evaluates the matching degree between supply and demand, and the Population-Congestion Match Index evaluates the matching degree between utilization and saturation. Additionally, two distinct optimization strategies have been proposed to enhance the urban bus network. The first optimization strategy considers the bus network’s current status, while the second aspires to an idealized scenario. Subsequently, the potential contributions of each bus station in reducing CO_2_ emission reduction after implementing the two optimization strategies are quantified. Utilizing a case study focused on Harbin, the proposed methods are validated. The findings unveil a substantial misalignment between supply and demand within the bus network during peak periods, with nearly half of the bus stations experiencing a disparity between utilization and saturation. Comparative experiments across different optimization strategies reveal that the second optimization strategy significantly outperforms the first, but the first optimization strategy has a higher degree of CO_2_ emission reduction contribution. The results of this study provide decision-makers with an environmentally oriented vantage point for the discerning selection of optimization strategies and leave valuable insights for urban areas confronting transportation challenges.

## 1 Introduction

Urbanization is an ongoing global trend, with many people living in urban areas [1]. According to the United Nations (UN) report [2], the global urban population doubled from 25% in 1950 to about 50% in 2020. By 2050, the global population will reach 9.7 billion, and 68%, i.e., 6.6 billion people, will live in urban areas. This rapid urbanization introduces multiple challenges, with transportation emerging as one of the most pressing issues, including traffic congestion, increased carbon dioxide (CO_2_) emissions, and the need for sustainable transportation solutions [3]. Within this dynamic urban landscape, public transportation systems, particularly bus networks, have become essential components of urban mobility. They serve as the lifeblood of cities, connecting residents to workplaces, schools, and essential services while providing a cost-effective and environmentally responsible means of transportation [4]. However, like any complex system, urban bus networks face several inefficiencies and imperfections. These issues manifest in various ways, including poorly planned bus routes that result in longer travel times and reduced convenience for passengers [5]. Inconveniently located bus stations may force passengers to walk long distances or face safety concerns, diminishing the system’s accessibility and usability [6]. Additionally, the uneven distribution of bus resources often leads to an overallocation of buses in some areas and underallocation in others, further exacerbating the system’s inefficiency [7].

In light of these challenges, optimizing urban bus networks has become an urgent priority. Scholars have increasingly focused on improving the efficiency of bus networks to alleviate traffic congestion and reduce CO_2_ emissions. This research aims to contribute to these efforts by exploring innovative strategies for optimizing urban bus networks based on spatial matching patterns. By aligning the supply of bus supply with the spatial distribution of demand, it is possible to analyze the current bus network configurations, identify gaps and inefficiencies, and propose data-driven solutions tailored to the city’s unique characteristics. Furthermore, the study will evaluate the potential environmental and social benefits of the optimized network, contributing to broader goals of sustainable urban transportation.

## 2 Literature review

Bus network optimization is a research area that has received significant attention, with the literature offering several studies [8]. The literature on this topic encompasses numerous studies focusing on the optimization of bus routes, bus stations, and the allocation of bus network resources. From the perspective of bus route optimization, an increasing number of researchers investigate strategies for bus route optimization by building multi-objective bus route optimization models and using heuristic algorithms to find the optimal solution. Multi-objective bus route optimization has various optimization objectives, such as minimum bus mileage run, minimum travel time, minimum operation cost, and maximum population flow. Sang et al. [9] introduced uncertainty theory into the custom bus route optimization problem and established an uncertainty custom bus route optimization model with the objective of minimizing the total bus mileage run, which ultimately resulted in a 9.55% reduction in total miles run. Ma et al. [10] constructed robust optimization models with the objective of minimizing passenger travel time and minimizing carbon emissions from custom buses. The final solution included three customized bus parking lots and 20 drop-off and pick-up stations. Calabrò et al. [11] searched for the lowest-cost paths in the road network, maximizing service coverage based on potential traffic demand and required travel time, in order to improve the efficiency of the entire transit system. With the optimization objectives of maximizing the direct population flow and minimizing the route duplication factor, Wu et al. [12] adjusted the bus routes of the existing bus network to minimize the deviation of the adjusted network from the existing network while improving the quality of bus service. Typical heuristic algorithms include ant colony, particle swarm, genetic, and adaptive large neighborhood search algorithms [13]. For instance, Wei et al. [14] proposed a bus route network optimization method based on Ant Colony Optimization(ACO) algorithm, which takes into account the existing bus route network with the objective of maximizing direct passenger flow, and does not alter the existing network, but only adds new bus routes to the existing network according to the needs of urban development. Lin et al. [15] proposed an improved Multi-Objective Adaptive Particle Swarm Optimization (MOAPSO) algorithm, which considers the interests of all parties from the perspective of bus companies, passengers, and governmental departments, and ultimately achieves the optimization objectives of minimizing the fixed cost, fuel cost, carbon emission cost, and time-window penalty cost of bus operation cost. Tang et al. [16] designed an improved Non-dominated Sorting Genetic Algorithm-II (NSGA-II) to optimize bus schedules by considering the minimization of the total waiting time of passengers and the departure time of the bus company. Szeto and Wu [17] proposed an algorithm for designing bus routes in Hong Kong that minimizes the total travel time and intermediate points for residents by combining a genetic algorithm with a neighborhood search heuristic to improve the existing bus routes by reducing the number of transfers and the total travel time for passengers.

Regarding bus station optimization, Chien and Yang [18] employed a genetic algorithm to establish the objective function of minimizing user and supplier costs to find the optimal number and location of bus stations serving CBD(Central Business District) and residential districts. Chien and Qin [19] used supplier and user costs as the objective function to optimize the number and location of bus stations to improve the accessibility of bus services. Moura et al. [20] proposed a two-stage urban bus station location model that investigates the location variation of bus stations using different traffic flows, public exchanges, and signaling sequences. Supangat and Soelistio [21] used mean shift algorithm and ant colony algorithm to optimize the location of bus stops in West Jakarta and successfully determined the optimal solution of bus stations and their suboptimal routes. Shatnawi et al. [22] modeled the location of bus stations in the city of Amman, Jordan, in order to find the optimal travel time and the service capacity of the bus stations. Ceder et al. [23] mathematically modeled the bus station layout based on topography using a heuristic evolutionary algorithm. The study found the best layout for bus stations, which improved transit quality and increased ridership.

When optimizing bus stations and routes, some scholars have also paid attention to the degree of spatial resource allocation of the urban bus network, and selected indicators from the perspective of the balance of supply and demand for research [24–26]. With regard to the selection of supply indicators, Minocha et al. [27] proposed a system of indicators for evaluating the supply of buses, including the frequency of service, service hours, service coverage and other indicators. Li et al. [28] used bus stops and their associated total daily bus arrivals to measure the level of bus supply. Currie [29] used the frequency of service and spatial coverage of bus stops to measure the level of bus supply and identified a match between the distribution of public transport facilities and demand from a supply and demand perspective based on the economic and social attributes of the population. On the choice of demand indicators, Liao et al. [30] have used population density to measure the public transport needs of residents. Yao et al. [31] used three main indicators, namely, the nature of the land, socio-economic and structural characteristics of the public transport network, to measure the demand for buses. Bi et al. [32] used data from smart cards and Quick Response (QR) codes for bus demand. Based on supply and demand indicators, some scholars have used Gini coefficients and the Lorenz curve to assess the allocation of public transport resources, such as Gori et al. [33] combine the Gini coefficients and the Lorenz curve to measure resource allocation from the perspective of the "quality" of the public transport system. Delbosc et al. [34] used Lorenz curve to compare the distribution of transit resource provision in population and employment in Melbourne, Australia.

This paper contributes to the existing literatures from several perspectives. Firstly, although some scholars have examined the spatial matching of population and bus resources from the perspective of supply and demand balance, such research is relatively sparse. Most of these studies focus on exploring the influence mechanisms or proposing qualitative optimization strategies based on global and local matching situations. Quantitative optimization studies based on the spatial local matching of urban bus networks are limited. This study proposes two quantitative optimization strategies based on the spatial local matching of bus supply and demand. The first optimization strategy involves adjusting the resource allocation within the constraints of the current bus network capacity. This method is practically feasible in the short term and can quickly address the issue of supply-demand mismatch, especially in areas with high demand but insufficient supply. The second optimization strategy achieves the desired matching of supply and demand by adjusting bus capacity. This strategy provides a long-term perspective, aiming to comprehensively improve the efficiency and service quality of the bus network, although its implementation requires more significant modifications. Although economic development levels, transportation infrastructure, and weather conditions significantly influence residents’ willingness to use public transportation [35, 36], the core focus of this study is the spatial matching in bus network optimization—specifically, the relationship between population flow and bus capacity. This spatial matching is crucial for enhancing the overall efficiency of the bus system [37], especially when optimizing under existing infrastructure conditions and current levels of economic development [38]. Consequently, this study reasonably omits the influence of economic development levels and transportation infrastructure on travel preferences, opting instead to use data from non-rainy, non-snowy days to control for external variables and ensure that the results are centered on the core issue of bus network optimization. Secondly, in terms of index selection, this study not only considers actual population flow dynamics (demand and utilization) and bus resource allocation (supply) but also introduces a new index: traffic congestion, to measure the saturation level of the roads. By quantifying the spatial matching degree between the utilization and saturation of each bus station, the study can identify bus stations with severe issues for optimization. Traditional bus network optimization research typically focuses on supply-demand matching and resource allocation, often overlooking the impact of traffic congestion on the efficiency of the bus system. This study expands the perspective of bus network optimization by incorporating traffic congestion as an evaluation dimension. The introduction of traffic congestion allows for a more accurate reflection of the actual traffic conditions in the areas where bus stations are located, providing more comprehensive data support for formulating optimization strategies [9]. Lastly, an important contribution of this study is the quantification of the potential CO_2_ emission reductions resulting from the proposed optimization strategies. By estimating the environmental benefits of improved bus resource allocation, this research highlights the broad environmental impacts of urban transportation planning and aligns with global sustainability goals.

The remainder of the paper is organized as follows. Section 3 describes the study area and the dataset used. Section 4 presents two established spatial matching indexes, the two optimization strategies, and the methodology for assessing the contribution of CO_2_ emission reduction. Section 5 reports and analyzes the results. Section 6 discusses the findings in comparison with existing literature. Section 7 summarizes the study and suggests future research directions.

## 3 Study area and data

### 3.1 Study area

**Fig 1:**
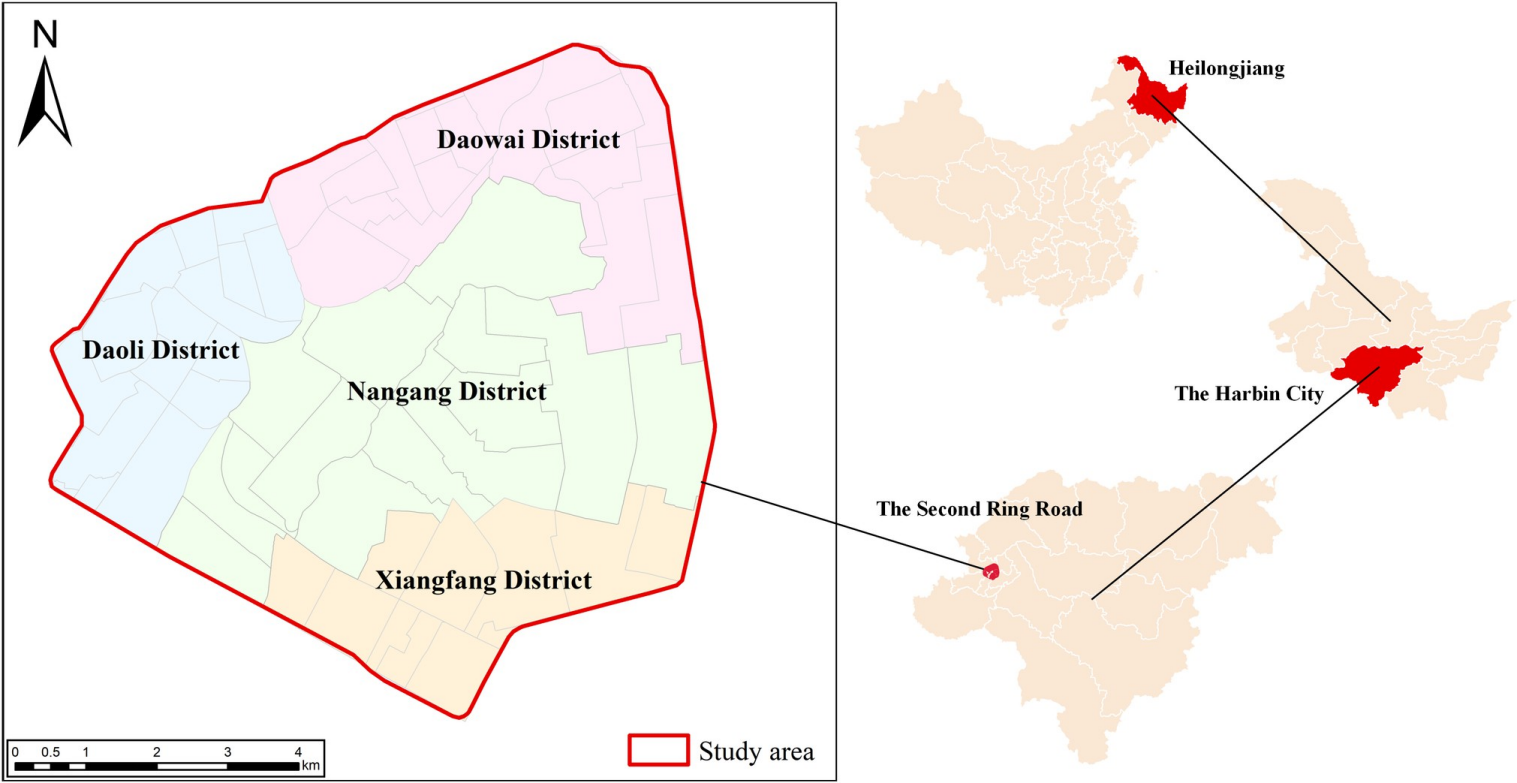
Study area and division of administrative districts within the Second Ring Road in Harbin. Note: self-drawn image. Source: latitude and longitude from OpenStreetMap.

This paper focuses on Harbin (125°42′-130°10′E, 44°04′-46°40′N), a prominent city in Heilongjiang Province, China. Specifically at the urban center within the Second Ring Road (Fig 1). Harbin is a significant political, economic, and cultural center in Northeast China. It is also an important transportation hub in the northeast region. Harbin has undergone significant urbanization, which has resulted in mounting demands for morning peak flow and, in turn, severe traffic congestion. However, as a developing city, Harbin has different travel demands compared to major metropolises like Beijing in terms of population size and economic development levels [39]. This results in both common and unique challenges for Harbin’s bus system. Therefore, it is essential to improve Harbin’s bus network by drawing on the successful experiences of other major cities and tailoring strategies to its own specific circumstances.

According to the Harbin Statistical Yearbook 2023, as of 2022, Harbin had a total of 7,428 buses. The total route length of the bus lines was 8,467 kilometers. The annual passenger volume reached 456.33 million, with an average daily ridership of 1.25 million passengers. There were, on average, 13.5 public buses per ten thousand people.

### 3.2 Data

#### 3.2.1 Bus network data

**Fig 2:**
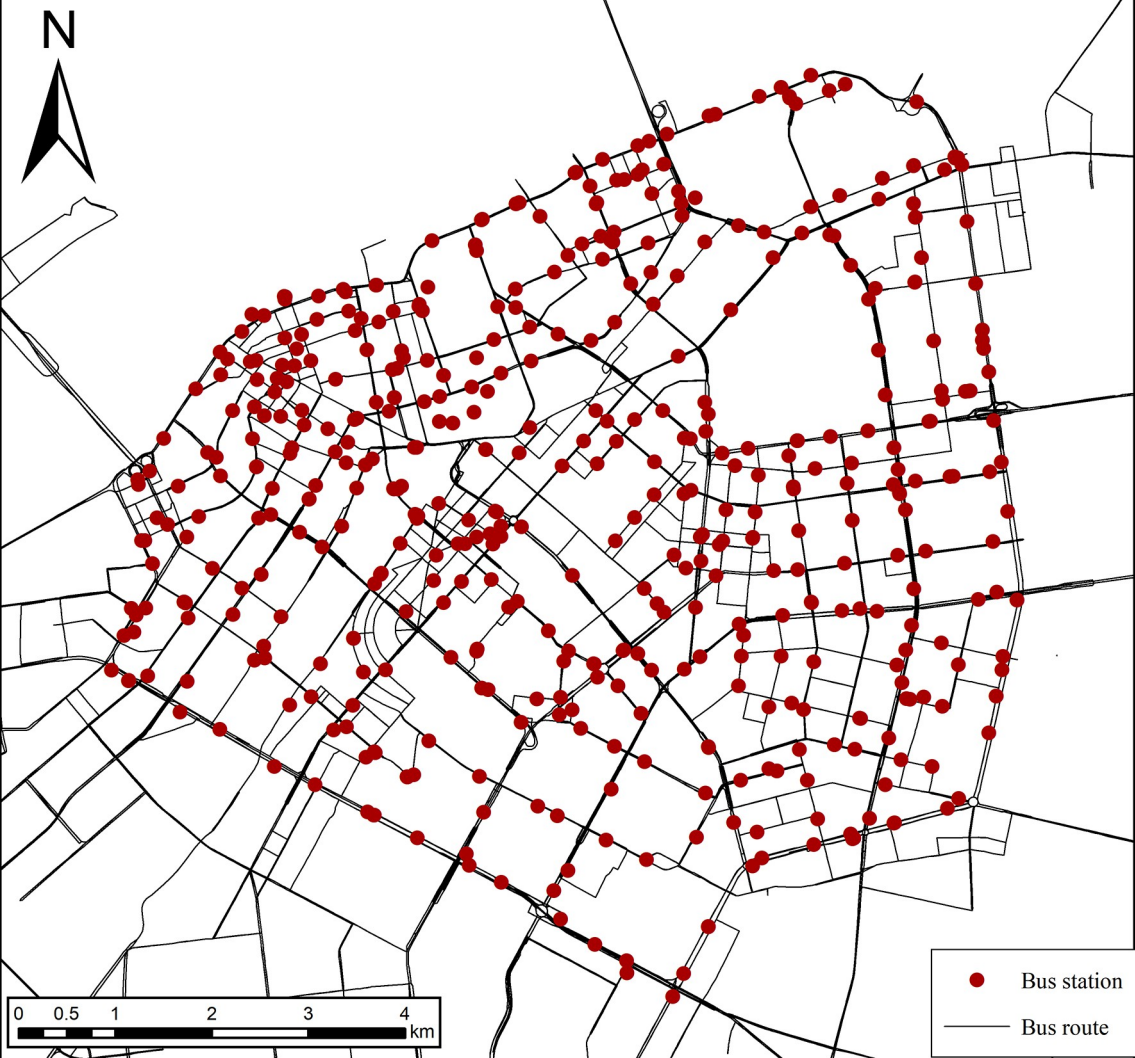
Bus network data. Note: self-drawn image. Source: latitude and longitude from OpenStreetMap.

The bus network dataset utilized in this analysis is sourced from OpenStreetMap (https://www.openstreetmap.org/), which provides vector data on bus stations and bus routes, including: bus name, initial and final departure time, start station, end station, latitude and longitude, number of station, departure intervals, and total route length. A total of 6650 bus stations and 228 bus routes in Harbin were obtained. Given that the focus of this study was on the morning peek period within the second ring road of Harbin, bus stations and bus routes outside the second ring road as well as nighttime bus services were excluded. In addition, we merged bus stations with the same name to obtain a final dataset of 499 bus stations and 201 bus routes (Fig 2). The specific dataset is provided in S1 and S2 Tables. This dataset enables the estimation of bus capacity, thereby providing a representation of the supply side of the bus network.

#### 3.2.2 Population density data

This study utilized Baidu Map (https://map.baidu.com/) to collect real-time heat maps from December 20 to December 24, 2021, during the morning peak period (6:30 a.m.–9:30 a.m.) on a typical workday within Harbin’s Second Ring Road. The data were collected at ten-minute intervals, which is due to the ability to balance the granularity and reliability of the data by capturing detailed features while accounting for network latency and data validity. As part of this, the real-time heat map data reflects the population density of different regions in different colors. More highlighted area in red, higher the population density. In contrast, more highlighted area in blue, lower the population density. The data collected in this study were loaded into ArcGIS 10.2 and classified into six categories according to the color and assigned values from red to blue in ascending order to accurately reflect the demand of the bus network and the utilization of the roads. The specific dataset can be found in S3 Table.

#### 3.2.3 Traffic condition data

Similarly to the real-time population density data, this study uses Gaode Map (https://ditu.amap.com/) to acquire real-time traffic conditions at ten-minute intervals during the morning peak period within Harbin’s Second Ring Road from December 20 to December 24, 2021. The real-time traffic condition map can present various traffic events on the electronic map by different colors, which in turn provides detailed real-time traffic information. Based on the collected information, the colors are classified into four categories: crimson, red, yellow, and green, which represent very congested, congested, slow moving, and smooth, respectively, and are assigned the values of 4, 3, 2, and 1 to represent the real-time traffic conditions of the road, respectively. S4 Table presents the data collection details. The map images themselves are not integral to the composition of this paper. This data was used in this study to determine the traffic congestion in the region where each bus station is located, thus representing the road saturation.

## 4 Method

**Fig 3:**
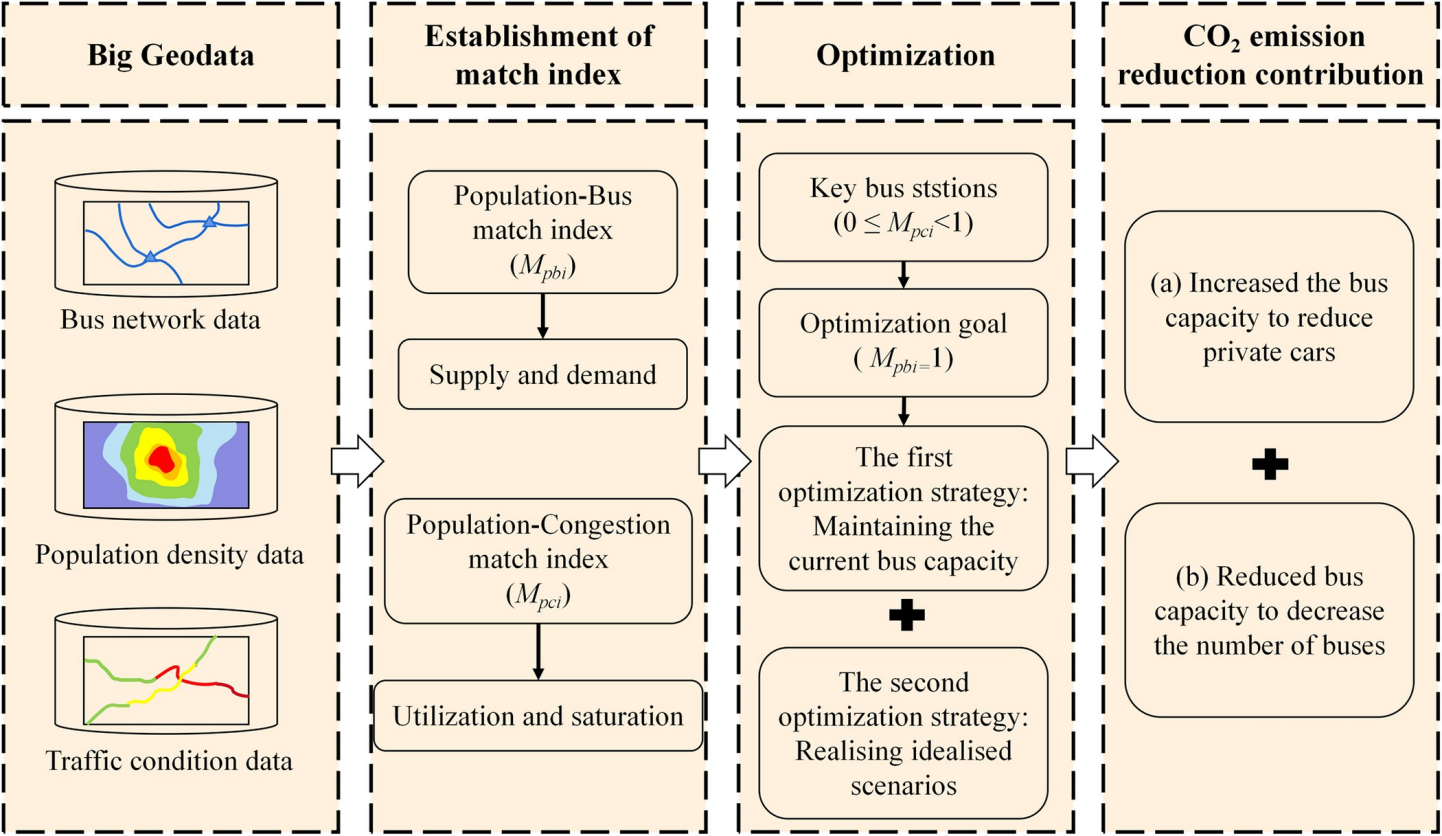
Proposed analysis framework. Note: self-drawn image.

Fig 3 illustrates the proposed analysis framework. First, based on the three datasets, i.e., bus network, population density, and traffic conditions, we establish the Population-Bus Match Index and Population-Congestion Match Index to assess the spatial match degree between supply and demand, utilization and saturation within the bus network [40]. Next, we identify the key bus stations with 0 ≤ *M*_*pci*_<1, which may confront more pressing issues, and residents have strong incentives to choose buses. Subsequently, we introduce two optimization strategies to address the resource allocation mismatch problem. Finally, we estimate the contribution to CO_2_ emission reduction in two scenarios while employing the optimization strategies. All the collected data complied with the terms and conditions of the source of data.

### 4.1 Population-Bus Match Index

This study introduces an index to quantify the matching degree between supply and demand at each bus station to illustrate spatial disparities within local regions. From the supply perspective, we we utilized bus network data to estimate the bus capacity of each bus station within the Second Ring Road during the morning peak period by considering three attributes: bus departure frequency, rated bus capacity, and total number of bus stations on each route. From the demand perspective, we used population density data to quantify the population flow at each bus station by considering the cumulative population flow during the morning peak period.

$$b_{i}=\sum_{j=1}^{l}\left[\frac{Tr_{j}}{s_{j}f_{j}}\right]$$ (1)

$$M_{pbi}=\frac{p_{i}/\sum_{i=1}^{m}p_{i}}{b_{i}/\sum_{i=1}^{m}b_{i}}$$ (2)

where *b*_*i*_ is the bus capacity at the bus station *i*, i.e., the number of passengers carried by the bus during the interval from the time it enters the station to the time it leaves the station, *l* is the number of routes passing from bus station *i*, *j* is the bus route, *f*_*j*_ is the bus departure frequency of route *j* (trips/min), *r*_*j*_ is the rated bus capacity per bus for route *j*, and *s*_*j*_ is the total number of bus stations on bus route *j*. *T* is the morning peak hour (min), *m* is the number of bus stations in the bus network, and *p*_*i*_ is the population flow at bus station *i*. *M*_*pbi*_ measures whether the population flow at bus station *i* effectively allocates the corresponding share of bus capacity. If *M*_*pbi*_ = 1, the share of the population flow at bus station *i* equals the share of the bus capacity, indicating a perfect match between supply and demand. If *M*_*pbi*_ > 1, the population flow share exceeds the bus capacity share at bus station *i*. A high value implies underallocation in this bus station. On the contrary, if 0 ≤ *M*_*pbi*_ < 1, it implies a lower share of population flow at bus station *i* than that of bus capacity. A low value implies overallocation in this bus station.

### 4.2 Population-Congestion Match Index

We also establish an index to quantify the spatial match degree between utilization and saturation at each bus station. From the utilization perspective, we used population density data to measure the utilization of each bus station using the cumulative population flow volume. From the saturation perspective, we used cumulative traffic condition data during the morning peak hours to determine the traffic congestion level in the area surrounding each bus station, thereby representing the saturation of road resources.

$$M_{pci}=\frac{p_{i}/\sum_{i=1}^{m}p_{i}}{c_{i}/\sum_{i=1}^{m}c_{i}}$$ (3)

where *m* is the number of bus stations in the bus network, *p*_*i*_ is the population flow at bus station *i*, *c*_*i*_ is the traffic congestion at bus station *i*, and *M*_*pci*_ measures whether the population flow at bus station *i* is effectively allocated at the corresponding share of traffic congestion. If *M*_*pci*_ = 1, the share of the population flow at bus station *i* equals the share of the traffic congestion. If *M*_*pci*_>1, the population flow share exceeds the traffic congestion share. Conversely, if 0 ≤ *M*_*pci*_<1, the share of the population flow is lower than that of the traffic congestion.

### 4.3 Optimization of bus capacity

The two indexes assess the spatial matching degree between population flow and bus capacity and population flow and traffic congestion. These indexes can identify bus stations with resource allocation mismatches, allowing them to adjust bus capacity effectively and provide a more informed optimization decision-making strategy. Given the limited resource allocation constraints, this work considers that bus stations with 0 ≤ *M*_*pci*_<1 may confront more pressing issues, such as heavy traffic congestion and urgent population flow. Hence, prioritizing these stations allows for a more targeted strategy to address areas in the greatest need of improvement, ultimately leading to a more effective improvement in the overall efficiency of the bus network. Based on this premise, we explore an extreme scenario where the share of bus capacity at each bus station perfectly matches the share of population flow, assuming that all residents choose buses. For this scenario, we propose two optimization strategies to enhance the efficiency of the bus network.

The first optimization strategy has a constraint that the current bus capacity of the bus network remains unchanged. In this strategy, the optimization goal is to achieve *M*_*pbi*_ = 1, where the population share flow at each bus station during the morning peak period equals the share of bus capacity. Additionally, we prioritize bus stations exhibiting a more significant resource allocation mismatch for the optimization strategy. The process is implemented through MATLAB.

$$b_{i}\prime =\underset{M_{\mathrm{pbi}}\to 1}{\lim}\frac{p_{i}\sum_{i=1}^{m}b_{i}\prime }{M_{pbi}\sum_{i=1}^{m}p_{i}}$$ (4)

$$\sum_{i=1}^{m}b_{i}\prime =\sum_{i=1}^{m}b_{i}$$ (5)

where *b*_*i*_*’* is the optimized bus capacity at bus station *i*.

The second optimization strategy strives for an ideal *M*_*pbi*_ = 1 for all key bus stations with resource allocation mismatches. This strategy has no constraints on the current bus capacity of the network. The optimization goal still centers on ensuring that the share of population flow at each bus station perfectly matches the share of bus capacity. Considering that each bus station’s bus capacity affects the current bus capacity, the process is implemented through MATLAB.


$$b_{i}\prime =\underset{M_{\mathrm{pbi}}\to 1}{\lim}\frac{p_{i}\sum_{i=1}^{m}b_{i}\prime }{M_{pbi}\sum_{i=1}^{m}p_{i}}$$ (6)

### 4.4 Estimation of CO_2_ emission reduction contribution based on optimization strategies

In order to explore the potential effect of each strategy on carbon dioxide (CO_2_) emissions reduction, we assess the CO_2_ emissions reduction contribution of each bus station in two scenarios: (1) increased bus capacity to reduce private cars and (2) reduced bus capacity to decrease the number of buses. This study is based on a city-level CO_2_ emission database and employs a fuel-based approach for evaluation.

(1) Increased the bus capacity to reduce private cars: If all the excessive population flow is switched from private cars to buses (private cars with a rated capacity of 5 and buses with a rated capacity of 80), it has the potential to reduce the CO_2_ emissions associated with private cars significantly. The CO_2_ emission reduction contribution at bus station *i* can be calculated as follows:

$$C_{di}=(\frac{{EI}_{pc,gas}\times K_{c}}{R_{pc}}-\frac{{EI}_{cb,die}\times K_{b}\times D}{R_{b}})\times P_{ci}$$ (7)

where *C*_*di*_ represents the CO_2_ emission reduction contribution at bus station *i*, *EI*_*pc*,*gas*_ is the private car energy consumption 0.09L/km, *K*_*c*_ is the CO_2_ emission factor for private cars 2.26 kgCO_2_/L, and *R*_*pc*_ is the rated capacity of private cars. *EI*_*cb*,*die*_ is the diesel bus energy consumption 0.26 L/km, *K*_*b*_ is the CO_2_ emission factor for diesel bus 2.73 kgCO_2_/L, *P*_*ci*_ is the increased bus capacity at bus station *i*, *R*_*b*_ is the rated capacity of diesel bus, *D* is the percentage of diesel buses.

(2) Reduced bus capacity to decrease the number of buses: This study aims to reduce the excess number of buses that operate inefficiently, often due to factors such as running with empty seats. By considering the number of buses cut (rated capacity of 80), the CO_2_ emission reduction contribution at bus station *i* can be derived as follows:

$$C_{di}=\frac{{EI}_{cb,die}\times K_{b}\times D\times P_{di}}{R_{b}}$$ (8)

where *C*_*di*_ is the CO_2_ emission reduction contribution at bus station *i*, *EI*_*cb*,*die*_ is the diesel bus energy consumption 0.26 L/km, *K*_*b*_ is the CO_2_ emission factor for diesel bus 2.73 kgCO_2_/L, *P*_*di*_ is the reduced bus capacity at bus station *i*, *R*_*b*_ is the rated capacity of a diesel bus, and *D* is the percentage of diesel buses.

## 5 Results

### 5.1 Analysis of Population-Bus Matching patterns

Next, we calculate the Population-Bus Match Index for each bus station within the Second Ring Road of Harbin (Eqs (1) and (2)) based on our proposed method.

**Fig 4:**
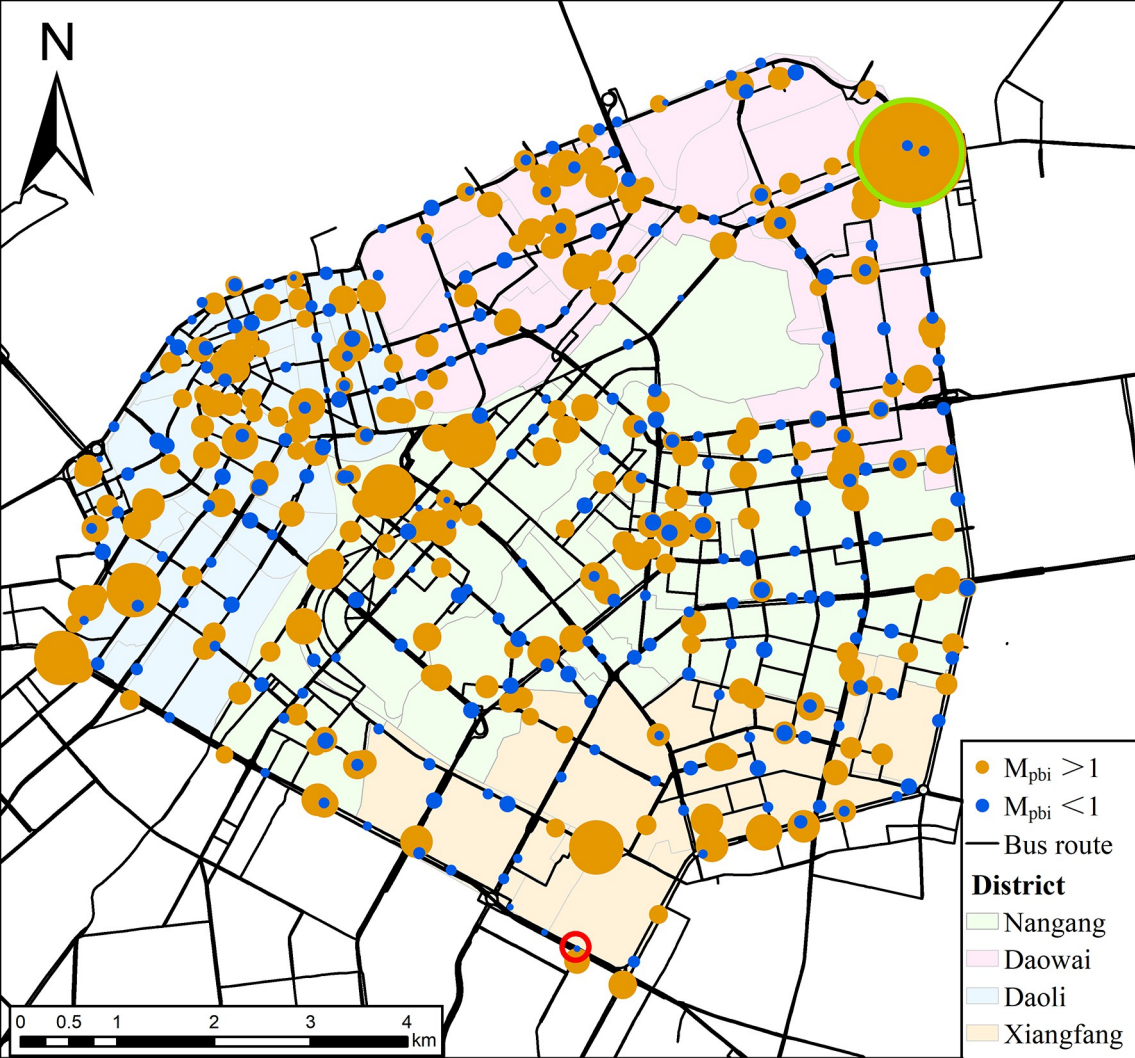
Spatial distribution of Population-Bus Match Index. Note: self-drawn image. Source: latitude and longitude from OpenStreetMap.

Fig 4 illustrates the spatial distribution of the Population-Bus Match Index (*M*_*pbi*_), where the size of the dots represents the magnitude of the *M*_*pbi*_ value for each bus station. The orange dots indicate *M*_*pbi*_>1, signifying that a higher share of the population flow exceeds the bus capacity. The deep blue dots represent *M*_*pbi*_<1, signifying a lower share of population flow compared to the bus capacity. In this study, we divide the study area into four districts according to administration area: Daoli District (Blue), Daowai District (Pink), Nangang District (Green), and Xiangfang District (Yellow).

Based on the analysis of the *M*_*pbi*_ for bus stations during the morning peak period, as illustrated in Fig 4 and Table 1, the following conclusions were drawn: Firstly, the proportion of bus stations with *M*_*pbi*_ > 1 is approximately 61.92%, indicating that most of the population flow is allocated low bus capacity while a minority is allocated large bus capacity. This situation is significant within each district. Among these districts, the Nangang District has the highest proportion of underallocated bus stations, reaching 23.85%. Meanwhile, the Nangang District has the most overallocation of bus stations at 12.83%. This spatial distribution underscores the residents’ challenges in the district, demonstrating a considerable disparity between population flow and the available bus capacity. Secondly, there is a considerable variation in *M*_*pbi*_ values, with the minimum value (0.13) observed at Dianjichang Station (indicated by a green circle) and the maximum value (84.62) at Taiping Second Shop Station (indicated by a red circle). Both stations are located at the intersection of the Second and Third Ring Roads, theoretically serving as critical transportation hubs. However, while Dianjichang Station is serviced by sixteen bus routes, providing sufficient capacity and a relative match between population flow and bus capacity, Taiping Second Shop Station is serviced by only one bus route, resulting in the share of population flow far exceeding the share of bus capacity, thereby failing to meet the commuting needs of the population. Thirdly, the standard deviation of *M*_*pbi*_ in Daowai District is significantly higher than that of the other three districts, indicating a substantial imbalance in bus capacity allocation among the bus stations in this district. This inconsistency is closely linked to the presence of Taiping Second Shop Station, whose *M*_*pbi*_ value of 84.62 greatly skews the district’s overall standard deviation. Further analysis shows that if the data from Taiping Second Shop Station were excluded, the standard deviation of *M*_*pbi*_ in Daowai District would decrease to 2.90, aligning more closely with the standard deviation levels of the other districts, suggesting that the overall bus capacity allocation in Daowai District is not inferior to that of the other districts.

**Table 1:** Statistics on *M*_*pbi*_ of bus stations within four districts.

District	Number of bus station			*M*_*pbi*_			
	*M*_*pbi*_>1	*M*_*pbi*_<1	Total	Mean	Std. deviation	Max.	Min.
**Nangang**	119(23.85%)	64(12.83%)	183(36.67%)	2.97	3.11	18.74	0.14
**Xiangfang**	37(7.41%)	29(5.81%)	66(13.23%)	2.66	3.30	16.18	0.13
**Daoli**	80(16.03%)	44(8.82%)	124(24.85%)	3.32	3.94	22.35	0.17
**Daowai**	73(14.63%)	53(10.62%)	126(25.25%)	3.46	7.81	84.62	0.18
**Total**	309(61.92%)	190(38.08%)	499(100%)	3.10	4.94	84.62	0.13

Note: Percentages are based on all bus stations

### 5.2 Analysis of Population-Congestion Matching patterns

Similarly, we calculate the Population-Congestion Match Index (*M*_*pci*_) for each bus station within the Second Ring Road of Harbin (Eq (3)).

**Fig 5:**
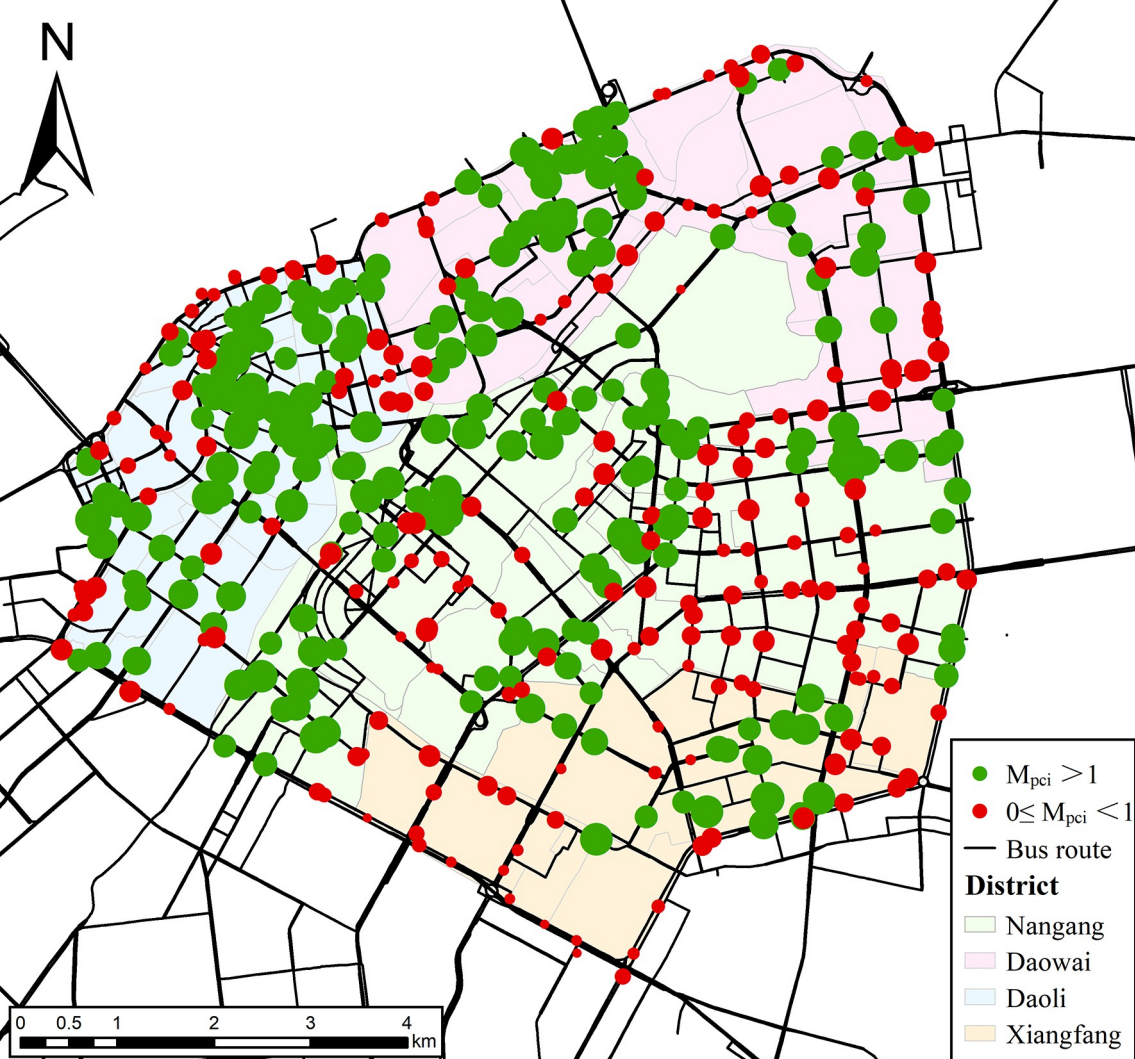
Spatial distribution of Population-Congestion Match Index. Note: self-drawn image. Source: latitude and longitude from OpenStreetMap.

Fig 5 illustrates the spatial distribution of *M*_*pci*_ during the morning peak period, where the size of the dots represents the *M*_*pci*_ value. The dot’s radius increases as the value increases and, conversely, decreases as the value decreases. The dots with *M*_*pci*_>1 are represented in green, signifying a higher share of population flow than traffic congestion. This implies smooth traffic conditions at these bus stations and relatively weaker incentives for residents to choose buses. Conversely, dots with *M*_*pci*_<1 are depicted in red, signifying a higher share of traffic congestion than population flow, which may incentive residents to choose buses.

Based on the analysis of the *M*_*pci*_ data for bus stations in different districts during the morning peak period, as shown in Fig 5 and Table 2, several key characteristics emerge: Firstly, 46.89% of the bus stations experience traffic congestion levels that exceed the current population flow (i.e., 0≤*M*_*pci*_<1). These stations are primarily located at the edges of the Second Ring Road and within the southeastern area inside the Second Ring. Severe road congestion increases travel time and costs for residents, which in turn may lead to increased costs such as congestion charges, parking fees, and fuel taxes [41]. This situation may lead residents of these stations to favor buses as an alternative to alleviate traffic congestion, thereby creating a strong incentive for them to choose buses [39]. Consequently, these stations should be prioritized for optimization in bus capacity allocation to better meet high demand and alleviate traffic congestion. Secondly, from a district-level perspective, Nangang, Daowai, and Daoli districts have more bus stations with *M*_*pci*_ > 1 compared to those with 0≤*M*_*pci*_<1, while Xiangfang is the only district where the opposite is true. This indicates that during the morning peak period, a significant number of bus stations in Xiangfang experience traffic congestion levels that exceed the current population flow. The misalignment between population flow and traffic congestion highlights potential inefficiencies in Xiangfang’s transportation network, emphasizing the urgent need to enhance bus services to better meet residents’ needs. Meanwhile, in Nangang, Daowai, and Daoli, the focus should be on maintaining and improving the positive trends in bus utilization. This will help ensure that the transportation network continues to effectively serve residents and alleviate traffic congestion. Finally, the *M*_*pci*_ values among different bus stations are evenly distributed in the range of 0.29 to 2.21, exhibiting minor variations. The standard deviations of *M*_*pci*_ indicate minimal fluctuations across districts, with most values closely aligning with their averages. This implies a more stable relationship between traffic congestion and population flow at different stations, which helps optimize the allocation of resources to the bus network.

**Table 2:** Statistics on *M*_*pci*_ of bus stations within four districts.

District	Number of bus station			*M*_*pci*_			
	*M*_*pci*_>1	*M*_*pci*_<1	Total	Mean	Std. deviation	Max.	Min.
**Nangang**	95(19.04%)	88(17.64%)	183(36.67%)	1.04	0.34	2.04	0.29
**Xiangfang**	25(5.01%)	41(8.22%)	66(13.23%)	0.90	0.36	1.84	0.31
**Daoli**	77(15.43%)	47(9.42%)	124(24.85%)	1.12	0.36	2.21	0.51
**Daowai**	68(13.63%)	58(11.62%)	126(25.25%)	1.07	0.29	1.69	0.51
**Total**	265(53.11%)	234(46.89%)	499(100%)	1.03	0.34	2.21	0.29

Note: Percentages are based on all bus stations

### 5.3 Explanation of the two optimization strategies

These two matching patterns allow us to understand the current status of the bus network, and specifically, in identifying key bus stations that confront more pressing issues (0≤*M*_*pci*_<1) and thus improving the bus network’s efficiency with targeted strategies, thereby alleviating traffic congestion and reducing CO_2_ emissions. This study introduces two optimization strategies to overcome the mismatch between the share of population flow and the share of bus capacity at the key bus stations, i.e., (0 ≤ *M*_*pci*_ < 1) bus station (Eqs (4)–(6)). Hence, the first optimization strategy considers the current bus capacity unchanged. In this scenario, we identify 234 key bus stations undergoing further optimization. Of these, 66.24% achieve a perfect match (*M*_*pbi*_ = 1) through internal adjustments, i.e., optimization based on the current bus network supply in the study area, with no adjustments to the total amount, while 33.76% of the bus stations remain unchanged. Within the internally adjusted bus stations, 83.23% require an increase in bus capacity, while 16.77% require a reduction. This demonstrates a significant underallocation of bus capacity at these bus stations. The second optimization strategy does not preserve the current bus capacity. Instead, it allows for adjustments to the bus capacity at each station to achieve an idealized scenario with *M*_*pbi*_ = 1 for all bus stations. This strategy results in a 49.57% increase in bus capacity at certain bus stations and a 50.43% reduction at others, with an overall decreasing trend in bus capacity. The bus network’s current bus capacity is excessive, but its allocation at many bus stations cannot match the population flow.

**Fig 6:**
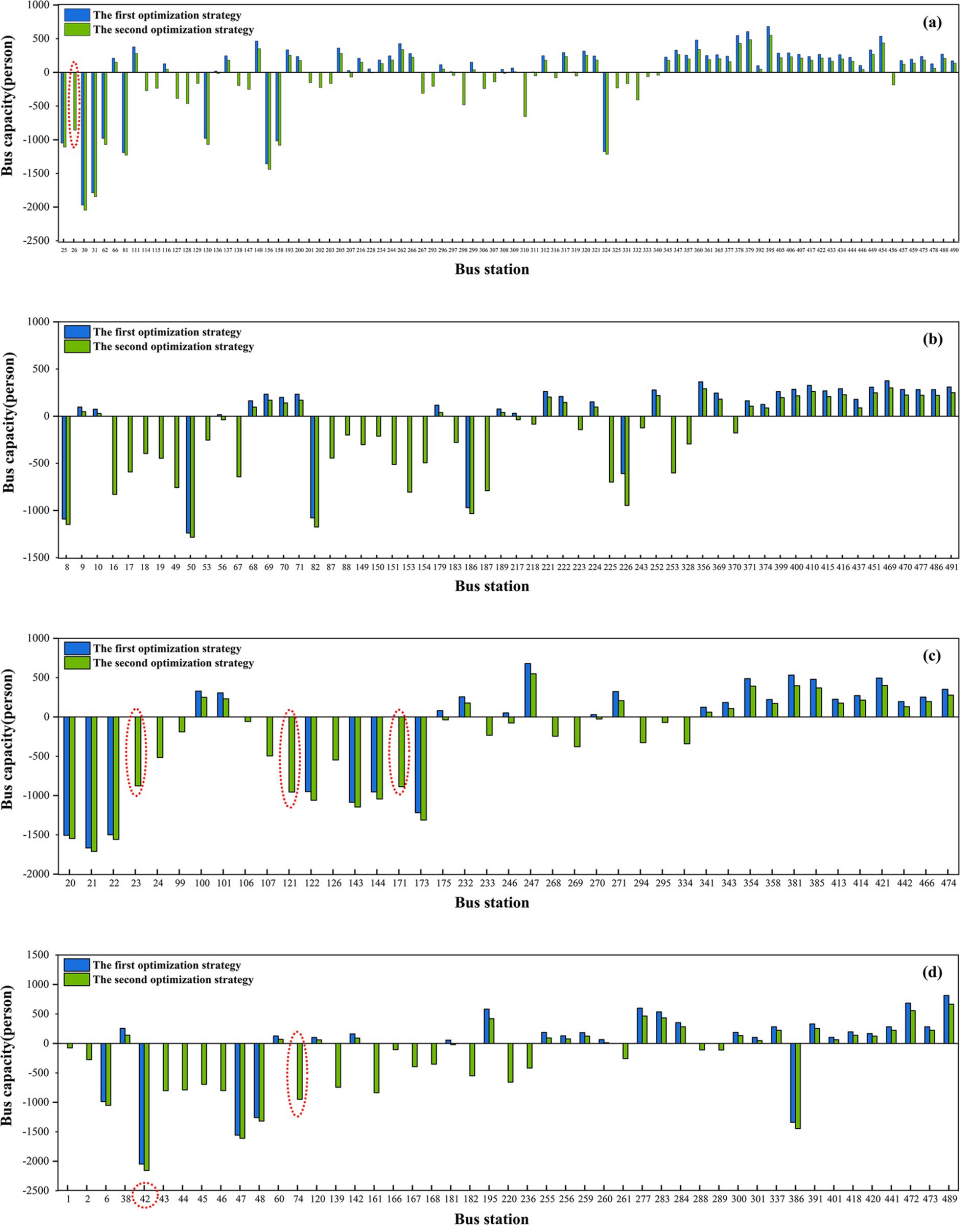
Variations in bus capacity at each bus station. (a) Nangang (b) Daowai (c) Xiangfang (d) Daoli.

Fig 6 illustrates the bus capacity variations at each bus station within the four districts under two optimization strategies. It is worth noting that, under both optimization strategies, the bus station with the greatest change in bus capacity is the Highway Bridge Station in Daoli District (No.42)(marked by the red dashed circle),where the two optimization strategies require reductions in bus capacity of 2047 persons and 2155 persons, respectively, during the morning peak period. This indicates that a certain amount of bus capacity can be reallocated from the Highway Bridge Station to other districts. The five bus stations that exhibit the greatest differences between the two optimization strategies are Gongbin Road(No.121), Anhong Street(No.74), Swan Hote(No.171), Forestry University(No.23), and Harbin Normal University (South Campus) (No. 26)(marked in the red dotted ellipses), where the second optimization strategy reduces the bus capacity by 956, 948, 887, 877, and 859 persons, respectively, relative to the first optimization strategy. This difference is because the five bus stations were not optimized under the first optimization strategy due to the constraint of maintaining the current bus capacity, resulting in their *M*_*pbi*_ not reaching the idealized scenario. However, the second optimization strategy, which results in a perfect match of all bus stations, significantly changes bus capacity at these stations.

Table 3 outlines the changes across different districts following the implementation of the first optimization strategy. This strategy recommends increasing the bus capacity in Nangang District (1,956 persons) and in Daowai District (1,491 persons), while reducing the bus capacity in Xiangfang District (3,010 persons) and in Daoli District (437 persons). Among the adjusted stations, a total of 129 bus stations require an increase in capacity, while 26 stations need a reduction in capacity. Specifically, in each district, the number of bus stations needing increased capacity exceeds those requiring a reduction. Notably, Nangang District needs to increase the capacity of 55 bus stations while reducing the capacity of 9 bus stations. Considering the constraints, the first optimization strategy can only achieve an overall optimization rate of 66.24%, with the highest optimization rate among the four districts being in the Nangang district, where it reaches 72.73%. The high optimization rate in Nangang highlights the strategy’s effectiveness in addressing the specific transportation needs of the district. By strategically reallocating resources, the strategy successfully aligns bus capacity with actual demand. This targeted approach not only improves service efficiency but also demonstrates the potential for tailored optimization strategies to enhance bus systems in varied urban settings.

**Table 3:** Statistics on variations within four districts under the first optimization strategy.

District	The first optimization strategy			
	Optimized stations	Variations in Stations		Bus capacity (persons)
		Increased	Reduced	
**Nangang**	64 (72.73%)	55 (62.50%)	9 (10.23%)	1956
**Xiangfang**	27 (65.85%)	20 (48.78%)	7 (17.07%)	-3010
**Daoli**	29 (61.70%)	24 (51.06%)	5 (10.64%)	-437
**Daowai**	35 (60.34%)	30 (51.72%)	5 (8.62%)	1491
**Total**	155 (66.24%)	129 (55.13%)	26 (11.11%)	0

Note: Percentages are based on the key bus stations

Table 4 reports the overall variations in the four districts following the implementation of the second optimization strategy. As this strategy aims to achieve the desired resource allocation scenario, the optimization rate in all districts is 100%. Overall, 116 bus stations (49.57%) require a reduction in bus capacity, while 118 bus stations (50.43%) need an increase in bus capacity. Despite the nearly equal number of stations requiring increases and decreases in bus capacity, there was an overall reduction in bus capacity across all districts. This indicates a significant misalignment between current bus capacity and actual population flows. The total reduction of 42,989 in bus capacity suggests that the share of existing bus capacity generally exceeds the share of actual population flows, highlighting the need to eliminate excess capacity and more effectively reallocate resources.

**Table 4:** Statistics on variations within four districts under the second optimization strategy.

District	The second optimization strategy			
	Optimized stations	Variations in Stations		Bus capacity (persons)
		Increased	Reduced	
**Nangang**	88(100%)	48 (54.55%)	40 (45.45%)	-9054
**Xiangfang**	41(100%)	17 (41.46%)	24 (58.54%)	-11323
**Daoli**	47(100%)	23 (48.94%)	24 (51.06%)	-11602
**Daowai**	58(100%)	28 (48.28%)	30 (51.72%)	-11010
**Total**	234(100%)	116 (49.57%)	118 (50.43%)	-42989

Note: Percentages are based on the key bus stations

### 5.4 Distribution of CO_2_ emission reduction contribution at bus stations

In order to provide decision-makers with an environmental perspective, this study explores the potential impact of each strategy on CO_2_ emissions reduction. Specifically, we calculate the CO_2_ emissions reduction at each bus station after the implementation of the two optimization strategies (Eq (7)-(8)).

**Fig 7:**
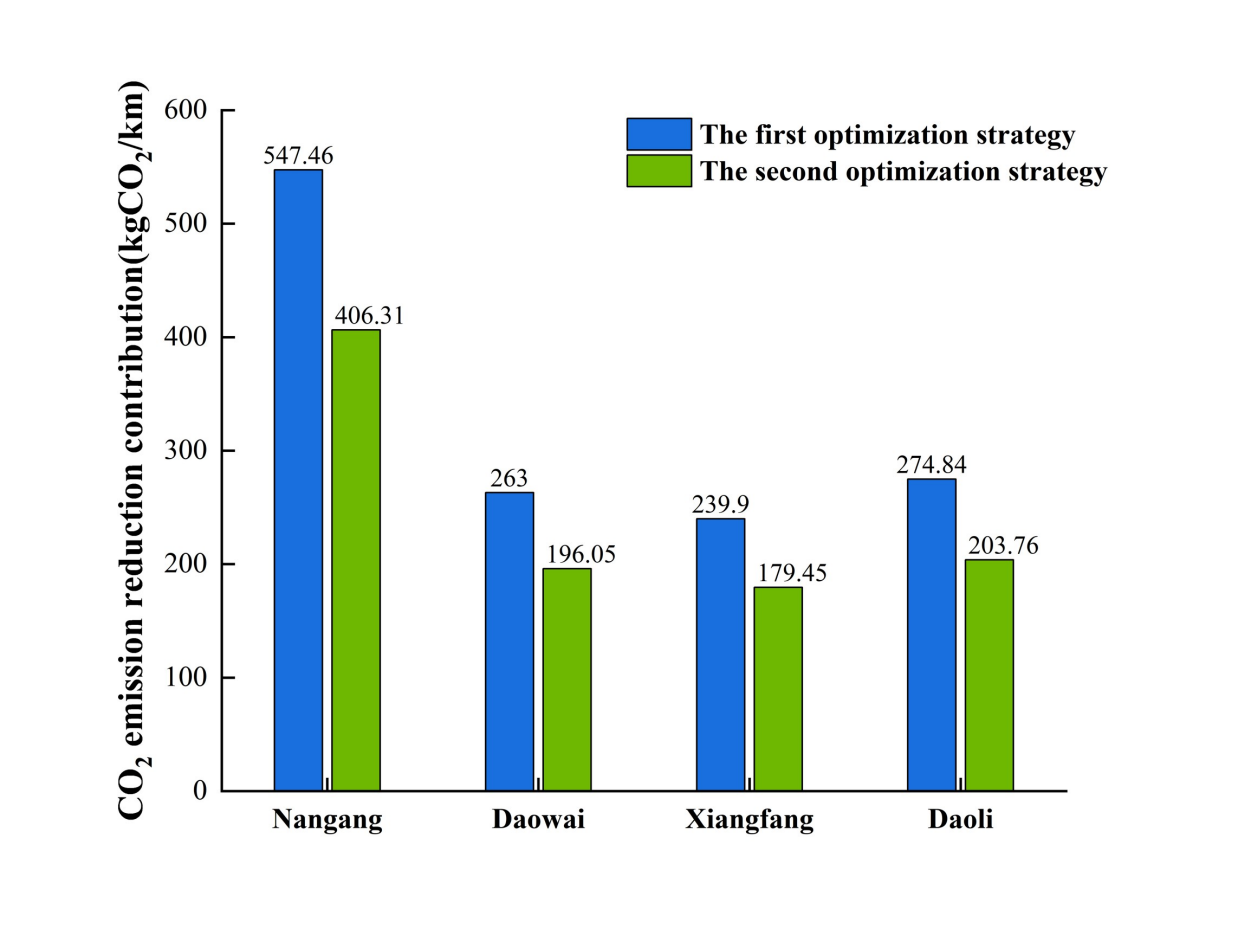
CO_2_ emission reduction contribution within four districts.

The results demonstrate that the total CO_2_ reduction contribution of the first optimization strategy is 1325.20 kgCO_2_/km, and the second optimization strategy contributes 985.57 kgCO_2_/km. This difference underscores the effectiveness of the first strategy in promoting sustainability through improved bus network efficiency. Fig 7 presents the overall CO_2_ emission reduction contributions across four districts for both optimization strategies. The consistent trend across all four districts, where the first strategy outperforms the second, indicates that a targeted approach to optimizing bus capacity can lead to substantial environmental benefits. Additionally, the distribution of CO_2_ emission reduction contributions is similar across the districts, with Nangang District having the highest contribution rate, followed by Daoli District, Daowai District, and Xiangfang District. This indicates that more CO_2_ emissions can be reduced when bus capacity is more accurately matched to actual demand. The largest reduction difference between the two strategies is observed in Nangang, where the first optimization strategy reduces CO_2_ by 141.15 kgCO_2_/km more than the second optimization strategy. The smallest difference is in Xiangfang, where the first strategy reduces CO_2_ by 60.45 kgCO_2_/km more than the second strategy. It is evident that the first strategy has a higher CO_2_ reduction contribution, making it a more effective choice for areas with high population density and high bus demand. The second strategy may be relatively more effective in areas where traffic congestion exceeds the current population flow.

**Fig 8:**
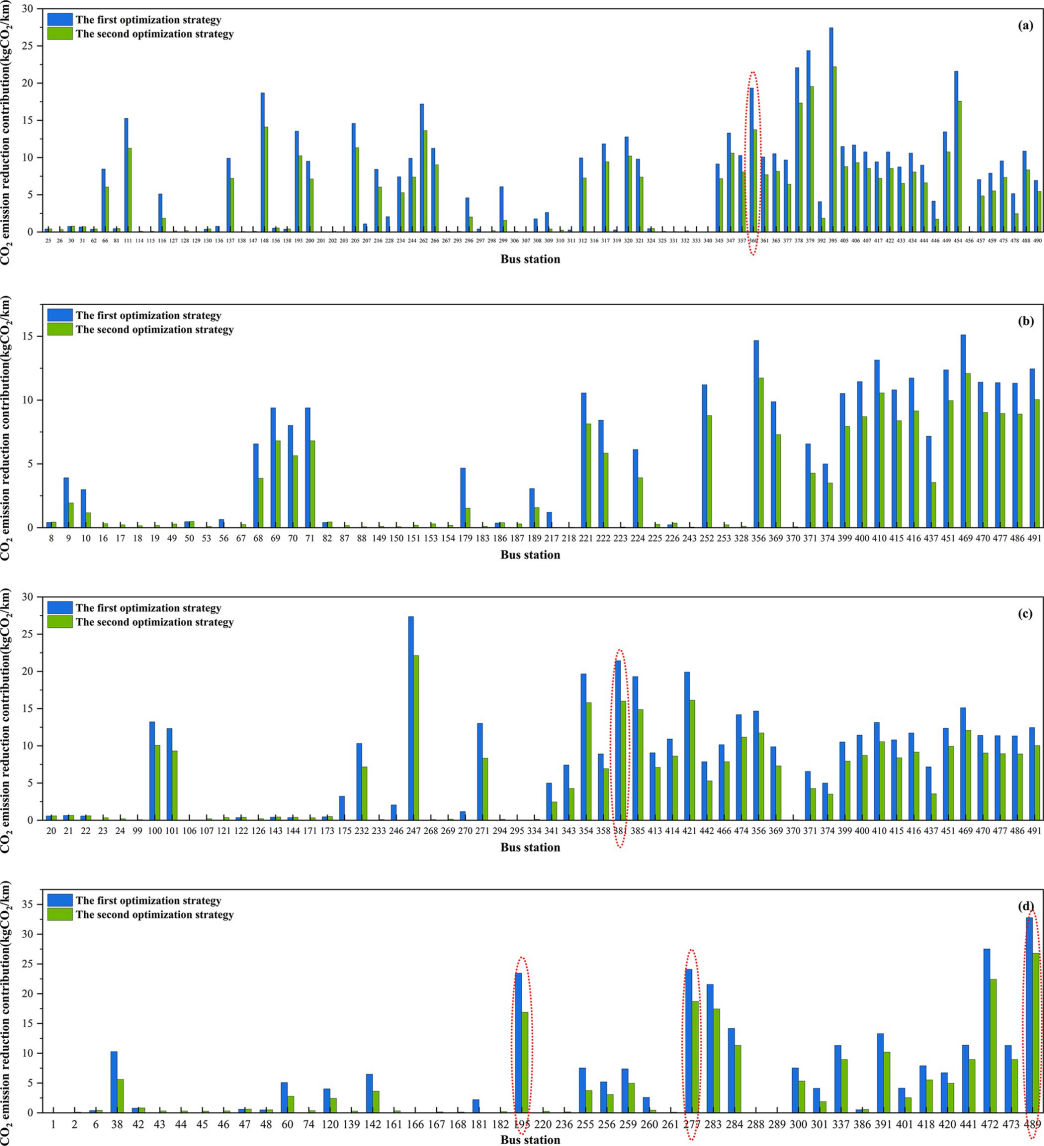
CO_2_ emission reduction contribution at each bus station. (a) Nangang (b) Daowai (c) Xiangfang (d) Daoli.

Fig 8 illustrates the CO_2_ emission reduction contributions of various bus stations across four districts under two optimization strategies. Among these, 55.13% of the bus stations exhibit higher CO_2_ reduction contributions under the first optimization strategy, while 44.87% show higher contributions under the second strategy. In both optimization strategies, the bus stations at Harbin Print Center (No.489) in Daoli District has the highest CO_2_ emission reduction contribution, with the first strategy achieving a reduction of 32.76 kgCO_2_/km and the second strategy achieving 26.80 kgCO_2_/km. Additionally, the bus stations with the largest differences in CO_2_ emission reduction contributions are Wujing Hospital (No.195), Harbin Print Center (No.489), Litchi Street (No.360), Zhongxin Department Store (No.381), and Steel Residential Area (No.277), as indicated by red dashed lines. At these five stations, the first optimization strategy consistently yields higher contributions. Decision-makers can prioritize implementing the first optimization strategy at these key stations to maximize CO_2_ emission reduction benefits.

## 6 Discussion

The optimization of urban bus networks is essential for enhancing public transportation efficiency, reducing congestion, and minimizing environmental impacts [42]. This study focused on the spatial matching of supply and demand in Harbin’s bus network, proposing two distinct optimization strategies. The results provide valuable insights into potential improvements and highlight key areas for further research and practical implementation.

Several studies highlight the inefficiencies in urban bus networks caused by mismatches between supply and demand. For example, Liu et al. [43] and Shang et al. [44] both identified similar issues in their analyses of bus networks in other Chinese cities, noting that optimizing bus routes and schedules based on real-time data can significantly improve efficiency and reduce congestion. The results of this study, particularly the high levels of underallocation in districts like Nangang, echo these studies’ conclusions about the critical need for better alignment between bus supply and passenger demand. Our two optimization strategies demonstrated distinct benefits and limitations. The first optimization strategy, constrained by the current bus capacity, focuses on internal adjustments and demonstrated a potential to enhance efficiency through targeted reallocations. This approach aligns well with strategies used in other studies, such as those by Wei et al. [14] and Wang et al. [45], which also emphasize internal reallocations to optimize bus routes without significantly altering the existing infrastructure. The second strategy, which allows for adjustments beyond the current bus capacity, aims for an idealized scenario with perfect matches across all stations. This strategy is more ambitious and comprehensive, similar to approaches proposed by Wang [46], which seek to maximize service coverage and efficiency through significant modifications to bus networks. In conclusion, both optimization strategies have distinct advantages. For policymakers, targeted reallocations can bring significant improvements in the short term, while comprehensive modifications can create a more efficient and sustainable transportation system in the long run. This dual approach ensures that immediate needs are met while laying the foundation for future enhancements. The study also underscores the value of incorporating environmental factors into transportation planning, demonstrating that optimized resource allocation can significantly reduce CO_2_ emissions. Chen et al. [47] conducted an empirical analysis assessing the low-carbon friendliness of the road network structure in the central urban area of Shanghai. Similar to this study, both propose low-carbon-oriented optimization strategies, emphasizing the importance of incorporating environmental sustainability into urban transportation planning to achieve significant carbon reduction and improved efficiency.

While our overall results were consistent with expectations, some unexpected findings warrant further discussion. Notably, the CO_2_ emission reduction contributions were higher in the first optimization strategy despite its more conservative approach. This contrasts with studies like those by Estrada et al. [48] and Dong et al. [49], which suggest that more extensive changes (as in our second strategy) typically yield greater environmental benefits. The higher reduction in CO_2_ emissions under the first strategy can be explained by the fact that increasing bus capacity at specific high-demand stations more effectively attracts private car users to switch to buses, thus reducing emissions more significantly than a blanket capacity reduction approach.

## 7 Conclusion

In this study, two optimization strategies based on spatial matching patterns were proposed to address inefficiencies in Harbin’s urban bus network. The first optimization strategy, constrained by the current bus capacity, focused on internal adjustments, showing potential to enhance efficiency through targeted reallocations. The second optimization strategy, which allowed for adjustments beyond current capacity, aimed for an idealized scenario with perfect matches across all stations. Both strategies demonstrated distinct benefits and limitations, providing valuable insights into the optimization of urban bus networks. The results highlighted the critical need for aligning bus supply with passenger demand based on spatial matching, as well as the environmental benefits of optimized resource allocation. The major findings are as follows.

Firstly, during the morning peak period, there is a significant mismatch between supply and demand across bus stations, with more stations experiencing underallocation of bus capacity (i.e., population flow exceeding the corresponding share of bus capacity) than overallocation. On a local scale, Nangang District exhibits a particularly severe mismatch between bus capacity and population flow proportions. Notably, Taiping Second Shop Station has an extremely high *M*_*pbi*_ value, significantly impacting the overall bus capacity distribution in Daowai District, highlighting the supply-demand imbalance at this station.

Secondly, during the morning peak period, 46.89% of bus stations experience traffic congestion exceeding population flow, particularly around the Second Ring Road. This indicates a need to optimize bus capacity allocation in these areas. Xiangfang district, with more stations showing severe congestion relative to population flow, requires urgent enhancement of bus capacity to address inefficiencies. In contrast, Nangang, Daowai, and Daoli should focus on maintaining and improving positive trends in bus utilization.

Third, the first optimization strategy achieves a perfect match (*M*_*pbi*_ = 1) for 66.24% of the key bus stations under the constraint of maintaining existing bus capacity. Among these stations, the majority require an increase in bus capacity, while a few need a reduction. This strategy improves the match degree at most key stations without requiring a complete overhaul of the existing bus capacity. However, working within fixed capacity constraints limits the strategy’s ability to fully address all areas with misaligned bus services. The second optimization strategy, based on ideal conditions, achieves a perfect match for 100% of the stations by dynamically adjusting bus capacity. In this scenario, nearly half of the stations see an increase in bus capacity, while the other half experience a decrease, ultimately resulting in an overall reduction in bus capacity.This strategy ensures comprehensive optimization, making sure all key stations receive an appropriate level of bus capacity. However, it also necessitates significant adjustments across the entire network, which could be resource-intensive and disruptive. Additionally, during implementation, challenges may arise, particularly in maintaining service consistency during the transition period.

Lastly, after implementing the two optimization strategies, the overall CO_2_ emission reduction contribution rate is higher with the first strategy. Nangang District exhibits significant potential for reducing CO_2_ emissions. Additionally, it is noteworthy that the Harbin Print Center in Daoli District has the highest contribution rate under both strategies. This indicates that the station plays a crucial role in achieving the city’s CO_2_ emission reduction goals.

The research conclusions and proposed optimization strategies are based on a rigorous analysis of current public transportation data, focusing on spatial matching patterns between bus capacity and population flow. This research acknowledges that factors such as economic development levels, transportation infrastructure, and weather conditions play a crucial role in influencing public transportation usage. While these factors were not explicitly modeled in this study, the proposed optimization strategies are grounded in empirical data and are designed to be adaptable to different urban environments. The conclusions drawn are based on a reliable analysis of population flow and bus network efficiency, providing a foundational framework. Future research can build on this framework by incorporating additional variables to ensure that the strategies remain flexible and applicable across various scenarios, thereby enhancing their generalizability and effectiveness. Moreover, this study analyzes the optimization strategies of Harbin’s bus network based on data from December 20 to 24, under non-rainy and non-snowy weather conditions. We recognize that Harbin’s seasonal variations and weather conditions significantly impact traffic conditions and residents’ travel choices. Although the data selection in this study effectively avoids short-term disturbances caused by rainy or snowy weather, to further validate the applicability and robustness of the optimization strategies, future research will expand to include traffic data from different seasons and weather conditions. Through comparative analysis, we will assess the effectiveness of the optimization strategies under a broader range of weather conditions and time periods, ensuring the general applicability and scientific validity of the research conclusions.

## Supporting information

S1 Table Bus routes data. (PDF)

S2 Table Bus stations data. (PDF)

S3 Table Population density data. (PDF)

S4 Table Traffic condition data. (PDF)
