# Supplementary material for: Optimizing urban bus network based on spatial matching patterns for sustainable transportation: A case study in Harbin, China
Source: PLoS One. 2024 Oct 28;19(10):e0312803. doi: 10.1371/journal.pone.0312803 (PMC11515997; doi:10.1371/journal.pone.0312803)
Supplement: S1 Table — (PDF) [file pone.0312803.s001.pdf]

S1 Table. Bus route data

Number	Bus route	Initial and final departure time	Longitude of starting station	Latitude of starting station	Longitude of the terminal station	Latitude of the terminal station	Total stations	Departure interval (minutes)	Total route length
1	1	05:30-22:00	126.607918	45.771835	126.680664	45.799969	19	4	9.04307
2	10	05:30-21:00	126.701546	45.690849	126.64386	45.776821	24	5	15.53747
3	11	05:00-22:00	126.636871	45.757515	126.579026	45.70448	22	5	11.97206
4	12	05:30-21:00	126.526688	45.735729	126.685287	45.799206	37	4	17.47019
5	13	05:30-21:30	126.60331	45.770382	126.703064	45.770935	20	5	12.68549
6	14	05:30-21:30	126.589638	45.749969	126.712265	45.757565	28	3	17.58429
7	15	06:00-21:00	126.620064	45.776672	126.49944	45.719563	24	5	14.30132
8	16	05:30-20:30	126.708359	45.744976	126.611431	45.777867	28	4	12.99751
9	17	05:30-20:30	126.730934	45.718281	126.608421	45.761875	30	5	17.55225
10	18	05:30-20:00	126.671158	45.694199	126.643921	45.757015	19	5	11.44323
11	19	05:00-20:00	126.753677	45.774437	126.637306	45.772678	24	5	13.95696
12	101	05:00-21:30	126.686157	45.709385	126.630531	45.779133	19	3	12.6599
13	102	05:00-21:30	126.674995	45.711639	126.630302	45.77956	22	2	13.06917
14	103	05:30-22:00	126.607201	45.7761	126.703094	45.732868	19	3	12.67268
15	104	05:30-22:00	126.676521	45.783035	126.624115	45.658497	28	2	15.86185
16	105		126.526001	45.742565	126.710548	45.782818	29	5	17.18052
17	106	05:40-20:30	126.634041	45.678894	126.66011	45.781124	30	5	16.07562
18	107	05:30-21:30	126.667236	45.795898	126.645363	45.679749	34	3	18.55252
19	108		126.600754	45.979805	126.523239	45.874962	30	5	17.72022
20	109		126.658577	45.790665	126.651352	45.740074	20	5	10.8077
21	111（Circle）	06:30-20:00	126.571335	45.735863	126.571259	45.735859	34	4	18.81885
22	112	06:00-20:00	126.705269	45.724606	126.627302	45.760218	27	5	14.69819
23	113		126.522278	45.717316	126.750717	45.774063	39	5	21.94016
24	114	07:00-17:00	126.624115	45.658516	126.635727	45.653099	3	4	1.53018
25	115	06:30-20:00	126.644073	45.756607	126.70253	45.732075	11	5	7.18383
26	116	06:00-20:30	126.594353	45.753468	126.737251	45.742458	39	4	23.31013
27	117	05:30-20:30	126.5467	45.712059	126.704536	45.732849	36	5	20.5621
28	118	06:00-20:00	126.60723	45.776097	126.691795	45.679951	29	5	16.79125
29	119	06:00-22:30	126.640121	45.752029	126.560455	45.819565	23	5	18.1381
30	120	06:00-20:00	126.710403	45.782703	126.579497	45.704193	38	8	23.64203
31	121	06:00-20:30	126.641013	45.679213	126.718254	45.789341	38	5	22.43743
32	122	05:30-20:00	126.50872	45.825626	126.659764	45.780427	42	10	25.80673
33	123	06:30-20:00	126.507988	45.82655	126.653198	45.824432	32	10	23.44349
34	124		126.579155	45.70409	126.549736	45.723915	23	8	13.53699
35	125	06:00-21:00	126.495636	45.81778	126.545647	45.7089	39	6	26.29307
36	126	06:30-22:30	126.619568	45.778465	126.466125	45.822243	24	6	19.03419
37	127	05:30-21:30	126.580162	45.821781	126.661823	45.726311	44	8	27.12405
38	128	06:00-21:00	126.620277	45.778542	126.644789	45.679521	30	6	16.8163
39	129		126.50798	45.826492	126.576157	45.709827	32	8	26.4376
40	130	07:00-19:00	126.651665	45.774502	126.578629	45.704592	21	8	12.66336
41	131		126.54277	45.693649	126.630433	45.77938	31	8	17.23327
42	132		126.660193	45.781123	126.526339	45.73513	34	12	17.3868
43	136		126.594711	45.752979	126.750389	45.774364	30	8	17.04575
44	150	06:30-18:30	126.630623	45.715981	126.551122	45.655993	9	60	11.68993

45	2	05:00-21:30	126.703598	45.727905	126.611547	45.777848	21	5	13.22521
46	20	05:30-21:30	126.62973	45.779163	126.549812	45.689022	22	3	14.2642
47	21	06:00-20:30	126.604134	45.771851	126.725914	45.731743	24	5	15.80209
48	22	06:00-20:00	126.593903	45.754711	126.707413	45.688396	26	5	17.77635
49	23		126.700531	45.741413	126.609261	45.771816	20	4	12.48582
50	24	06:00-21:00	126.608559	45.761581	126.703407	45.727875	35	5	19.73193
51	25	06:00-20:00	126.707237	45.767105	126.509384	45.719814	40	6	25.27234
52	26	06:00-20:00	126.623085	45.782936	126.708572	45.72905	26	5	15.01257
53	27	05:48-20:00	126.735527	45.793179	126.67968	45.72237	25	5	15.09957
54	28	06:00-20:30	126.653191	45.784786	126.643875	45.695934	24	5	15.73757
55	29	06:30-20:00	126.586838	45.82233	126.627792	45.760479	29	8	20.79512
56	201		126.564041	45.721615	126.698105	45.742241	40	6	21.75274
57	202	05:10-21:00	126.661537	45.796673	126.663734	45.688229	32	6	17.34817
58	203	06:00-20:30	126.588875	45.683983	126.710052	45.783951	41	6	26.02538
59	205	06:00-20:30	126.629814	45.779449	126.65834	45.689377	40	5	21.41204
60	206	05:30-21:20	126.579819	45.746014	126.718277	45.685226	49	5	29.87511
61	209		126.711342	45.764252	126.525993	45.744274	55	4	30.07562
62	210	06:30-17:30	126.676384	45.593708	126.607292	45.596489	25	8	13.23551
63	211		126.584442	45.903744	126.594368	45.75975	31	8	24.23076
64	212		126.568542	45.912292	126.595352	45.758366	28	8	23.46923
65	213		126.49054	45.875061	126.594261	45.759899	24	5	19.79157
66	215	06:30-20:00	126.595831	45.758072	126.640922	45.848017	20	10	21.53247
67	216		126.495491	45.815861	126.59391	45.75835	19	30	15.33729
68	217	05:15-18:30	126.742363	45.691612	126.590729	45.732635	44	8	25.45644
69	218		126.646202	45.680031	126.51078	45.735802	42	6	21.08209
70	219		126.523216	45.869518	126.594482	45.759602	22	10	19.03591
71	220	06:00-19:00	126.606926	45.594589	126.561821	45.74305	43	10	25.92106
72	223		126.584442	45.903755	126.595222	45.758549	31	60	26.11647
73	225		126.597504	45.759663	126.623177	45.890347	32	8	24.79322
74	226		126.594421	45.758545	126.523346	45.883324	20	15	21.01226
75	3	05:00-21:30	126.690087	45.725971	126.66227	45.795441	22	7	13.4451
76	30	06:00-20:30	126.709152	45.787254	126.656631	45.696148	27	5	14.39604
77	31	05:30-21:00	126.579317	45.704415	126.711708	45.768139	31	5	17.48342
78	32	05:30-20:30	126.520424	45.718113	126.639778	45.756271	27	4	13.99205
79	33		126.576262	45.709343	126.707819	45.78903	35	5	21.74677
80	34	06:00-18:00	126.691826	45.679821	126.682281	45.74057	19	5	9.7846
81	35	06:30-21:30	126.709305	45.78664	126.500504	45.808266	38	6	25.59701
82	35（Interval）	06:30-18:30	126.68116	45.787674	126.645172	45.843372	7	10	8.3172
83	36	05:30-20:30	126.627709	45.761122	126.682899	45.828533	17	5	13.06961
84	37	06:00-20:00	126.69487	45.797768	126.576157	45.709827	31	5	21.27674
85	39		126.661859	45.647307	126.689598	45.725468	28	15	16.03972
86	301	07:00-10:00	126.631561	45.75861	127.127945	45.388512	2	20	61.65346
87	331		126.680298	45.785286	126.802734	45.857288	33	60	15.65595
88	332	05:30-19:00	126.822968	45.784145	126.707893	45.789207	19	10	10.94877
89	333		126.67424	45.587139	126.690245	45.725758	34	15	24.40343
90	334		126.953804	45.727608	126.676659	45.786003	60	10	33.73688
91	335	05:00-19:00	126.600235	45.747929	126.199455	45.630737	55	10	37.96003
92	336	05:10-19:00	126.541508	45.660014	126.630898	45.757393	26	5	15.33016
93	337		126.82323	45.623438	126.690058	45.726139	24	10	17.29573
94	338		126.623734	45.600098	126.62999	45.757511	35	5	24.68985

95	338（Interval）	06:30-19:00	126.646729	45.719036	126.683327	45.613075	8	10	13.17417
96	339		126.676491	45.785942	126.980164	45.94342	50	10	38.44522
97	340	05:00-20:00	126.836569	45.700469	126.690514	45.726891	28	15	13.75635
98	341	05:00-17:30	126.344734	45.661667	126.598412	45.747169	47	10	24.74212
99	343	05:00-20:00	126.674175	45.586684	126.62941	45.756855	30	6	24.84071
100	345	05:00-18:00	126.389876	45.544098	126.599525	45.747181	47	10	33.02056
101	346		126.599442	45.755141	126.348434	45.794659	33	5	33.4979
102	348	05:30-20:00	126.613747	45.606422	126.608788	45.761681	33	6	24.65952
103	349	06:00-17:00	126.611832	45.704315	126.555901	45.66954	12	20	7.48708
104	352	06:15-20:05	126.680275	45.785278	126.929901	45.732872	43	90	22.5342
105	353	05:40-18:00	126.820206	45.728508	126.690514	45.726879	31	15	12.35821
106	355	06:00-18:00	126.924591	45.872993	126.677025	45.786068	52	15	25.67383
107	356		126.951988	45.716297	126.65303	45.793056	48	8	32.62198
108	357	06:00-20:00	126.788368	45.779106	126.642288	45.777462	24	7	14.1887
109	358		126.676323	45.785908	126.921562	45.833984	38	60	25.33912
110	359	06:00-18:00	126.657906	45.788071	126.771935	45.873123	46	15	25.25215
111	360	05:00-17:00	126.75898	45.86668	126.680725	45.785629	35	15	17.75036
112	361	06:00-19:30	126.636391	45.581429	126.616768	45.606983	22	8	9.85159
113	362	05:00-18:00	126.463715	45.593426	126.593323	45.756142	41	8	24.94137
114	363		126.59333	45.756142	126.542267	45.660728	31	4	16.53251
115	364	06:00-19:00	126.598694	45.70673	126.738861	45.700191	41	30	23.84275
116	365	06:00-20:00	126.707428	45.764423	126.677948	45.675106	28	4	12.9019
117	366	06:00-19:30	126.690483	45.725803	126.748154	45.738018	14	10	6.5122
118	367	06:00-19:00	126.718437	45.789555	126.690521	45.726868	29	8	16.16457
119	368	05:30-19:00	126.799408	45.703209	126.690514	45.726658	26	8	11.1355
120	369	06:00-18:30	126.481255	45.696362	126.611732	45.704304	36	8	22.46413
121	371		126.607849	45.594345	126.688454	45.72514	47	5	25.8046
122	375	05:00-18:40	126.80027	45.871361	126.680641	45.785603	43	10	17.49607
123	376	06:00-19:00	126.702499	45.758266	126.525993	45.743763	34	4	20.21512
124	377	05:00-20:00	126.836388	45.700352	126.690514	45.726891	30	10	13.71869
125	378	07:00-18:00	126.769318	45.702629	126.690567	45.728085	18	60	9.44135
126	379	06:00-19:50	126.75875	45.71645	126.578942	45.704161	39	5	21.73251
127	380		126.67691	45.786041	126.953781	45.727638	46	60	29.63354
128	381	06:00-19:30	126.552597	45.673447	126.550064	45.624489	11	10	6.50162
129	382	06:10-18:10	126.611893	45.704319	126.46627	45.542046	32	20	23.7393
130	383	04:38-18:00	126.312363	45.634285	126.641357	45.758015	49	8	32.33541
131	385		127.005348	45.740395	126.658028	45.788116	54	0	35.76908
132	386	05:30-18:30	126.858967	45.745952	126.638	45.755959	34	8	23.68038
133	390	07:00-17:00	126.690567	45.727821	126.807487	45.736118	38	40	17.96464
134	396	06:05-19:30	126.594505	45.756542	126.344238	45.929871	38	60	36.20834
135	398	06:00-18:00	126.853081	45.851971	126.677048	45.786102	44	60	23.70171
136	399	06:30-18:30	126.653793	45.604332	126.641556	45.572632	25	6	11.93908
137	4	05:30-21:00	126.611512	45.777599	126.718057	45.797223	19	6	10.76096
138	40	07:00-18:00	126.642311	45.777462	126.765289	45.736702	18	15	12.97523
139	44	05:30-20:30	126.707237	45.767097	126.622131	45.714458	27	8	15.66326
140	46		126.533806	45.720821	126.70285	45.770912	35	8	20.57268
141	47		126.640419	45.758675	126.489494	45.786221	32	6	22.48422
142	48		126.662254	45.795456	126.638054	45.81237	14	10	11.90378
143	49	07:30-18:00	126.618279	45.800457	126.61866	45.817097	4	120	2.40151
144	5	05:30-21:30	126.702866	45.763088	126.561127	45.716747	30	6	18.96735

145	50	07:30-18:00	126.582954	45.835022	126.618568	45.817215	6	30	4.72786
146	51		126.585182	45.709053	126.705368	45.788544	47	5	24.90628
147	53	05:30-20:30	126.606117	45.768715	126.763771	45.730484	34	6	22.64717
148	55		126.707353	45.774178	126.69204	45.679986	42	4	22.87855
149	56	06:00-20:30	126.652313	45.779823	126.674942	45.706348	34	5	19.85414
150	57		126.516838	45.720711	126.701157	45.725964	45	5	28.71462
151	58	06:00-18:30	126.70694	45.763676	126.565887	45.69698	33	8	19.60364
152	59	05:50-20:30	126.542563	45.693542	126.709084	45.73196	33	5	20.33029
153	551	06:20-20:00	126.629566	45.756723	126.609215	45.982529	17	10	33.69714
154	552	05:00-19:20	126.609314	45.98418	126.590935	45.759243	19	15	29.86717
155	553		126.707367	45.752216	126.609283	45.982288	18	15	33.02774
156	6	05:00-21:30	126.707413	45.789165	126.627686	45.760685	15	6	10.12396
157	60	06:00-20:00	126.707169	45.691776	126.648224	45.79287	28	6	16.2787
158	61	05:50-20:30	126.503174	45.71891	126.700073	45.765369	51	6	27.1441
159	62	06:00-20:00	126.757225	45.756004	126.706696	45.688072	38	6	24.38324
160	63		126.526001	45.742527	126.680801	45.757435	35	4	18.76755
161	64		126.577934	45.696064	126.665741	45.788208	39	3	19.08947
162	65		126.593704	45.754345	126.709419	45.788139	26	5	13.98196
163	66		126.593742	45.754307	126.73616	45.792885	23	5	15.09303
164	67		126.611473	45.771461	126.66626	45.691601	40	5	23.34911
165	68	05:30-20:30	126.624107	45.659569	126.711945	45.752373	38	3	20.98646
166	69		126.760696	45.748833	126.597992	45.688206	49	5	28.43295
167	601	06:00-20:00	126.628761	45.756123	126.966125	45.544762	4	10	40.18563
168	602	06:40-18:30	126.578407	45.704329	126.966125	45.544762	9	15	42.87813
169	603	06:00-17:00	126.70031	45.74136	126.968765	45.539387	29	20	43.40411
170	7	05:30-20:30	126.699974	45.741486	126.647758	45.793324	24	5	12.8352
171	70	06:00-20:30	126.705437	45.722778	126.641716	45.789417	26	5	13.23157
172	71		126.707069	45.781525	126.514427	45.718052	44	6	24.321
173	72	06:00-20:00	126.677673	45.693481	126.667931	45.793884	30	6	17.08856
174	73	06:00-20:30	126.739426	45.781681	126.695816	45.724728	51	6	28.20048
175	74		126.673668	45.74556	126.59272	45.760357	33	3	23.31869
176	75		126.607345	45.775975	126.673172	45.70284	31	5	14.80761
177	76	06:00-21:00	126.629831	45.779406	126.727654	45.711533	40	5	22.5935
178	77		126.757461	45.756699	126.593742	45.753986	41	5	23.27415
179	78	06:00-20:30	126.714523	45.753063	126.587822	45.736603	31	6	16.79595
180	79	05:30-20:30	126.728188	45.830868	126.5924	45.741898	36	6	19.215
181	8	05:30-21:00	126.70842	45.746246	126.606964	45.775931	26	4	15.09852
182	80		126.43795	45.844341	126.594437	45.759659	23	10	19.26509
183	81	06:00-20:00	126.63102	45.661274	126.633171	45.757118	25	5	16.49409
184	82		126.560043	45.719002	126.705986	45.763435	36	5	18.72698
185	83	06:00-20:30	126.623558	45.783092	126.588791	45.684074	33	5	18.14282
186	84	06:00-20:30	126.659927	45.788479	126.540634	45.723045	48	5	28.18336
187	85	06:00-20:30	126.6279	45.760781	126.511963	45.723732	39	6	21.16758
188	86	05:50-20:30	126.725319	45.731827	126.579353	45.742676	29	6	18.98129
189	87		126.677879	45.797916	126.589272	45.706196	40	5	20.75028
190	89	05:30-21:00	126.609253	45.768448	126.709676	45.788399	31	5	17.75803
191	9		126.684654	45.705948	126.608215	45.775093	25	6	13.6483
192	90	06:00-20:30	126.586197	45.740692	126.708092	45.791622	33	6	20.56451
193	91		126.701637	45.741608	126.579559	45.742702	32	8	17.96714
194	92	06:00-21:00	126.661537	45.796677	126.581429	45.748684	25	6	14.81196

195	93	06:00-20:30	126.519447	45.722839	126.708069	45.79171	53	6	30.23535
196	94	05:30-21:00	126.625183	45.700035	126.665741	45.788204	32	2	16.91586
197	95		126.525161	45.722942	126.682373	45.798344	29	5	20.1887
198	96	06:00-21:00	126.631073	45.75729	126.582474	45.692417	17	6	8.82033
199	97	05:50-19:30	126.520035	45.719513	126.663399	45.787388	41	8	20.2025
200	98	05:30-18:00	126.556541	45.669594	126.648239	45.792862	38	10	24.10316
201	99	06:00-20:00	126.701904	45.743038	126.596184	45.731972	32	8	18.21614
