# Supplementary material for: Optimizing urban bus network based on spatial matching patterns for sustainable transportation: A case study in Harbin, China
Source: PLoS One. 2024 Oct 28;19(10):e0312803. doi: 10.1371/journal.pone.0312803 (PMC11515997; doi:10.1371/journal.pone.0312803)
Supplement: S2 Table — (PDF) [file pone.0312803.s002.pdf]

S2 Table. Bus station data

Number	Bus Station	Longitudes	Latitude
1	Cheliangchangwenhuagong	126.607918	45.771835
2	Jingweishidaojie	126.608139	45.770035
3	Jingweishidaojie	126.61055	45.768913
4	Daolishierdaojie	126.618607	45.770294
5	Zhaolinjie	126.626755	45.771473
6	Maimaijie	126.630745	45.772308
7	Nanmalu	126.639519	45.77441
8	Chengdeguangchang	126.639885	45.777508
9	Jingyangjie(temporary station)	126.636658	45.783222
10	Daowaisandaojie(temporary station)	126.637367	45.785568
11	Daowaiqidaojie(temporary station)	126.64193	45.787148
12	Daowaishierdaojie(temporary station)	126.649612	45.790199
13	Shisiyuan	126.656219	45.789715
14	Jingyuershidaolu	126.661423	45.791264
15	Songpudaqiao	126.661728	45.794079
16	Gengxinjie	126.668083	45.79604
17	Youshichang	126.673874	45.79784
18	Mucaishichang	126.67675	45.798737
19	Gangwuju	126.680664	45.799969
20	Dianjichang	126.659592	45.716816
21	Leyuanjie	126.655289	45.718266
22	Lesongguangchang	126.650604	45.720528
23	Forestry University	126.642616	45.723804
24	Linyezongyiyuan	126.63829	45.725292
25	Linxinglu	126.631271	45.727703
26	Harbin Normal University (South Campus)	126.625404	45.729725
27	Hexinglu	126.617462	45.732391
28	Tongdajie	126.619743	45.737541
29	Xidaqiao	126.626404	45.743317
30	Hagongda	126.633842	45.749725
31	Hatieluju	126.636169	45.751759
32	Shengbolanzhongxin	126.641228	45.756084
33	Qiulingongsi	126.649048	45.76281
34	Yidayiyuan	126.651833	45.765194
35	Yanchang	126.653336	45.768063
36	Hazhan	126.636871	45.757515
37	Shigushangyiyuan	126.622177	45.739899
38	Hesongjie	126.592392	45.748333
39	Hezhengxiaoqu	126.595001	45.752563
40	Hegujie	126.593452	45.754704
41	Heliangjie	126.595428	45.756866
42	Highway Bridge Station	126.594246	45.7612
43	Shehuikexueyuan	126.596016	45.764275
44	Baoyedasha	126.600044	45.768986
45	Shidishuiju	126.603142	45.772503
46	Shirenda	126.605934	45.774464
47	Fanghongjinianta(Youyilu)	126.619247	45.778694
48	Ertongyiyuan	126.623672	45.779171
49	Shilaoniandaxue	126.630882	45.783493
50	Daowaisandaojie	126.637512	45.785622
51	Daowaiqidaojie	126.642357	45.78727
52	Daowaishierdaojie	126.649757	45.790276
53	Daowaishiliudaojie	126.653244	45.791534
54	Daotaifu	126.659363	45.793362
55	Taocixiaoqu(temporary station)	126.683174	45.798584
56	Taocixiaoqu	126.685287	45.799206
57	Jingweishierdaojie	126.60331	45.770382
58	Gaoyijie	126.611679	45.773983
59	Dongfengjie	126.613075	45.77142
60	Jianzhuyishuguangchangzhan	126.626503	45.768719

61	Bowuguanzhan	126.640373	45.758423
62	Ertonggongyuan	126.661316	45.763454
63	Dachengjie	126.666321	45.760902
64	Xuanhuajie(Xianfenglu Junction)	126.670319	45.764454
65	Xianfengxiaoqu	126.673752	45.764961
66	Xianfenglu(Huashanlu Junction)	126.68026	45.765808
67	Songshanxiaoqu	126.684685	45.766285
68	Hongqidajie(Xianfenglu Junction)	126.689621	45.766918
69	Hongqidajie(Hongweilu Junction)	126.697723	45.767967
70	Shieryuan(temporary station)	126.699425	45.770065
71	Boyuexingcheng	126.703064	45.770935
72	Qianjinlu	126.596878	45.756271
73	Jianguojie	126.602966	45.752285
74	Anhong Street	126.609459	45.751862
75	Anshengjie	126.615562	45.759045
76	Beianjie	126.618828	45.763504
77	Jingweijie(temporary station)	126.620445	45.764439
78	Hazhanbeiguangchang(temporary station)	126.629639	45.76416
79	Bowuzhan	126.640579	45.758339
80	Hagongchengdaxue	126.66407	45.773373
81	Nantongdajie	126.670891	45.777836
82	Taipingqiao	126.680061	45.785213
83	Heilongjianggongchengxueyuan	126.683807	45.785099
84	Daxinxiecheng	126.686676	45.782326
85	Honghexiaoqu	126.689194	45.779209
86	Hongpingxiaoqu	126.690735	45.77449
87	Hongqijiajucheng	126.691788	45.770321
88	Hongweilu(Xianfenglu Junction)	126.697884	45.767986
89	Hepingxiaoqu	126.707451	45.764359
90	Xinhengguwancheng	126.708496	45.759781
91	Daolixierdaojie	126.620064	45.776672
92	Jingweixiao	126.614594	45.765911
93	Anfengjie	126.613068	45.763779
94	Anhejie	126.608925	45.757118
95	Jianguogongyuan	126.599915	45.747593
96	Shizhongyiyiyuan	126.59481	45.742088
97	Shengkangfuzhongxin	126.708359	45.744976
98	Nanzhilu(Hanshui Junction)	126.707695	45.742523
99	Jianchengchang	126.706818	45.739063
100	Shiyizhongxue	126.69944	45.735775
101	Jianbeilu	126.69529	45.736301
102	Zhujianglu	126.689095	45.737164
103	Shizhijianyuan	126.686371	45.73753
104	Shengfuerzhongxin	126.681747	45.737
105	Liushunjiesitujiekou	126.678825	45.73494
106	Zuguoyiyaoyanjiusuo	126.674042	45.733982
107	Xiangbinlu	126.669388	45.732681
108	Yamachang	126.661194	45.735432
109	Liangjuchang	126.657135	45.736782
110	Minshenglu	126.65255	45.738361
111	Wanjiachengshangchang	126.649635	45.739601
112	Wenjingjie	126.649773	45.741207
113	Fadianchang	126.650047	45.74461
114	Shigonggongdianchezonggongsi	126.647926	45.747406
115	Shengshiyanzhongxue(Hegoujie)	126.643654	45.750046
116	Manzhouliljie	126.635536	45.755272
117	Hayibai	126.624107	45.76825
118	Zhongguorenshoubaoxiangongsi	126.62265	45.773132
119	Daolisandaojie	126.62178	45.776039
120	Tongjiangshidigongjiaoshuniuzhan	126.611431	45.777867

121	Gongbin Road	126.70163	45.731895
122	Xiangbinxiaoxue	126.694618	45.730377
123	Hongqidajie(Gongbin Junction)	126.688866	45.729252
124	Hengdaojie	126.684448	45.730556
125	Caiyijie	126.68293	45.734161
126	Xiangfangwandaguangchang	126.677536	45.740891
127	Hanshuilu	126.678947	45.745682
128	shengrencaishicang	126.673233	45.748547
129	Zhongxuanjie	126.668137	45.745396
130	Xuanhuajie	126.667358	45.749969
131	Xuanhuajie(Lizhijie)	126.665916	45.753632
132	Gexinjie	126.660454	45.751606
133	Wenchangjie(temporary station)	126.657982	45.745808
134	Dongbeiyiyuan(temporary station)	126.654091	45.744411
135	Wenmingjie	126.650642	45.745583
136	Gongsijie	126.64254	45.749489
137	Fuhuaxiaoqu(Fanrongjie)	126.638428	45.745281
138	Fuhuaxiaoqu	126.635048	45.744663
139	Fushunjie	126.61747	45.754597
140	Anfaqiao	126.614441	45.755898
141	Andejie	126.610535	45.757465
142	anjianxinchenggongjiaoshoumozhan	126.608421	45.761875
143	Shengzhongyiyaodaxue	126.651413	45.72509
144	Hepingqiao	126.656853	45.732788
145	Shengwenlian	126.660515	45.739937
146	Wenfujie	126.657356	45.742443
147	Shengdanganguan	126.663185	45.749432
148	Jianshejie	126.643921	45.757015
149	Xianfenglu	126.705437	45.772739
150	Shengyaocaigongsi	126.704453	45.776653
151	Nanzhishangchang	126.703377	45.780964
152	Huashujie	126.701973	45.786713
153	Wangtongdongzhiluyingyeting	126.700478	45.792648
154	Jingyangjie	126.636856	45.782684
155	Kaiyuanerdaojie	126.637306	45.772678
156	Shengzhengfu	126.661766	45.744003
157	Wenchangjie	126.659859	45.745522
158	Gongrenwenhuagong	126.650856	45.752636
159	Hazhanbeiguangchang(Haichengqiao)	126.626785	45.760204
160	Tianyilanwangongjiaoshoumozhan	126.630531	45.779133
161	Dafashichang	126.604576	45.737286
162	Kanganlu	126.600037	45.741684
163	Zhongxinyiyuan	126.605896	45.748043
164	Anshengjie(Xinyanglu Junction)	126.61554	45.75901
165	Jingweijie	126.621277	45.766567
166	Ertongyiyuanzhan	126.623611	45.779163
167	Jiuzhangongjiaoshoumozhan	126.607201	45.7761
168	Fanghongjinaintazhan	126.61602	45.777988
169	Ertongdianyingyuan	126.623833	45.762947
170	Hazhanbeiguangchang(Jihongjie)	126.629341	45.764133
171	Swan Hote	126.66967	45.736938
172	Shengyiyuan	126.673271	45.730022
173	Xixiangfang	126.67598	45.726013
174	Hongqidajie(Gongbinlu Junction)	126.68914	45.728821
175	Shichuanranbingyiyuan	126.703094	45.732868
176	Jingweiliudaojie	126.614235	45.767281
177	Jingweisandaojie	126.617775	45.765644
178	Lieshiguan	126.644638	45.766312
179	Hongtujie	126.690063	45.780228
180	Yaoliujiayuan	126.695305	45.780918

181	Shiyinhang	126.625832	45.767433
182	Wangfujingfanyuecheng	126.630501	45.768425
183	Shibaqutiyuzhongxin	126.636757	45.769939
184	Shizhufanggongjijinzhongxin	126.640587	45.771294
185	Nanjishichang	126.644569	45.772533
186	Nanjijie	126.65242	45.774605
187	Binjiangzhan	126.655487	45.776367
188	Binjiangxinchenggongjiaoshoumozhan	126.66011	45.781124
189	Longjiangguoluchang	126.667236	45.795898
190	Daowaishibadaojie	126.658577	45.790665
191	Shidisiyiyuan	126.658005	45.790237
192	Dongbeiyiyuanzhan	126.654015	45.744438
193	Wenhuajie	126.651352	45.740074
194	Jianguojie(Kanganlu Junction)	126.592308	45.741589
195	Wujing Hospital	126.599243	45.7388
196	Sishiqizhongxue	126.612007	45.733971
197	Hazhanbeiguangchang(Haichengqiao)	126.626762	45.760227
198	Boligangyanjiusuo	126.691322	45.730751
199	Jianbeixiaoqu	126.693611	45.738316
200	Xiaofangyiyuan(temporary station)	126.694435	45.745132
201	Hanyayinhang	126.68927	45.75029
202	Yushanlu	126.686852	45.750088
203	Longta	126.678993	45.749481
204	Heilongjiangdongbeiyiyuan	126.654099	45.744411
205	Xiaohongzhongxue	126.63649	45.755302
206	Songhuajiangjie	126.632439	45.751766
207	Jiaohuaguangchang	126.628891	45.748783
208	Fushunxiaoxue	126.620003	45.756577
209	Shirencaishichang	126.621834	45.760185
210	Renliziyuanshichang	126.622917	45.762352
211	Hazhanbeiguangchang	126.627302	45.760218
212	Anfajie	126.610611	45.759918
213	Xinfaxiaoqu	126.662247	45.768242
214	Kuanchengjie	126.666016	45.765701
215	Xuanhuajie(Xianfeng Junction)	126.670296	45.764454
216	Xianfengxiaoqu(Huashanlu Junction)	126.680283	45.765812
217	Hongqidajie(Xianfeng Junction)	126.689636	45.766918
218	Hongweilu(Xianfeng Junction)	126.697929	45.76799
219	Nanzhilu	126.706284	45.768208
220	Jianzhuyishuguangchang	126.626495	45.76873
221	Wuyezhongxin	126.660561	45.778156
222	Harbingongyegaojijigongxuexiao	126.663635	45.780872
223	Dafanglixiaoqu	126.667175	45.784107
224	Xinglongbadaojie	126.671608	45.785709
225	Shilaonianyiyuan	126.675041	45.78521
226	Daowaigonganfenju	126.690193	45.788582
227	Huashujie(Hongwei Junction)	126.694832	45.78825
228	Changjianglu(Nanzhilu Junction)	126.710068	45.75153
229	Nanzhilu(Hanshuilu Junction)	126.707687	45.742508
230	Jianguojie(Kangan Junction)	126.592308	45.741585
231	Hazhan(Tielujie)	126.629723	45.757893
232	Yiererzhongxue	126.670326	45.720234
233	Diantajie	126.667221	45.715763
234	Shengbolanzhongxin(Lianfajie)	126.640121	45.752029
235	Sankongqiao	126.616631	45.740604
236	Hashangda(South School District)	126.610283	45.744072
237	Minanjie	126.612297	45.747978
238	Xinyanglu	126.606979	45.750534
239	Alaboguangchang	126.652557	45.787361
240	Nanxunshisidaojie	126.653275	45.784355

241	Nanxunjie	126.649002	45.782494
242	Taiguliudaojie	126.647316	45.780888
243	Taigutoudaojie	126.642197	45.779209
244	Wenjunhuayuanxiaoqu	126.639252	45.741879
245	Wenkujie	126.644646	45.738792
246	Wenzhengjie	126.64003	45.730274
247	Shengfunvganbuxueyuan	126.637878	45.72636
248	Wenchengjie	126.646637	45.741005
249	Xuandejie	126.664299	45.754826
250	Xinjishangcheng	126.66539	45.760536
251	Dingxinsandaojie	126.665199	45.765743
252	Dafangliweishengfuwuzhan	126.660553	45.778168
253	ershangdian	126.702713	45.792213
254	Taiguershidaojie	126.66404	45.786484
255	Gangtiejie	126.591522	45.74577
256	Fanghongjiniantagongjiaoshoumozhan	126.619568	45.778465
257	Zhongyangdajie	126.620995	45.774868
258	Hongxiajie	126.613297	45.773022
259	Jingweishiyidaojie	126.605019	45.767071
260	Aijianlu	126.603043	45.762661
261	Hezhoujie	126.598053	45.759872
262	Tielujie	126.625359	45.752197
263	Wajie	126.622017	45.746151
264	Xidazhijie	126.623482	45.743031
265	Hanguangjie(temporary station)	126.625244	45.735626
266	Wuruijie	126.629715	45.733364
267	Yikuangjie	126.632439	45.73679
268	Wangzhaoxincun	126.639313	45.733616
269	Tielueryuan	126.647217	45.73101
270	Wangzhaohuochezhan	126.649811	45.730148
271	Huaqiaomingyuanxiaoqu	126.656357	45.728024
272	Xiangbinluqiaokou	126.661823	45.726311
273	Shangyoujie(Zhongyangdajie)	126.615883	45.775814
274	Jianguogongyuan(Tongdajie)	126.599281	45.749088
275	Nanpingjie	126.644234	45.775688
276	Anshengjie(Xinyang Junction)	126.616379	45.760307
277	Steel Residential Area	126.594276	45.74839
278	Shiyiyuan	126.627281	45.773125
279	Zhaolingongyuan	126.624146	45.775734
280	Jingweijiudaojie	126.610809	45.770203
281	Anlongjie	126.607903	45.767494
282	Ansongjie	126.607758	45.764507
283	Yangmingjie	126.600922	45.757088
284	Aijianxinchengshoumozhan	126.608429	45.761898
285	Tongjiangjie	126.615242	45.77195
286	Shangyoujie	126.613884	45.774357
287	Hasanzhong	126.638824	45.764153
288	Guoxinzhengquangongsi	126.632515	45.76897
289	Youyigong	126.611547	45.777848
290	Gongchengjie	126.604134	45.771851
291	Chuangyezhongxin	126.689644	45.740086
292	Dadaojie	126.593903	45.754711
293	Yierwuzhong	126.654579	45.743195
294	Shengdianliyiyuan	126.700531	45.741413
295	Xiangjianglu	126.696274	45.74197
296	Heilongjiangshanglv(Hanshuilu)	126.683334	45.745224
297	Changjianglu	126.682762	45.750813
298	Huanghelu	126.677628	45.753609
299	Longyundasha(Lujiajie)	126.66803	45.756813
300	Zhuozhangouwuerqi	126.603645	45.760937

301	Shanghaijie	126.60186	45.763077
302	Jingyuqidaojie(Jingyudajie)	126.645172	45.786049
303	Taigushisidaojie	126.65464	45.784023
304	Yuhongxiaoxue	126.667801	45.769146
305	Benmaqipeicheng(Xianfenglu)	126.668037	45.766418
306	Shisanzhongxue	126.675194	45.76247
307	Hatiyuxueyuan	126.674934	45.759052
308	Haihelu(Xuanqingjie)	126.674713	45.756676
309	Yixuanguangtongzhaobiaojituan	126.691528	45.750111
310	Jiusanjituan	126.696198	45.748875
311	Minjiangxiaoqu	126.700188	45.747318
312	Taishanxiaoqu	126.70253	45.745338
313	Hongqixiaoqu	126.700691	45.762878
314	Huaihelu	126.696152	45.762356
315	Liaohexiaoqu	126.687065	45.761986
316	Zhongzhifangzhouyuan	126.682739	45.763271
317	Haihelu(Xuanqingjie Junction)	126.674706	45.756577
318	Shizijie	126.670265	45.755894
319	Shenggonganting	126.6661	45.744179
320	Wenjunhuayuan	126.640137	45.741745
321	Xianfenglu(Huashanbei Junction)	126.680267	45.765808
322	Liaohelu	126.693146	45.765404
323	Huaihelu(Hongqidajie Junction)	126.694069	45.761261
324	Huizhanzhongxin	126.696327	45.752289
325	Hanshuilu(Hongqidajie Junction)	126.695526	45.746563
326	Hongnanjie	126.696106	45.783237
327	Hongweilu	126.698059	45.775513
328	Shieryuan	126.699219	45.77087
329	Liaohelu(Hongqidajie Junction)	126.693146	45.765415
330	Huaihelu(Hongqidajiekou)	126.694099	45.761135
331	Xinyuanxiaoqu	126.694077	45.755379
332	Hagongdadierxiaoqu	126.68705	45.754505
333	Hongxianglu	126.673401	45.742767
334	Sandadonglilu	126.649651	45.723156
335	Zhaolingongyuanzhan	126.626007	45.776764
336	Shiyiyuanzhan	126.627563	45.77248
337	Xiashigushangkeyiyuanzhan	126.632576	45.766621
338	Hazhanbeiguangchanggongjiaoshoumozhan	126.627792	45.760479
339	Taihaihuayuanxiaoqu	126.706657	45.756927
340	Hongboshijiguangchang	126.697784	45.755863
341	Taishanlu	126.698105	45.742241
342	Huayuanjie	126.658539	45.767345
343	Yiererzhong	126.670311	45.720207
344	Liushunzongheshichang	126.67778	45.735115
345	Huashanlu(Ganshui Junction)	126.68055	45.741322
346	Chuangyezhongxin(temporary station)	126.689751	45.740067
347	Huaihelu(Hongqidajiejiekou)	126.694923	45.759655
348	Liaohelu(Hongqidajiejiekou)	126.693817	45.76339
349	Shiguoshuijichaju	126.684341	45.785019
350	Yimianjie(Senlinjie Junction)	126.629875	45.776924
351	Alaboguangchang(temporary station)	126.652657	45.787476
352	Nanxinershidaojie	126.663467	45.788742
353	Songshanlu	126.687593	45.763875
354	Guoluchanglinshizhan	126.66584	45.713593
355	Renliziyuanshichang(Anningjie)	126.620346	45.762512
356	Wangtongluyingyeting(Ershangdian)	126.700142	45.792614
357	Kunlunshangcheng(Hanshuilu)	126.673866	45.74752
358	Haguangdiantai	126.682121	45.740746
359	Hengdaojie(Gongbinlu)	126.684029	45.728168
360	Litchi Street	126.6679	45.754326

361	Xianfengxiaoqu(Huashan Junction)	126.680283	45.765812
362	Hashangdananxuequ	126.610107	45.74519
363	Fushunjiedidelijiekou(Fushunxiaoxue)	126.620003	45.756592
364	Beifangjuchang	126.639778	45.756271
365	Huashanluxianfenglulukou	126.680305	45.765812
366	Shengzhijianyuan	126.686409	45.737526
367	Xinjiangqiaoshichang	126.694733	45.791801
368	Taipingdajie	126.690598	45.790535
369	Taipingbeisandaojie	126.684975	45.788792
370	Taipingnansidaojie	126.681198	45.787682
371	Baqujie	126.637154	45.767601
372	Baqujienanjijiekou	126.638878	45.769562
373	Songpudaqiaonan	126.657867	45.792904
374	Nanzhixiaoqu	126.69487	45.797768
375	Wangtongdongzhiluyingyeting(Dongzhilu)	126.698776	45.791519
376	Yanpingjie	126.676346	45.782841
377	Dongdazhijie	126.654861	45.767105
378	Longyundasha	126.667717	45.756569
379	Hashidananxuequ	126.625481	45.729702
380	Zhongbeichuncheng	126.668365	45.728535
381	Zhongxin Department Store	126.689301	45.728859
382	Xinjiangqiaojie	126.694664	45.791782
383	Jiashujie	126.63269	45.75415
384	Hazhantielujie	126.62999	45.757511
385	Laoshichang	126.694687	45.730404
386	Linshizhan	126.592804	45.746117
387	Herunjie	126.599442	45.755141
388	Daowaiershidaojie	126.663857	45.787651
389	Daowaikeyunzhan	126.642288	45.777462
390	Tongdajie(Jianguobeierdaojie)	126.599342	45.749062
391	Minzhongjie	126.60891	45.743832
392	Xuanqingxiaoqu	126.680817	45.753773
393	Shengshizihui	126.669975	45.752991
394	Wenchangjiezhan	126.65789	45.745754
395	Hashida(Nanxiaoqu)	126.624588	45.730003
396	Taiping Second Shop	126.701096	45.791996
397	Beijingjie(Manzhoulijiekou)	126.638	45.755959
398	Hongqidajiegongbinlukou	126.689308	45.728859
399	Taipingsandaojie	126.684959	45.788792
400	Taipingsidaojie	126.681145	45.78767
401	Tongjiangshidigongjiaohuanchengshuniuzhan	126.611512	45.777599
402	Yimianjie	126.629387	45.777325
403	Taigushierdaojie	126.653633	45.782215
404	Taigushiliudaojie	126.659645	45.783852
405	Xianfenglu(Huashan Junction)	126.679176	45.764378
406	Harbintiyuxueyuan	126.679893	45.761505
407	Lvseshipinjiaoyizhongxin	126.70491	45.751488
408	Hanguangjie	126.625366	45.73555
409	Qizhengjie	126.624161	45.735085
410	Boyuexingchenggongjiaoshoumozhan	126.70285	45.770912
411	Zhaolinjie	126.626099	45.771332
412	Hongqixiaoqu(temporary station)	126.701103	45.762928
413	Sandadonglifulu	126.659668	45.715672
414	Hanshuilu(Hongqidajiekou)	126.695122	45.74353
415	Kunlunxiaoqu	126.704681	45.774906
416	Weixinglu	126.7024	45.770859
417	Wenhuadasha(temporary station)	126.629677	45.733383
418	Tiandijie	126.632957	45.770943
419	Yimianjie(temporary station)	126.629448	45.777508
420	Youyigong(temporary station)	126.608826	45.776039

421	Xixiangfang(Gongbinlu)	126.677185	45.726772
422	Huizhanzhongxintiyuchang	126.707367	45.752216
423	Hazhan(Haichengqiao)	126.63002	45.757816
424	Nanxunbadaojie	126.650818	45.783607
425	Jingyujie	126.651695	45.789021
426	Anfengshangchang	126.612816	45.763229
427	Jingweijie(Zhongyangdajie)	126.621597	45.766685
428	Heilongjianggongchengdaxue	126.683792	45.785114
429	Gexinjiezhan	126.660454	45.751606
430	Shengdanganshi	126.662224	45.750217
431	Shizijie(Dachengjiekou)	126.671043	45.759178
432	Maduanjie	126.672081	45.763325
433	Hatiyuxueyuandongmen	126.679947	45.761307
434	Jiangongxinqu	126.680801	45.757435
435	Hazhan(Haichengqiao)(temporary station)	126.630096	45.757771
436	Nanxinshisidaojie	126.655067	45.784794
437	Huangjiahuayuan	126.665741	45.788208
438	Gongludaqiao(Heluojie)	126.592812	45.759911
439	Nanjijie(Chengdejie)	126.647972	45.77512
440	Hongzhuanjie	126.611473	45.771461
441	Gongchengjie(temporary station)	126.607193	45.771698
442	Xiangjianglu(Hongqidajie Junction)	126.69577	45.742043
443	Xiangshunjie	126.676414	45.729179
444	Xiaofangyiyuan	126.694389	45.745136
445	Hasanzhong(temporary station)	126.63887	45.764126
446	Heilongjiangkejixueyuan(Songshanxiaoqu)	126.687904	45.758537
447	Benmaqipeicheng	126.668312	45.768055
448	Dongbeiyiyuan	126.654091	45.744411
449	Fanrongjie	126.638504	45.745502
450	Taigushisidaojie(temporary station)	126.654945	45.783848
451	Hongqidajie(Beihuanlu Junction)	126.677917	45.797787
452	Jingyudajie	126.655182	45.789627
453	Shengyiyuannangnagfenyuan	126.643318	45.763924
454	Quxianjie	126.624619	45.751316
455	Tieludiyixiaoxue	126.622017	45.746174
456	Wenhuadasha	126.629707	45.733368
457	Kunlunshangcheng	126.673668	45.74556
458	Hequjie	126.59272	45.760357
459	Changjianglunanzhilukou	126.710083	45.751541
460	Nanzhiluhanshuilukou	126.707687	45.742508
461	Liushunjie(Situjie Junction)	126.678833	45.73494
462	Tongdajie(Jianguogongyuan)	126.599503	45.748997
463	Anshunjie	126.611443	45.766659
464	Minjianglu(Nanzhilu Junction)	126.70842	45.746246
465	Xidazhijie(temporary station)	126.621437	45.737881
466	Shengzhengxie	126.669594	45.737015
467	Tianmuxiaoqu	126.705986	45.763435
468	Renlijie	126.659927	45.788479
469	'angtongdongzhiluyingyeting(temporary statio	126.700104	45.792721
470	Yaocaigongsi	126.704483	45.775681
471	Huaihelu(temporary station)	126.693214	45.761978
472	Qianjinlu(Hesongjiekou)	126.593025	45.747715
473	Xiashigushangyiyuan	126.634407	45.766521
474	Xiangdianjie	126.693321	45.733955
475	Huashanlu(Changjianglu Junction)	126.68277	45.750748
476	Shengbolanzhongxinzhan	126.64019	45.755325
477	Hongqidajie(Beihuanlukou)	126.677879	45.797916
478	Lujiajie	126.658905	45.758205
479	Renhejie	126.656425	45.755974
480	Huayuanshangdian	126.653709	45.763123

481	Liaoyangjie	126.656197	45.765255
482	Ankangjie	126.609261	45.766682
483	Jingweijie(Gaoyijie)	126.612137	45.769913
484	Jianguojie(Kanganlulukou)	126.5923	45.741585
485	Yaoliujiayuan(temporary station)	126.695358	45.78093
486	Shierzhongxue	126.695152	45.786968
487	Lujiajie(Shizijie Junction)	126.670746	45.756252
488	Renhejie(Jianxinjiekou)	126.661438	45.760384
489	Harbin Print Center	126.589966	45.742512
490	Miaopujie	126.630585	45.733593
491	Shierzhong	126.695152	45.786968
492	Bowuguanzhan(temporary station)	126.641212	45.757027
493	Hongqidajie	126.67878	45.797264
494	Tielujie(Shangfangjie)	126.627884	45.755138
495	Tielujie(temporary station)	126.62545	45.752308
496	Tielujieqiaobeijiekou	126.617699	45.743694
497	Tielujieqingmingerdaokou	126.613777	45.739738
498	Binjianghuozhan	126.657501	45.780022
499	Huochezhan	126.63282	45.758968
