# Supplementary material for: Optimizing urban bus network based on spatial matching patterns for sustainable transportation: A case study in Harbin, China
Source: PLoS One. 2024 Oct 28;19(10):e0312803. doi: 10.1371/journal.pone.0312803 (PMC11515997; doi:10.1371/journal.pone.0312803)
Supplement: S3 Table — (PDF) [file pone.0312803.s003.pdf]

S3 Table. Population density data

Number	Bus Station	6:30	6:40	6:50	7:00	7:10	7:20	7:30	7:40	7:50	8:00	8:10	8:20	8:30	8:40	8:50	9:00	9:10	9:20	9:30
1	Cheliangchangwenhuagong	1	1	1	1	1	1	1	2	2	2	2	2	2	2	2	2	2	2	2
2	Jingweishidaojie	1	1	1	1	1	1	1	2	2	2	2	2	2	2	2	2	2	2	2
3	Jingweishidaojie	1	1	2	2	1	1	1	3	3	3	3	3	3	3	3	3	3	3	3
4	Daolishierdaojie	1	1	1	1	1	1	1	1	1	2	3	3	3	3	3	2	3	3	3
5	Zhaolinjie	1	1	1	2	2	2	2	2	2	2	3	3	3	5	3	3	3	3	3
6	Maimaijie	1	1	1	1	1	2	2	2	2	2	2	2	2	2	2	2	2	2	2
7	Nanmalu	1	2	1	2	2	2	2	2	2	2	3	3	3	3	3	3	3	3	3
8	Chengdeguangchang	1	1	1	1	1	1	1	1	1	2	2	2	2	2	2	2	2	2	2
9	Jingyangjie(temporary station)	1	1	1	1	1	1	1	1	1	1	1	1	1	2	2	2	2	2	2
10	Daowaisandaojie(temporary station)	1	1	1	1	1	1	1	1	1	1	1	1	1	1	1	2	2	2	2
11	Daowaiqidaojie(temporary station)	1	1	1	1	1	1	2	2	2	1	2	2	2	3	3	3	3	3	3
12	Daowaishierdaojie(temporary station)	1	1	2	2	2	2	2	3	3	4	4	4	4	4	4	4	4	4	4
13	Shisiyuan	2	2	2	2	2	2	2	2	2	3	3	3	3	3	3	3	3	3	3
14	Jingyuershidaolu	1	1	2	2	2	2	2	2	2	2	3	3	3	3	3	3	3	3	3
15	Songpudaqiao	1	1	1	1	1	1	1	2	2	2	3	3	3	3	3	3	3	3	3
16	Gengxinjie	1	1	1	1	1	1	1	1	1	1	1	1	1	1	1	1	1	1	1
17	Youshichang	1	1	1	1	1	1	1	1	1	1	1	1	1	1	1	1	1	1	1
18	Mucaishichang	1	1	1	1	1	1	2	2	2	1	2	1	2	2	2	2	2	2	2
19	Gangwuju	1	1	1	1	1	1	2	2	2	1	2	1	2	1	2	2	2	2	1
20	Dianjichang	1	1	1	1	1	1	1	1	1	1	1	1	1	2	1	1	1	1	1
21	Leyuanjie	1	1	1	1	1	1	1	1	1	1	1	1	1	2	2	2	2	1	1
22	Lesongguangchang	1	1	1	1	1	1	1	2	2	2	2	1	2	2	2	2	2	2	2
23	Forestry University	1	1	1	1	1	1	1	2	2	3	3	3	3	3	3	3	3	3	3
24	Linyezongyiyuan	1	1	2	2	2	2	2	3	3	4	5	4	5	5	5	5	5	5	5
25	Linxinglu	1	1	1	1	1	2	1	1	1	2	2	2	2	2	2	2	2	2	2
26	Harbin Normal University (South Campus)	1	1	2	2	2	2	2	3	3	3	4	3	5	5	5	5	5	4	4
27	Hexinglu	1	1	2	3	3	3	3	5	5	5	5	3	5	5	5	5	5	5	5
28	Tongdajie	1	1	2	2	2	2	2	4	4	5	5	4	5	5	5	5	5	5	5
29	Xidaqiao	1	1	1	1	1	1	1	2	2	3	3	3	4	4	4	4	4	5	5
30	Hagongda	1	1	1	1	1	1	1	1	1	2	3	3	3	3	3	3	3	3	3
31	Hatieluju	1	1	1	1	1	1	1	1	1	2	2	2	2	2	2	2	2	2	2
32	Shengbolanzhongxin	1	1	1	1	1	1	1	2	2	3	4	4	4	4	4	4	4	4	4
33	Qiulingongsi	1	1	2	3	3	3	3	4	4	4	5	5	6	6	6	6	6	6	6
34	Yidayiyuan	1	1	3	3	2	2	2	3	3	3	5	4	5	5	5	5	5	5	5
35	Yanchang	1	1	1	1	1	1	1	2	2	2	3	3	3	3	3	3	3	3	3
36	Hazhan	1	1	1	1	1	1	1	3	3	3	3	3	3	3	3	3	3	3	3
37	Shigushangyiyuan	1	1	2	2	2	2	2	3	3	4	4	3	5	5	5	5	5	5	6
38	Hesongjie	1	1	2	2	2	2	3	3	3	4	4	3	4	4	4	5	4	4	4
39	Hezhengxiaoqu	1	1	2	2	2	2	3	3	3	3	4	3	5	5	5	5	5	5	5
40	Hegujie	2	2	2	2	2	2	3	3	3	4	4	3	4	4	4	4	4	5	5
41	Heliangjie	2	2	2	2	3	2	4	3	3	5	5	4	5	5	5	5	5	5	5
42	Highway Bridge Station	1	1	1	1	1	2	2	3	3	2	4	4	4	4	4	4	4	5	5
43	Shehuikexueyuan	1	1	1	1	1	1	1	1	1	2	2	2	3	3	3	3	3	5	5
44	Baoyedasha	1	1	1	1	1	1	1	1	1	2	2	2	3	3	3	3	3	3	3
45	Shidishuiju	1	1	1	1	1	1	1	1	1	1	2	2	2	2	2	2	2	2	2
46	Shirenda	1	1	1	1	1	1	1	1	2	1	2	2	2	1	1	1	1	1	1
47	Fanghongjinianta(Youyilu)	1	1	1	1	1	1	1	1	1	1	2	1	2	2	2	2	2	2	2
48	Ertongyiyuan	1	1	1	1	1	1	2	1	1	2	2	2	2	2	2	2	2	2	2
49	Shilaoniandaxue	1	1	1	1	1	1	1	1	1	1	1	1	1	2	2	2	2	2	2
50	Daowaisandaojie	1	1	1	1	1	1	1	1	1	1	1	1	1	1	1	2	2	2	2
51	Daowaiqidaojie	1	1	1	1	1	1	1	2	2	2	3	3	3	3	3	3	3	3	3
52	Daowaishierdaojie	1	1	2	2	2	2	2	3	3	4	4	4	4	4	4	4	4	4	4
53	Daowaishiliudaojie	1	1	2	2	2	2	2	2	3	3	3	3	3	3	3	3	3	3	3
54	Daotaifu	1	1	2	2	2	2	2	2	2	3	3	3	3	3	3	3	3	3	3
55	Taocixiaoqu(temporary station)	1	1	1	1	2	1	2	3	3	2	2	2	2	2	2	2	2	2	2
56	Taocixiaoqu	1	1	1	1	1	1	1	2	2	1	2	1	1	1	2	2	2	2	2
57	Jingweishierdaojie	1	1	1	1	1	1	1	2	2	2	2	2	3	3	3	3	3	3	3
58	Gaoyijie	1	1	1	1	1	1	2	2	2	2	3	3	3	2	2	2	2	2	2
59	Dongfengjie	1	1	1	1	1	1	1	2	2	3	3	3	3	3	3	3	3	3	3

60	Jianzhuyishuguangchangzhan	1	1	1	1	1	1	1	1	1	2	2	2	2	2	2	2	2	2	2
61	Bowuguanzhan	1	1	1	1	1	1	1	2	2	3	4	4	4	4	4	4	4	4	4
62	Ertonggongyuan	1	1	1	1	2	2	2	3	3	3	3	3	3	3	3	3	3	3	3
63	Dachengjie	1	1	2	2	2	2	2	3	3	3	4	4	4	4	4	4	4	4	4
64	Xuanhuajie(Xianfenglu Junction)	1	1	2	2	2	2	2	3	3	3	3	3	3	3	3	3	3	3	3
65	Xianfengxiaoqu	1	1	1	1	2	2	2	3	3	3	2	2	2	3	2	2	2	2	2
66	Xianfenglu(Huashanlu Junction)	1	1	1	1	1	1	2	2	2	2	2	1	2	2	2	2	2	2	2
67	Songshanxiaoqu	1	1	1	1	1	1	1	2	2	2	2	2	2	2	2	2	2	2	2
68	Hongqidajie(Xianfenglu Junction)	1	1	1	1	1	1	1	2	2	2	2	2	3	3	2	2	3	2	2
69	Hongqidajie(Hongweilu Junction)	1	1	1	1	1	1	2	2	2	2	2	2	3	2	2	2	2	2	2
70	Shieryuan(temporary station)	1	1	1	1	1	1	1	2	2	2	2	1	2	2	2	2	2	2	2
71	Boyuexingcheng	1	1	1	1	2	2	2	2	2	2	2	1	2	2	2	2	2	2	2
72	Qianjinlu	2	2	2	2	2	2	3	3	3	4	5	5	5	5	5	5	5	5	5
73	Jianguojie	1	2	2	2	2	2	4	3	3	4	4	4	4	5	4	5	5	5	5
74	Anhong Street	1	1	2	2	2	2	2	3	3	3	3	3	3	3	3	3	3	3	3
75	Anshengjie	2	2	2	2	2	2	2	4	4	4	5	5	5	5	5	5	5	5	5
76	Beianjie	2	2	2	3	3	3	3	4	4	5	5	5	5	5	5	5	5	5	5
77	Jingweijie(temporary station)	1	1	2	3	3	3	3	4	4	5	5	5	5	5	5	5	5	5	5
78	Hazhanbeiguangchang(temporary station)	1	1	2	2	2	2	2	3	3	3	4	3	3	3	3	4	4	4	4
79	Bowuzhan	1	1	1	1	1	1	1	2	2	3	4	3	4	4	4	4	4	4	4
80	Hagongchengdaxue	1	1	2	2	2	2	2	3	3	4	4	3	4	4	4	4	4	4	4
81	Nantongdajie	1	1	1	1	1	1	1	1	1	1	1	1	1	1	1	1	1	1	1
82	Taipingqiao	1	1	1	1	1	1	2	2	2	3	3	3	4	4	4	4	4	4	4
83	Heilongjianggongchengxueyuan	1	1	1	1	1	1	2	2	2	3	2	2	2	3	3	3	3	3	3
84	Daxinxiecheng	1	1	1	1	1	1	2	2	2	2	2	2	2	3	3	3	3	3	3
85	Honghexiaoqu	1	1	1	1	1	2	2	2	2	2	2	2	2	2	3	3	3	3	3
86	Hongpingxiaoqu	1	1	2	2	2	2	2	2	2	3	2	2	2	2	3	3	3	3	3
87	Hongqijiajucheng	1	1	1	1	1	1	1	1	1	1	2	1	2	2	2	2	2	2	2
88	Hongweilu(Xianfenglu Junction)	1	1	1	1	1	1	2	2	2	2	2	2	2	2	2	2	2	2	2
89	Hepingxiaoqu	1	1	1	1	2	2	2	2	2	2	3	3	3	3	3	4	4	4	4
90	Xinhengguwancheng	1	1	2	2	2	2	2	3	3	3	4	3	4	4	4	4	4	4	4
91	Daolixierdaojie	1	1	1	1	1	1	1	1	1	1	2	3	2	3	3	3	3	3	3
92	Jingweixiao	2	2	3	3	3	3	3	4	4	5	5	5	5	5	5	5	5	5	5
93	Anfengjie	2	2	2	3	3	3	3	3	4	4	4	4	4	4	4	4	4	4	4
94	Anhejie	2	1	2	2	2	2	2	4	4	4	5	5	5	5	5	5	5	5	5
95	Jianguogongyuan	1	2	2	2	2	3	4	4	4	5	5	5	6	5	5	5	5	5	5
96	Shizhongyiyiyuan	2	2	2	3	3	3	4	4	4	5	5	5	5	5	5	5	5	5	5
97	Shengkangfuzhongxin	1	1	1	1	1	1	1	2	2	2	2	2	3	3	4	4	4	4	4
98	Nanzhilu(Hanshui Junction)	1	1	1	1	1	1	2	2	2	2	2	2	3	3	3	3	3	3	3
99	Jianchengchang	1	1	1	1	1	2	2	2	2	2	3	3	3	3	2	2	3	3	3
100	Shiyizhongxue	1	1	2	1	1	2	2	2	2	2	3	3	3	2	2	2	3	3	3
101	Jianbeilu	1	1	1	1	1	2	2	2	2	2	2	2	3	2	3	3	3	3	2
102	Zhujianglu	1	1	1	1	1	1	1	2	2	2	4	3	4	4	5	5	5	5	5
103	Shizhijianyuan	1	1	1	1	1	1	1	1	2	1	4	3	4	5	5	5	5	5	5
104	Shengfuerzhongxin	1	1	1	1	1	1	1	1	1	1	3	3	2	3	3	3	3	3	3
105	Liushunjiesitujiekou	1	1	1	1	1	1	2	2	2	2	2	2	2	2	3	3	3	4	4
106	Zuguoyiyaoyanjiusuo	1	1	1	1	1	1	1	1	1	2	2	2	2	2	2	2	2	2	2
107	Xiangbinlu	1	1	1	1	1	1	1	2	2	2	3	3	3	3	3	3	3	3	3
108	Yamachang	1	1	2	2	2	2	2	3	3	3	4	4	4	4	4	4	4	4	4
109	Liangjuchang	1	1	2	2	2	2	2	3	3	3	3	3	3	3	3	3	3	3	3
110	Minshenglu	1	1	2	2	2	2	2	3	3	2	3	3	3	3	3	3	3	3	3
111	Wanjiachengshangchang	1	1	2	1	1	1	1	3	3	2	4	3	4	4	4	4	4	4	4
112	Wenjingjie	1	1	1	1	1	1	1	2	2	2	3	3	3	3	3	3	3	3	3
113	Fadianchang	1	1	2	2	2	2	2	2	3	3	3	3	3	3	3	3	3	3	3
114	Shigonggongdianchezonggongsi	1	1	1	1	1	1	1	2	2	2	2	2	2	2	2	2	2	2	2
115	Shengshiyanzhongxue(Hegoujie)	1	1	1	1	1	1	1	1	1	1	1	1	1	1	1	1	1	1	1
116	Manzhouliljie	1	1	1	1	1	1	1	2	2	3	3	3	3	3	3	3	3	3	3
117	Hayibai	1	1	1	1	1	1	1	2	2	2	3	2	2	3	2	2	3	2	3
118	Zhongguorenshoubaoxiangongsi	1	1	1	1	1	2	2	1	1	2	3	3	3	5	5	5	5	5	5
119	Daolisandaojie	1	1	1	1	1	1	1	1	1	2	3	3	2	3	3	3	3	3	3

120	Tongjiangshidigongjiaoshuniuzhan	1	1	1	1	1	1	1	1	1	1	2	1	1	1	1	1	1	1	1
121	Gongbin Road	1	1	2	2	2	2	3	3	3	3	3	3	5	3	3	3	3	3	4
122	Xiangbinxiaoxue	1	1	2	2	2	2	2	3	3	3	3	3	4	3	5	5	4	4	4
123	Hongqidajie(Gongbin Junction)	1	2	2	2	2	3	3	4	4	4	4	3	5	5	5	5	5	5	5
124	Hengdaojie	1	2	2	2	2	3	3	4	4	4	4	4	5	4	4	4	4	4	4
125	Caiyijie	1	1	1	1	1	1	2	3	3	3	3	3	3	3	3	3	3	4	4
126	Xiangfangwandaguangchang	1	1	1	1	1	1	1	1	1	1	1	1	1	2	2	2	2	2	2
127	Hanshuilu	1	1	1	1	1	1	1	1	1	1	2	1	2	3	3	3	3	3	3
128	shengrencaishicang	1	1	1	1	1	1	1	1	1	1	2	2	2	2	2	2	2	2	2
129	Zhongxuanjie	1	1	1	1	1	1	1	2	2	2	2	2	2	2	2	2	2	2	2
130	Xuanhuajie	1	1	1	1	2	2	2	3	3	3	3	3	3	3	3	3	3	3	3
131	Xuanhuajie(Lizhijie)	2	2	2	2	3	3	3	3	3	3	5	5	5	5	5	5	5	5	5
132	Gexinjie	1	1	2	2	2	2	2	3	3	3	3	3	3	3	3	3	3	3	3
133	Wenchangjie(temporary station)	1	1	2	2	2	2	2	2	2	4	5	5	5	5	5	5	5	5	5
134	Dongbeiyiyuan(temporary station)	1	1	2	2	2	2	2	2	2	3	4	3	4	4	4	4	4	4	4
135	Wenmingjie	1	1	2	2	2	2	2	3	3	3	3	3	3	3	3	3	3	3	3
136	Gongsijie	1	1	1	1	1	1	1	1	1	1	1	1	1	1	1	1	1	1	1
137	Fuhuaxiaoqu(Fanrongjie)	1	1	1	1	1	1	1	1	1	2	2	2	3	3	3	3	3	2	2
138	Fuhuaxiaoqu	1	1	1	1	1	1	1	1	1	2	2	2	3	3	3	2	3	3	3
139	Fushunjie	1	1	2	3	3	3	3	3	3	4	4	4	5	4	4	4	4	4	4
140	Anfaqiao	2	2	3	2	2	2	2	4	4	5	5	5	5	5	5	5	5	5	5
141	Andejie	2	2	2	2	2	2	2	5	4	4	5	5	5	5	5	5	5	5	5
142	anjianxinchenggongjiaoshoumozhan	1	1	2	2	2	2	2	2	2	2	2	2	2	2	2	2	2	2	2
143	Shengzhongyiyaodaxue	1	1	1	1	1	1	1	2	2	2	2	2	2	2	2	2	2	2	2
144	Hepingqiao	1	1	2	2	2	2	2	2	2	3	3	3	3	3	3	3	3	3	3
145	Shengwenlian	1	1	1	1	1	1	2	2	2	3	3	3	3	3	3	3	3	3	3
146	Wenfujie	1	1	1	1	1	1	1	2	2	3	4	3	3	3	3	3	3	3	3
147	Shengdanganguan	1	1	2	2	2	2	2	3	3	3	3	3	3	3	3	3	3	3	3
148	Jianshejie	1	1	1	1	1	1	1	2	2	2	5	5	5	5	5	5	5	5	5
149	Xianfenglu	1	1	1	1	1	2	2	2	2	2	2	1	2	2	2	2	2	2	2
150	Shengyaocaigongsi	1	1	1	1	1	1	1	1	2	2	1	1	2	2	2	2	2	1	1
151	Nanzhishangchang	1	1	1	1	1	2	2	2	2	2	2	2	2	3	2	2	2	2	2
152	Huashujie	1	1	2	2	2	2	2	2	2	2	3	3	3	3	3	3	3	2	2
153	Wangtongdongzhiluyingyeting	1	1	1	2	2	1	2	2	3	3	2	2	3	2	2	2	2	2	2
154	Jingyangjie	1	1	1	1	1	1	1	1	1	1	1	1	1	2	2	2	2	2	2
155	Kaiyuanerdaojie	1	1	1	2	2	2	2	2	2	2	2	2	2	3	3	3	3	3	3
156	Shengzhengfu	1	1	1	1	1	1	1	2	2	3	3	3	3	3	3	3	3	3	3
157	Wenchangjie	1	1	2	2	2	2	2	3	3	3	5	5	5	5	5	5	5	5	5
158	Gongrenwenhuagong	1	1	1	1	1	1	1	2	2	2	2	2	2	2	3	2	2	2	2
159	Hazhanbeiguangchang(Haichengqiao)	1	1	1	2	2	2	2	2	2	2	2	2	2	2	2	2	2	2	2
160	Tianyilanwangongjiaoshoumozhan	1	1	2	1	1	2	2	2	2	3	2	2	2	3	3	3	3	3	3
161	Dafashichang	1	1	1	1	1	1	1	2	2	3	3	3	3	3	3	3	3	3	3
162	Kanganlu	1	2	2	3	3	3	3	4	4	4	5	5	4	5	4	4	4	4	5
163	Zhongxinyiyuan	1	1	3	2	2	2	2	3	3	4	5	5	5	5	5	5	5	5	5
164	Anshengjie(Xinyanglu Junction)	2	2	2	2	2	2	2	4	4	4	5	5	5	5	5	5	5	5	5
165	Jingweijie	1	1	2	2	2	2	2	3	3	4	4	3	4	4	4	4	4	4	4
166	Ertongyiyuanzhan	1	1	1	1	1	1	2	1	1	2	2	2	2	2	2	2	2	2	2
167	Jiuzhangongjiaoshoumozhan	1	1	1	1	1	1	1	1	1	1	2	1	1	1	1	1	1	1	1
168	Fanghongjinaintazhan	1	1	1	1	1	1	1	1	1	1	2	1	2	2	2	2	2	2	2
169	Ertongdianyingyuan	1	1	2	2	2	2	2	3	3	3	3	3	3	3	3	3	3	3	3
170	Hazhanbeiguangchang(Jihongjie)	1	1	2	2	2	2	2	3	3	3	3	3	3	3	3	4	4	3	3
171	Swan Hote	1	1	1	1	1	1	1	1	1	2	2	2	2	2	2	2	2	2	2
172	Shengyiyuan	1	1	2	2	2	2	2	3	3	4	4	4	4	4	4	4	4	4	3
173	Xixiangfang	1	1	2	2	2	2	2	3	3	3	3	3	3	3	3	3	3	3	3
174	Hongqidajie(Gongbinlu Junction)	1	2	2	2	2	3	3	4	4	4	4	3	5	5	5	5	5	5	5
175	Shichuanranbingyiyuan	1	2	2	2	2	2	3	3	3	3	4	3	5	4	4	4	4	4	4
176	Jingweiliudaojie	2	2	2	2	2	2	2	3	3	5	5	4	4	4	4	4	5	5	5
177	Jingweisandaojie	2	2	2	3	3	3	3	4	4	5	5	5	5	5	5	5	5	5	5
178	Lieshiguan	1	1	2	2	2	2	2	2	3	2	4	4	4	4	4	4	4	4	4
179	Hongtujie	1	1	1	1	1	2	2	3	2	2	2	2	2	3	3	3	3	3	3

180	Yaoliujiayuan	1	1	2	2	2	2	2	3	3	3	2	2	2	4	3	3	3	3	3
181	Shiyinhang	1	1	1	1	1	1	1	2	2	3	3	2	3	3	3	2	3	2	3
182	Wangfujingfanyuecheng	1	1	1	1	1	1	1	2	2	2	1	1	1	1	1	1	1	1	1
183	Shibaqutiyuzhongxin	1	1	1	1	2	2	2	2	2	2	2	2	2	2	2	2	2	2	2
184	Shizhufanggongjijinzhongxin	1	1	1	2	2	2	2	3	3	3	4	3	4	4	3	4	4	3	3
185	Nanjishichang	1	1	2	2	2	2	2	3	3	5	5	5	5	5	5	5	5	5	5
186	Nanjijie	1	1	1	1	1	1	1	2	2	3	2	2	2	2	2	2	2	2	2
187	Binjiangzhan	1	1	1	1	1	1	1	2	1	2	2	2	2	2	2	2	2	2	2
188	Binjiangxinchenggongjiaoshoumozhan	1	1	2	2	2	2	2	3	3	4	3	3	3	3	3	3	3	3	3
189	Longjiangguoluchang	1	1	1	1	1	1	1	1	1	1	1	1	1	1	1	1	1	1	1
190	Daowaishibadaojie	1	1	2	2	2	2	2	2	2	2	3	3	3	3	3	3	3	3	3
191	Shidisiyiyuan	1	1	2	2	2	2	2	2	2	3	3	3	3	3	3	3	3	3	3
192	Dongbeiyiyuanzhan	1	1	2	2	2	2	2	2	2	3	4	3	4	4	4	4	4	4	4
193	Wenhuajie	1	1	1	1	1	1	1	3	3	2	3	3	3	3	3	3	3	3	3
194	Jianguojie(Kanganlu Junction)	2	2	3	3	3	4	5	5	5	5	6	5	6	6	6	6	6	6	6
195	Wujing Hospital	2	2	2	3	3	3	4	4	4	5	5	6	6	6	6	6	6	5	5
196	Sishiqizhongxue	1	1	1	2	2	2	2	3	3	4	4	4	5	5	5	5	5	5	5
197	Hazhanbeiguangchang(Haichengqiao)	1	1	1	2	2	2	2	2	2	2	2	2	2	2	2	2	2	2	2
198	Boligangyanjiusuo	1	2	2	2	2	2	3	3	3	3	4	4	4	4	5	5	5	4	4
199	Jianbeixiaoqu	1	1	1	1	1	2	2	2	2	2	2	3	3	2	5	5	5	4	4
200	Xiaofangyiyuan(temporary station)	1	1	1	1	1	1	1	1	1	2	2	1	1	3	2	2	2	3	3
201	Hanyayinhang	1	1	1	1	1	1	1	1	1	1	2	2	2	2	2	2	2	2	2
202	Yushanlu	1	1	1	1	1	1	1	1	1	1	2	1	2	2	2	2	2	2	2
203	Longta	1	1	1	1	1	1	1	1	1	1	2	2	2	2	2	2	2	2	2
204	Heilongjiangdongbeiyiyuan	1	1	2	2	2	2	2	2	2	3	4	3	4	4	4	4	4	4	4
205	Xiaohongzhongxue	1	1	1	1	1	1	1	2	2	3	3	3	3	3	3	3	3	3	3
206	Songhuajiangjie	1	1	1	1	1	1	1	1	1	2	3	3	3	3	3	3	3	3	3
207	Jiaohuaguangchang	1	1	1	1	1	1	1	2	2	3	4	3	4	4	4	4	4	4	4
208	Fushunxiaoxue	1	1	2	2	2	2	2	3	3	4	4	3	4	4	4	4	4	4	4
209	Shirencaishichang	1	1	1	2	2	2	2	2	2	3	3	3	3	3	3	3	3	3	3
210	Renliziyuanshichang	1	1	1	2	2	2	2	3	2	3	3	3	3	3	3	3	3	3	3
211	Hazhanbeiguangchang	1	1	2	2	2	2	2	2	2	2	2	2	2	2	2	3	2	2	2
212	Anfajie	2	1	2	2	2	2	2	4	4	3	4	4	4	4	4	4	4	4	4
213	Xinfaxiaoqu	1	1	2	2	2	2	2	2	2	3	3	3	3	3	3	3	3	3	3
214	Kuanchengjie	1	1	2	2	2	2	2	2	3	2	4	3	4	4	4	4	4	4	4
215	Xuanhuajie(Xianfeng Junction)	1	1	2	2	2	2	2	3	3	3	3	3	3	3	3	3	3	3	3
216	Xianfengxiaoqu(Huashanlu Junction)	1	1	1	1	1	1	1	2	2	2	2	1	2	2	2	2	2	2	2
217	Hongqidajie(Xianfeng Junction)	1	1	1	1	1	1	1	2	2	2	2	2	3	3	2	2	3	2	2
218	Hongweilu(Xianfeng Junction)	1	1	1	1	1	1	2	2	2	2	2	2	2	2	2	2	2	2	2
219	Nanzhilu	1	1	1	1	1	2	2	3	3	3	2	2	2	2	2	2	2	2	2
220	Jianzhuyishuguangchang	1	1	1	1	1	1	1	1	1	2	2	2	2	2	2	2	2	2	2
221	Wuyezhongxin	1	1	1	1	1	1	1	2	2	2	2	2	2	2	2	2	2	2	2
222	Harbingongyegaojijigongxuexiao	1	1	2	2	1	1	1	2	2	2	2	2	2	2	2	2	2	2	2
223	Dafanglixiaoqu	1	1	1	1	1	1	1	2	2	2	2	2	2	2	2	2	2	2	2
224	Xinglongbadaojie	1	1	1	1	1	1	1	1	1	1	2	2	2	2	2	2	2	2	2
225	Shilaonianyiyuan	1	1	2	2	1	1	1	2	2	2	3	3	3	3	3	3	3	3	3
226	Daowaigonganfenju	1	1	1	1	1	2	1	2	2	2	2	2	2	2	2	2	2	2	2
227	Huashujie(Hongwei Junction)	1	1	1	2	2	2	2	2	2	2	2	2	2	2	2	2	2	2	2
228	Changjianglu(Nanzhilu Junction)	1	1	1	1	1	1	1	2	2	1	2	2	2	2	2	2	2	2	2
229	Nanzhilu(Hanshuilu Junction)	1	1	1	1	1	1	2	2	2	2	2	2	3	3	3	3	3	3	3
230	Jianguojie(Kangan Junction)	2	2	3	3	3	4	5	5	5	5	6	5	6	6	6	6	6	6	6
231	Hazhan(Tielujie)	1	1	2	2	2	2	2	2	3	2	3	3	3	3	3	3	3	3	3
232	Yiererzhongxue	1	1	1	1	1	1	1	2	2	2	3	3	3	3	3	3	3	3	3
233	Diantajie	1	1	1	1	1	2	1	2	1	2	2	3	2	2	2	2	2	2	2
234	Shengbolanzhongxin(Lianfajie)	1	1	1	1	1	1	1	1	1	1	2	1	2	2	2	2	2	2	2
235	Sankongqiao	1	1	2	2	2	2	2	3	3	3	3	3	5	5	5	5	5	5	5
236	Hashangda(South School District)	1	1	1	1	1	1	1	2	2	2	2	2	4	3	4	3	3	3	3
237	Minanjie	2	2	2	2	2	2	2	2	2	2	3	3	3	3	3	3	3	3	3
238	Xinyanglu	2	2	2	2	2	2	2	3	3	4	4	4	4	4	4	4	4	4	4
239	Alaboguangchang	1	1	2	2	2	2	2	2	2	3	4	4	4	4	4	4	4	4	4

240	Nanxunshisidaojie	1	1	2	2	2	2	2	2	2	3	3	3	3	3	3	3	3	3	3
241	Nanxunjie	1	1	2	2	1	1	2	2	2	3	3	3	3	3	3	3	3	3	3
242	Taiguliudaojie	2	2	2	2	2	2	2	3	3	3	3	3	3	3	3	3	3	3	3
243	Taigutoudaojie	1	1	1	1	1	1	1	2	2	2	2	2	2	2	2	2	2	2	2
244	Wenjunhuayuanxiaoqu	1	1	1	1	1	1	1	2	2	2	2	2	1	2	2	2	2	3	3
245	Wenkujie	1	1	2	2	2	2	2	3	3	3	4	4	4	4	4	4	4	4	4
246	Wenzhengjie	1	1	2	2	2	2	2	4	4	4	5	5	4	4	4	5	4	5	5
247	Shengfunvganbuxueyuan	1	1	2	2	2	2	2	3	3	4	4	5	5	5	5	5	5	5	5
248	Wenchengjie	1	1	1	1	1	1	1	2	2	2	3	3	3	3	3	3	3	3	3
249	Xuandejie	2	2	2	2	3	3	3	3	3	3	5	5	5	5	5	5	5	5	5
250	Xinjishangcheng	1	1	1	1	2	2	1	3	3	3	4	3	4	4	4	4	4	4	4
251	Dingxinsandaojie	1	1	2	2	2	2	2	2	2	2	4	3	4	4	4	4	4	4	4
252	Dafangliweishengfuwuzhan	1	1	1	1	1	1	1	2	2	2	2	2	2	2	2	2	2	2	2
253	ershangdian	1	1	1	1	1	1	2	2	2	3	2	2	2	2	2	2	2	2	2
254	Taiguershidaojie	1	1	2	2	2	2	2	3	3	4	4	4	4	4	4	4	4	4	4
255	Gangtiejie	1	1	1	2	2	2	2	2	3	3	4	3	4	3	3	3	3	3	3
256	Fanghongjiniantagongjiaoshoumozhan	1	1	1	1	1	1	1	1	1	1	2	1	2	2	2	2	2	2	2
257	Zhongyangdajie	1	1	1	1	1	1	1	1	1	2	2	3	3	4	4	4	4	4	4
258	Hongxiajie	1	1	1	1	1	2	2	3	3	3	3	3	3	3	3	3	3	3	3
259	Jingweishiyidaojie	1	1	1	1	1	1	1	2	2	2	2	2	2	2	2	2	2	2	2
260	Aijianlu	1	1	1	1	1	1	1	1	1	1	2	1	2	2	2	2	2	2	2
261	Hezhoujie	1	1	1	1	1	1	2	2	2	2	3	3	4	4	4	4	4	4	4
262	Tielujie	1	1	1	2	2	2	2	2	2	3	3	3	3	3	3	3	3	3	3
263	Wajie	1	1	1	1	1	1	1	2	2	2	3	4	4	3	3	3	3	4	4
264	Xidazhijie	1	1	1	2	1	1	1	2	2	3	3	3	4	4	4	4	4	5	5
265	Hanguangjie(temporary station)	1	1	2	2	2	2	2	2	2	3	4	3	4	4	4	4	4	3	3
266	Wuruijie	1	1	1	1	1	1	1	1	1	2	2	1	2	2	2	2	2	2	2
267	Yikuangjie	1	1	1	1	1	1	1	1	1	2	2	1	2	2	2	2	2	2	2
268	Wangzhaoxincun	1	1	1	2	2	2	2	2	2	2	2	2	2	2	2	2	2	2	2
269	Tielueryuan	1	1	1	1	1	1	1	2	1	2	2	2	2	2	2	2	2	2	2
270	Wangzhaohuochezhan	1	1	1	1	1	1	1	1	1	2	2	1	2	2	2	2	2	2	2
271	Huaqiaomingyuanxiaoqu	1	1	3	2	2	2	2	3	3	4	4	4	4	4	4	4	4	4	4
272	Xiangbinluqiaokou	2	2	3	3	3	3	2	3	4	3	3	3	4	3	3	3	4	4	3
273	Shangyoujie(Zhongyangdajie)	1	1	1	1	1	1	2	2	2	2	3	3	3	3	4	4	4	4	4
274	Jianguogongyuan(Tongdajie)	2	2	3	3	3	3	5	5	5	5	5	5	6	6	6	6	6	5	5
275	Nanpingjie	1	1	2	2	2	2	2	2	2	3	4	3	4	4	4	4	4	4	4
276	Anshengjie(Xinyang Junction)	2	2	2	2	2	2	2	3	3	4	5	5	5	5	5	5	5	5	5
277	Steel Residential Area	1	2	2	2	2	2	3	3	4	4	4	4	5	5	5	5	5	5	5
278	Shiyiyuan	1	1	1	2	2	2	2	2	2	3	3	3	3	5	5	5	5	5	5
279	Zhaolingongyuan	1	1	1	1	1	1	2	1	1	2	3	2	2	3	3	3	3	3	3
280	Jingweijiudaojie	1	1	2	1	1	1	1	2	2	2	3	3	3	3	3	3	3	3	3
281	Anlongjie	1	1	2	2	1	1	1	3	3	2	2	3	3	3	3	3	3	3	3
282	Ansongjie	1	1	2	2	2	2	2	2	2	2	2	2	2	2	2	2	2	2	2
283	Yangmingjie	1	1	2	2	2	2	3	2	2	3	4	3	3	4	3	3	4	4	4
284	Aijianxinchengshoumozhan	1	1	2	2	2	2	2	2	2	2	2	2	2	2	2	2	2	2	2
285	Tongjiangjie	1	1	1	1	1	1	1	2	2	3	3	3	3	3	3	3	3	3	3
286	Shangyoujie	1	1	1	1	1	1	2	2	2	3	3	3	3	3	3	3	3	3	3
287	Hasanzhong	1	1	1	2	2	2	2	3	3	3	5	4	4	5	4	5	5	4	4
288	Guoxinzhengquangongsi	1	1	1	1	1	1	1	2	2	2	1	1	1	1	1	1	1	1	1
289	Youyigong	1	1	1	1	1	1	1	1	1	1	2	1	1	1	1	1	1	1	1
290	Gongchengjie	1	1	1	1	1	1	1	2	2	2	2	2	3	3	3	2	3	2	2
291	Chuangyezhongxin	1	1	1	1	1	1	1	1	1	1	2	2	4	3	5	5	5	5	5
292	Dadaojie	2	1	2	2	2	2	3	3	3	4	4	4	4	4	4	4	4	5	5
293	Yierwuzhong	1	1	2	1	1	1	1	2	2	3	4	3	3	3	3	3	3	3	3
294	Shengdianliyiyuan	1	1	1	1	1	1	2	2	2	2	2	2	4	3	3	3	3	3	3
295	Xiangjianglu	1	1	1	1	1	1	1	1	1	1	1	1	3	3	3	3	3	3	3
296	Heilongjiangshanglv(Hanshuilu)	1	1	1	1	1	1	1	1	1	1	1	1	2	2	3	3	3	4	4
297	Changjianglu	1	1	1	1	1	1	1	1	1	1	2	2	2	2	2	2	2	2	2
298	Huanghelu	1	1	1	1	1	1	1	1	1	1	2	2	2	2	2	2	2	2	2
299	Longyundasha(Lujiajie)	2	2	2	2	2	2	2	3	3	3	4	4	4	2	4	4	4	4	4

300	Zhuozhangouwuerqi	1	1	1	1	1	1	1	1	1	1	2	2	2	2	2	2	2	2	2
301	Shanghaijie	1	1	1	1	1	1	1	1	1	1	2	1	2	2	2	2	2	2	3
302	Jingyuqidaojie(Jingyudajie)	1	1	1	1	1	1	1	2	2	2	3	3	3	2	3	3	3	3	3
303	Taigushisidaojie	2	2	2	2	2	2	2	2	2	3	3	3	3	2	3	3	3	3	3
304	Yuhongxiaoxue	1	1	2	2	2	2	2	2	2	2	3	3	3	2	3	3	3	3	3
305	Benmaqipeicheng(Xianfenglu)	1	1	2	2	2	2	2	2	2	2	3	3	3	2	3	3	3	3	3
306	Shisanzhongxue	1	1	1	1	1	1	1	3	3	3	2	2	2	2	2	2	2	2	2
307	Hatiyuxueyuan	1	1	1	1	1	1	1	1	1	2	2	2	2	2	2	2	2	2	2
308	Haihelu(Xuanqingjie)	1	1	1	1	1	1	1	2	2	2	2	2	2	2	2	2	2	2	2
309	Yixuanguangtongzhaobiaojituan	1	1	1	1	1	1	1	1	1	1	2	2	2	2	2	2	2	2	2
310	Jiusanjituan	1	1	1	1	1	1	1	1	1	1	2	1	2	2	2	2	2	2	2
311	Minjiangxiaoqu	1	1	1	1	1	1	1	2	2	2	2	1	2	2	2	2	2	2	2
312	Taishanxiaoqu	1	1	1	1	1	1	2	2	2	2	2	1	2	2	3	3	3	2	2
313	Hongqixiaoqu	1	1	2	2	2	2	2	3	3	3	4	3	4	2	4	4	4	4	4
314	Huaihelu	2	2	2	2	2	2	2	3	3	3	3	3	3	2	3	3	3	3	3
315	Liaohexiaoqu	1	1	1	1	2	2	2	3	2	3	3	3	2	2	2	2	2	2	3
316	Zhongzhifangzhouyuan	1	1	1	1	1	1	1	2	2	2	2	2	2	2	2	2	2	2	2
317	Haihelu(Xuanqingjie Junction)	1	1	1	1	1	1	1	2	2	2	2	2	2	2	2	2	2	2	2
318	Shizijie	2	2	2	2	2	2	2	3	3	3	4	4	4	2	4	4	4	4	4
319	Shenggonganting	1	1	1	1	1	1	1	2	2	2	2	2	2	2	2	2	2	2	2
320	Wenjunhuayuan	1	1	1	1	1	1	1	2	2	2	2	2	2	2	2	2	2	3	3
321	Xianfenglu(Huashanbei Junction)	1	1	1	1	1	1	2	2	2	2	2	1	2	2	2	2	2	2	2
322	Liaohelu	2	2	2	2	2	2	2	3	3	3	3	3	4	2	4	4	4	4	4
323	Huaihelu(Hongqidajie Junction)	1	1	2	2	2	2	2	3	3	3	3	3	2	2	3	3	3	3	3
324	Huizhanzhongxin	1	1	1	1	1	1	1	1	1	1	1	1	1	2	1	1	1	1	1
325	Hanshuilu(Hongqidajie Junction)	1	1	1	1	1	1	1	2	2	2	2	1	2	2	2	2	2	2	2
326	Hongnanjie	1	1	2	2	2	2	3	3	3	3	2	2	2	2	3	3	3	3	2
327	Hongweilu	1	1	2	2	2	2	2	2	2	3	3	3	3	2	3	3	3	2	2
328	Shieryuan	1	1	1	1	2	2	2	2	2	2	2	1	2	2	2	2	2	2	2
329	Liaohelu(Hongqidajie Junction)	2	2	2	2	2	2	2	3	3	3	3	3	4	2	4	4	4	4	4
330	Huaihelu(Hongqidajiekou)	1	1	2	2	2	2	2	3	3	3	3	3	2	2	3	3	3	3	3
331	Xinyuanxiaoqu	1	1	1	1	1	1	1	1	1	1	2	1	2	2	2	2	2	2	2
332	Hagongdadierxiaoqu	1	1	1	1	1	1	1	1	1	1	2	2	2	2	2	2	2	2	2
333	Hongxianglu	1	1	1	1	1	1	1	1	1	1	1	1	1	2	1	1	1	1	1
334	Sandadonglilu	1	1	1	1	1	1	1	2	2	1	2	1	2	2	2	2	2	2	1
335	Zhaolingongyuanzhan	1	1	2	1	1	2	2	2	2	2	3	3	3	2	3	3	3	3	3
336	Shiyiyuanzhan	1	1	1	2	2	2	2	2	2	3	3	3	3	2	5	5	5	5	5
337	Xiashigushangkeyiyuanzhan	1	1	1	1	1	1	1	2	2	2	2	1	2	2	2	2	2	2	2
338	Hazhanbeiguangchanggongjiaoshoumozhan	1	1	2	2	2	2	2	2	2	2	3	3	3	2	3	3	3	3	3
339	Taihaihuayuanxiaoqu	1	1	1	1	2	2	1	2	2	2	4	3	4	2	4	4	4	4	4
340	Hongboshijiguangchang	1	1	1	1	1	1	1	1	1	1	1	1	1	2	1	1	1	1	1
341	Taishanlu	1	1	1	1	1	1	1	2	2	2	1	1	3	2	2	2	2	3	3
342	Huayuanjie	1	1	1	1	2	1	2	2	2	2	3	3	3	3	3	3	3	3	3
343	Yiererzhong	1	1	1	1	1	1	1	2	2	2	3	3	3	3	3	3	3	3	3
344	Liushunzongheshichang	1	1	1	1	1	1	2	2	2	2	2	2	3	2	3	3	3	4	4
345	Huashanlu(Ganshui Junction)	1	1	1	1	1	1	1	1	1	1	1	1	1	2	2	2	2	2	2
346	Chuangyezhongxin(temporary station)	1	1	1	1	1	1	1	1	1	1	2	2	4	3	5	5	5	5	5
347	Huaihelu(Hongqidajiejiekou)	1	1	1	1	2	2	2	2	2	2	2	2	2	2	2	2	2	2	2
348	Liaohelu(Hongqidajiejiekou)	2	2	2	2	2	2	2	3	3	3	3	3	3	3	3	3	3	3	3
349	Shiguoshuijichaju	1	1	1	1	1	1	2	2	2	3	2	2	2	3	3	3	3	3	2
350	Yimianjie(Senlinjie Junction)	1	1	2	1	1	2	2	2	2	3	2	2	2	3	3	3	3	3	3
351	Alaboguangchang(temporary station)	1	1	2	2	2	2	2	2	2	3	4	4	4	4	4	4	4	4	4
352	Nanxinershidaojie	1	1	2	2	2	2	2	3	3	3	4	3	3	4	3	4	4	4	4
353	Songshanlu	1	1	2	2	2	2	2	3	3	3	3	3	3	3	3	3	3	3	3
354	Guoluchanglinshizhan	1	2	1	2	2	2	2	3	3	3	3	3	4	3	3	3	3	3	3
355	Renliziyuanshichang(Anningjie)	2	2	2	2	3	3	2	3	3	4	4	4	4	5	4	4	4	4	4
356	Wangtongluyingyeting(Ershangdian)	1	1	1	2	2	1	2	2	3	3	2	2	3	2	2	2	2	2	2
357	Kunlunshangcheng(Hanshuilu)	1	1	1	1	1	1	1	1	1	1	2	2	2	2	2	2	2	2	2
358	Haguangdiantai	1	1	1	1	1	1	1	1	1	1	1	1	1	2	2	2	2	2	2
359	Hengdaojie(Gongbinlu)	1	2	2	2	2	3	3	4	4	4	4	3	5	4	5	5	5	5	5

360	Litchi Street	2	2	2	2	3	3	3	3	3	3	5	5	5	5	5	5	5	5	5
361	Xianfengxiaoqu(Huashan Junction)	1	1	1	1	1	1	1	2	2	2	2	1	2	2	2	2	2	2	2
362	Hashangdananxuequ	1	1	2	1	1	1	1	2	2	2	3	3	4	4	4	4	4	4	4
363	Fushunjiedidelijiekou(Fushunxiaoxue)	1	1	2	2	2	2	2	3	3	4	4	4	4	4	4	4	4	4	4
364	Beifangjuchang	1	1	1	1	1	1	1	3	2	3	4	4	4	4	4	4	4	4	4
365	Huashanluxianfenglulukou	1	1	1	1	1	1	1	2	2	2	2	1	2	2	2	2	2	2	2
366	Shengzhijianyuan	1	1	1	1	1	1	1	1	2	1	4	3	4	5	5	5	5	5	5
367	Xinjiangqiaoshichang	1	1	2	2	2	2	2	2	2	3	2	2	3	3	3	3	3	3	3
368	Taipingdajie	1	1	2	1	1	2	2	2	2	2	2	2	2	2	2	2	2	3	3
369	Taipingbeisandaojie	1	1	1	1	1	2	2	2	2	2	2	1	3	2	2	2	2	2	2
370	Taipingnansidaojie	1	1	1	1	1	2	2	2	2	2	2	1	3	2	2	2	3	3	2
371	Baqujie	1	1	1	1	1	1	1	2	1	2	2	1	2	2	2	2	2	2	2
372	Baqujienanjijiekou	1	1	1	1	2	2	2	2	2	3	3	3	2	2	2	2	2	3	2
373	Songpudaqiaonan	1	1	2	2	2	2	2	2	2	3	3	3	3	3	3	3	3	3	3
374	Nanzhixiaoqu	1	1	1	1	1	1	1	1	1	1	1	1	1	1	1	1	1	1	1
375	Wangtongdongzhiluyingyeting(Dongzhilu)	1	1	2	2	2	2	2	3	3	4	2	2	3	3	3	3	3	2	2
376	Yanpingjie	1	1	1	1	1	1	1	2	2	3	4	4	4	4	4	4	4	4	4
377	Dongdazhijie	1	1	1	1	1	1	1	2	2	2	3	3	3	3	3	3	4	3	3
378	Longyundasha	2	2	2	2	2	2	2	3	3	3	4	5	4	4	4	4	4	4	4
379	Hashidananxuequ	1	1	2	2	2	2	2	3	3	3	4	3	5	5	5	5	5	4	4
380	Zhongbeichuncheng	2	2	2	2	2	2	2	5	5	5	5	5	5	5	5	5	5	5	5
381	Zhongxin Department Store	1	2	2	2	2	3	3	4	4	4	4	3	5	5	5	5	5	5	5
382	Xinjiangqiaojie	1	1	2	2	2	2	2	2	2	3	2	2	3	3	3	3	3	3	3
383	Jiashujie	1	1	1	1	1	1	1	2	2	2	3	3	3	3	3	3	3	3	3
384	Hazhantielujie	1	1	2	2	2	2	2	2	3	2	3	3	3	3	3	3	3	3	3
385	Laoshichang	1	1	2	2	2	2	2	3	3	3	3	3	4	3	5	5	4	4	4
386	Linshizhan	1	1	2	2	2	2	2	3	3	4	4	4	4	3	3	3	3	4	4
387	Herunjie	1	2	2	2	2	2	3	2	2	4	4	4	4	4	4	4	4	5	5
388	Daowaiershidaojie	1	1	2	2	2	2	2	3	3	4	4	4	4	4	4	4	4	4	4
389	Daowaikeyunzhan	1	1	1	1	1	2	2	2	2	2	3	2	3	3	3	3	3	3	3
390	Tongdajie(Jianguobeierdaojie)	2	2	3	3	3	3	5	5	5	5	5	5	6	6	6	6	6	5	5
391	Minzhongjie	1	1	2	1	1	1	1	2	2	2	2	2	3	3	3	3	3	3	3
392	Xuanqingxiaoqu	1	1	1	1	1	1	1	1	1	1	2	2	2	2	2	2	2	2	2
393	Shengshizihui	2	2	2	2	3	3	3	3	3	3	4	3	4	4	4	4	4	4	4
394	Wenchangjiezhan	1	1	2	2	2	2	2	2	2	4	5	5	5	5	5	5	5	5	5
395	Hashida(Nanxiaoqu)	1	2	3	2	2	3	3	3	3	3	4	4	5	5	5	5	5	4	4
396	Taiping Second Shop	1	1	1	1	1	2	2	2	3	3	2	2	3	3	2	2	2	2	2
397	Beijingjie(Manzhoulijiekou)	1	1	1	1	1	1	1	2	2	3	3	3	3	3	3	3	3	3	4
398	Hongqidajiegongbinlukou	1	2	2	2	2	3	3	4	4	4	4	3	5	5	5	5	5	5	5
399	Taipingsandaojie	1	1	1	1	1	2	2	2	2	2	2	1	3	2	2	2	2	2	2
400	Taipingsidaojie	1	1	1	1	1	2	2	2	2	2	2	1	3	2	2	2	3	3	2
401	Tongjiangshidigongjiaohuanchengshuniuzhan	1	1	1	1	1	1	1	1	1	1	2	1	1	1	1	1	1	1	1
402	Yimianjie	1	1	2	1	1	2	2	2	2	3	2	2	2	3	3	3	3	3	3
403	Taigushierdaojie	1	1	2	2	2	2	2	3	3	3	3	3	3	3	3	3	3	3	3
404	Taigushiliudaojie	1	1	2	2	3	3	3	3	3	4	3	3	3	3	3	3	3	3	3
405	Xianfenglu(Huashan Junction)	1	1	1	1	2	2	2	2	2	2	2	2	2	2	2	2	2	2	2
406	Harbintiyuxueyuan	1	1	1	1	1	1	1	2	2	2	2	1	2	2	2	2	2	2	2
407	Lvseshipinjiaoyizhongxin	1	1	1	1	1	1	1	1	1	1	2	2	2	2	2	2	2	2	2
408	Hanguangjie	1	1	2	1	2	2	2	2	2	3	4	3	4	4	4	4	4	3	3
409	Qizhengjie	1	1	2	2	2	2	2	3	3	4	4	4	4	4	4	4	4	3	3
410	Boyuexingchenggongjiaoshoumozhan	1	1	1	1	2	2	2	2	2	2	2	1	2	2	2	2	2	2	2
411	Zhaolinjie	1	1	1	1	1	2	1	2	2	2	3	3	3	3	3	3	3	3	3
412	Hongqixiaoqu(temporary station)	1	1	2	2	2	2	2	3	3	3	4	3	4	4	4	4	4	4	4
413	Sandadonglifulu	1	1	1	1	1	1	1	1	1	1	2	2	2	2	2	2	1	1	1
414	Hanshuilu(Hongqidajiekou)	1	1	1	1	1	1	1	1	1	1	1	1	2	3	2	2	2	3	3
415	Kunlunxiaoqu	1	1	1	1	1	1	2	2	2	2	2	1	2	2	2	2	2	2	2
416	Weixinglu	1	1	1	1	2	2	2	2	2	2	2	1	2	2	2	2	2	2	2
417	Wenhuadasha(temporary station)	1	1	1	1	1	1	1	1	1	2	2	1	2	2	2	2	2	2	2
418	Tiandijie	1	1	1	1	1	1	1	2	2	2	2	1	2	2	2	2	2	2	2
419	Yimianjie(temporary station)	1	1	2	1	1	2	2	2	2	3	2	2	2	3	3	3	3	3	3

420	Youyigong(temporary station)	1	1	1	1	1	1	1	1	1	1	2	2	2	1	1	1	1	1	1
421	Xixiangfang(Gongbinlu)	1	1	2	2	2	2	2	3	3	3	3	3	3	3	3	3	3	3	3
422	Huizhanzhongxintiyuchang	1	1	1	1	1	1	1	1	1	1	2	2	2	2	2	2	2	2	2
423	Hazhan(Haichengqiao)	1	1	2	2	2	2	2	2	3	2	3	3	3	3	3	3	3	3	3
424	Nanxunbadaojie	1	1	2	2	1	1	1	2	2	3	3	3	3	3	3	3	3	3	3
425	Jingyujie	1	1	2	2	2	2	2	3	3	4	4	4	4	4	4	4	4	4	4
426	Anfengshangchang	2	2	2	3	3	3	3	3	3	3	3	3	4	3	4	4	3	4	4
427	Jingweijie(Zhongyangdajie)	1	1	2	2	2	2	2	3	3	4	4	3	4	4	4	4	4	4	4
428	Heilongjianggongchengdaxue	1	1	1	1	1	1	2	2	2	3	2	2	2	3	3	3	3	3	3
429	Gexinjiezhan	1	1	2	2	2	2	2	3	3	3	3	3	3	3	3	3	3	3	3
430	Shengdanganshi	1	1	2	2	2	2	2	3	3	3	3	3	3	3	3	3	3	3	3
431	Shizijie(Dachengjiekou)	1	1	1	1	1	1	1	2	2	2	3	2	3	3	3	3	3	3	3
432	Maduanjie	1	1	1	1	2	2	2	3	3	3	3	3	3	3	3	3	3	3	3
433	Hatiyuxueyuandongmen	1	1	1	1	1	1	1	1	1	2	2	1	2	2	2	2	2	2	2
434	Jiangongxinqu	1	1	1	1	1	1	1	1	1	1	2	2	2	3	2	3	3	3	2
435	Hazhan(Haichengqiao)(temporary station)	1	1	2	2	2	2	2	2	3	2	3	3	3	3	3	3	3	3	3
436	Nanxinshisidaojie	2	2	2	2	2	2	2	2	2	3	3	3	3	3	3	3	3	3	3
437	Huangjiahuayuan	1	1	2	2	2	2	2	2	2	3	3	3	3	3	3	3	3	3	3
438	Gongludaqiao(Heluojie)	1	1	1	2	2	2	3	3	3	4	5	5	5	5	5	5	5	6	6
439	Nanjijie(Chengdejie)	1	1	2	2	2	2	2	2	2	4	4	4	4	4	4	4	4	4	4
440	Hongzhuanjie	1	1	1	1	1	1	1	2	2	2	3	3	3	3	3	3	3	3	3
441	Gongchengjie(temporary station)	1	1	1	1	1	1	1	2	2	2	2	2	2	2	2	2	2	2	2
442	Xiangjianglu(Hongqidajie Junction)	1	1	1	1	1	1	1	1	1	1	1	1	3	3	3	3	3	3	3
443	Xiangshunjie	1	2	2	2	2	2	2	3	3	3	3	3	4	4	4	4	4	4	4
444	Xiaofangyiyuan	1	1	1	1	1	1	1	1	1	2	2	1	1	3	2	2	2	3	3
445	Hasanzhong(temporary station)	1	1	1	2	2	2	2	3	3	3	5	4	4	5	4	5	5	4	4
446	Heilongjiangkejixueyuan(Songshanxiaoqu)	1	1	1	1	1	1	1	2	2	2	2	2	2	2	2	2	2	2	2
447	Benmaqipeicheng	1	1	2	2	2	2	2	2	2	2	2	2	2	2	2	2	2	2	2
448	Dongbeiyiyuan	1	1	2	2	2	2	2	2	2	3	4	3	4	4	4	4	4	4	4
449	Fanrongjie	1	1	1	1	1	1	1	1	1	2	2	2	3	3	3	3	3	2	2
450	Taigushisidaojie(temporary station)	1	2	2	2	2	2	2	2	2	3	3	3	3	3	3	3	3	3	3
451	Hongqidajie(Beihuanlu Junction)	1	1	1	1	1	1	2	2	2	2	2	1	2	2	2	2	2	2	2
452	Jingyudajie	1	2	2	2	2	2	2	2	2	3	3	3	3	3	3	3	3	3	3
453	Shengyiyuannangnagfenyuan	1	1	2	2	3	2	3	3	3	3	5	5	5	5	5	5	5	5	5
454	Quxianjie	1	1	1	1	1	2	1	2	2	3	4	4	4	4	4	4	4	4	4
455	Tieludiyixiaoxue	1	1	1	1	1	1	1	2	2	2	3	3	4	3	3	3	3	4	4
456	Wenhuadasha	1	1	1	1	1	1	1	1	1	2	2	1	2	2	2	2	2	2	2
457	Kunlunshangcheng	1	1	1	1	1	1	1	1	1	1	2	2	2	2	2	2	2	2	2
458	Hequjie	1	1	1	2	2	2	2	3	3	3	5	4	5	5	5	5	5	6	6
459	Changjianglunanzhilukou	1	1	1	1	1	1	1	2	2	1	2	2	2	2	2	2	2	2	2
460	Nanzhiluhanshuilukou	1	1	1	1	1	1	2	2	2	2	2	2	3	3	3	3	3	3	3
461	Liushunjie(Situjie Junction)	1	1	1	1	1	1	2	2	2	2	2	2	2	2	3	3	3	4	4
462	Tongdajie(Jianguogongyuan)	1	2	3	3	3	3	5	5	5	5	5	5	6	6	6	6	6	5	5
463	Anshunjie	1	2	2	3	2	2	2	3	3	4	4	4	4	4	4	4	4	4	4
464	Minjianglu(Nanzhilu Junction)	1	1	1	1	1	1	1	2	2	2	2	2	3	4	4	4	2	2	2
465	Xidazhijie(temporary station)	1	1	2	2	2	2	2	4	4	5	4	4	5	5	5	5	5	5	5
466	Shengzhengxie	1	1	1	1	1	1	1	1	1	2	2	2	2	2	2	2	2	2	2
467	Tianmuxiaoqu	1	1	1	1	2	2	2	3	3	3	4	4	4	4	4	4	4	4	4
468	Renlijie	1	1	2	2	2	2	2	3	3	3	4	4	4	4	4	4	4	4	4
469	angtongdongzhiluyingyeting(temporary statio	1	1	1	2	2	2	2	2	3	3	2	2	3	2	2	2	2	2	2
470	Yaocaigongsi	1	1	1	1	1	1	2	2	2	2	1	1	2	2	2	2	2	2	2
471	Huaihelu(temporary station)	2	2	2	2	2	2	2	3	3	3	3	3	3	3	3	3	3	3	3
472	Qianjinlu(Hesongjiekou)	1	2	2	2	2	2	3	3	3	4	4	4	5	5	5	5	5	4	4
473	Xiashigushangyiyuan	1	1	1	1	1	1	1	2	2	2	2	1	2	2	2	2	2	2	2
474	Xiangdianjie	1	1	1	1	1	2	2	2	2	2	2	2	2	2	3	3	3	3	3
475	Huashanlu(Changjianglu Junction)	1	1	1	1	1	1	1	1	1	1	2	2	2	2	2	2	2	2	2
476	Shengbolanzhongxinzhan	1	1	1	1	1	1	1	2	2	3	4	4	4	4	4	4	4	4	4
477	Hongqidajie(Beihuanlukou)	1	1	1	1	1	1	2	2	2	2	2	1	2	2	2	2	2	2	2
478	Lujiajie	1	1	1	1	2	2	2	2	2	2	2	2	2	2	2	2	2	2	2
479	Renhejie	2	2	2	2	1	1	2	2	3	2	3	3	3	3	3	3	3	3	3

480	Huayuanshangdian	1	1	2	2	2	2	2	3	3	3	5	5	5	5	5	5	5	5	5
481	Liaoyangjie	1	1	1	2	2	2	2	3	3	3	4	4	4	4	4	4	4	4	4
482	Ankangjie	1	1	2	2	2	2	2	3	3	3	3	3	3	3	3	3	3	3	3
483	Jingweijie(Gaoyijie)	1	1	2	1	1	1	1	2	2	3	3	3	3	3	3	3	3	3	3
484	Jianguojie(Kanganlulukou)	2	2	3	3	3	4	5	5	5	5	6	5	6	6	6	6	6	6	6
485	Yaoliujiayuan(temporary station)	1	1	2	2	2	2	2	3	3	3	2	2	2	4	3	3	3	3	3
486	Shierzhongxue	1	1	1	1	1	2	2	2	2	2	2	1	2	2	2	2	1	2	2
487	Lujiajie(Shizijie Junction)	2	2	2	2	2	2	2	3	3	3	4	4	4	4	4	4	4	4	4
488	Renhejie(Jianxinjiekou)	1	1	1	1	1	1	1	2	2	2	2	2	2	2	2	3	2	2	2
489	Harbin Print Center	1	2	2	3	2	3	3	4	4	5	5	6	5	5	5	5	5	5	5
490	Miaopujie	1	1	1	1	1	1	1	1	1	1	1	1	1	1	1	1	1	1	1
491	Shierzhong	1	1	1	1	1	2	2	2	2	2	2	1	2	2	2	2	1	2	2
492	Bowuguanzhan(temporary station)	1	1	1	1	1	1	1	2	2	3	4	4	4	4	4	4	4	4	4
493	Hongqidajie	1	1	1	1	1	2	2	3	3	2	2	2	2	2	2	2	2	2	2
494	Tielujie(Shangfangjie)	1	1	1	2	2	2	2	2	2	3	2	2	2	2	2	2	2	2	2
495	Tielujie(temporary station)	1	1	1	2	2	2	2	2	2	3	3	3	3	3	3	3	3	3	3
496	Tielujieqiaobeijiekou	1	1	1	1	1	1	1	1	1	2	2	1	3	3	3	3	3	3	3
497	Tielujieqingmingerdaokou	1	1	1	2	2	2	2	2	2	3	3	3	4	4	4	4	4	4	4
498	Binjianghuozhan	1	1	2	2	2	2	2	3	3	3	3	3	3	3	3	3	3	3	3
499	Huochezhan	1	1	2	2	2	2	2	4	4	3	4	4	4	4	4	4	4	4	4
