# Supplementary material for: Optimizing urban bus network based on spatial matching patterns for sustainable transportation: A case study in Harbin, China
Source: PLoS One. 2024 Oct 28;19(10):e0312803. doi: 10.1371/journal.pone.0312803 (PMC11515997; doi:10.1371/journal.pone.0312803)
Supplement: S4 Table — (PDF) [file pone.0312803.s004.pdf]

S4 Table. Traffic condition data

Number	Bus Station	6:30	6:40	6:50	7:00	7:10	7:20	7:30	7:40	7:50	8:00	8:10	8:20	8:30	8:40	8:50	9:00	9:10	9:20	9:30
1	Cheliangchangwenhuagong	1	1	1	1	1	1	1	1	1	1	1	1	1	1	1	1	1	1	1
2	Jingweishidaojie	1	1	1	1	1	1	1	1	1	1	1	1	1	1	1	1	1	1	1
3	Jingweishidaojie	1	1	1	1	1	1	1	1	1	1	1	1	1	1	1	1	1	1	1
4	Daolishierdaojie	1	1	1	1	1	1	1	1	1	1	1	1	1	1	1	1	1	1	1
5	Zhaolinjie	1	1	1	1	1	1	1	1	1	1	1	1	1	1	1	1	1	1	1
6	Maimaijie	1	1	1	1	1	1	1	1	1	1	1	1	1	1	1	1	1	1	1
7	Nanmalu	1	1	1	1	1	1	1	1	1	1	1	1	1	1	1	1	1	1	1
8	Chengdeguangchang	1	1	1	1	1	1	1	1	3	1	1	1	1	1	1	1	1	1	1
9	Jingyangjie(temporary station)	1	1	1	1	1	1	1	1	1	1	1	1	1	1	1	1	1	1	1
10	Daowaisandaojie(temporary station)	1	1	1	1	1	1	1	1	1	1	1	1	1	1	1	1	1	1	1
11	Daowaiqidaojie(temporary station)	1	1	1	1	1	1	1	1	1	1	1	1	1	1	1	1	1	1	1
12	Daowaishierdaojie(temporary station)	1	1	1	1	1	1	1	1	1	2	2	2	2	1	1	1	1	1	1
13	Shisiyuan	1	1	1	1	1	1	1	1	1	1	1	1	1	1	1	1	1	1	1
14	Jingyuershidaolu	1	1	1	1	1	1	1	1	1	1	1	1	1	1	1	1	1	1	1
15	Songpudaqiao	1	1	1	1	1	1	1	2	1	1	1	1	1	1	1	1	1	1	1
16	Gengxinjie	1	1	1	1	1	1	1	1	1	1	1	1	1	1	1	1	1	1	1
17	Youshichang	1	1	1	1	1	1	1	1	1	1	1	1	1	1	1	2	1	1	1
18	Mucaishichang	1	1	1	1	1	1	1	1	1	1	1	1	1	1	2	2	2	2	3
19	Gangwuju	1	1	1	1	1	1	1	1	1	1	1	1	1	1	1	1	1	1	1
20	Dianjichang	1	1	1	1	1	1	1	1	2	2	1	1	1	3	3	3	3	1	1
21	Leyuanjie	1	1	1	1	1	1	1	1	2	2	3	3	3	4	3	3	3	3	3
22	Lesongguangchang	1	1	1	1	1	1	1	1	1	3	3	3	3	4	4	4	3	3	3
23	Forestry University	1	1	1	1	1	3	3	3	3	3	3	3	3	4	4	4	3	3	3
24	Linyezongyiyuan	1	1	1	2	2	3	3	3	3	3	3	3	3	4	4	4	3	3	3
25	Linxinglu	1	1	4	3	3	3	2	3	3	3	3	3	3	4	4	4	3	3	3
26	Harbin Normal University (South Campus)	1	3	2	3	2	3	3	2	2	2	2	2	3	3	4	4	3	3	2
27	Hexinglu	1	1	1	2	2	2	2	2	2	1	2	2	3	3	3	2	2	2	2
28	Tongdajie	1	1	1	1	1	1	1	1	3	1	1	2	2	4	2	2	2	2	3
29	Xidaqiao	1	1	1	1	1	1	3	4	2	1	1	1	3	1	1	1	1	1	1
30	Hagongda	1	1	1	1	3	2	3	3	2	2	3	3	4	2	1	1	1	1	1
31	Hatieluju	1	1	1	1	2	1	2	3	2	1	1	1	1	1	1	1	1	1	1
32	Shengbolanzhongxin	1	1	1	1	1	1	1	1	1	2	2	3	2	2	1	1	1	1	1
33	Qiulingongsi	1	1	1	1	1	1	1	1	1	1	1	1	3	2	2	2	2	2	3
34	Yidayiyuan	1	1	1	1	1	1	1	1	1	3	2	2	2	2	2	2	3	2	2
35	Yanchang	1	1	1	1	1	1	1	1	1	1	1	1	1	1	1	1	1	1	1
36	Hazhan	1	1	1	1	1	1	1	1	1	1	1	1	1	1	1	1	1	1	1
37	Shigushangyiyuan	1	1	1	1	1	1	1	1	1	1	1	1	1	1	1	1	1	1	1
38	Hesongjie	1	1	1	1	1	1	1	3	3	3	4	4	3	3	3	3	3	3	3
39	Hezhengxiaoqu	1	1	1	1	1	1	1	1	1	1	1	1	1	1	1	3	1	3	1
40	Hegujie	1	1	1	1	1	1	1	1	1	1	1	1	1	1	1	1	1	1	1
41	Heliangjie	1	1	1	2	1	1	1	1	1	1	1	1	1	2	1	1	3	4	3
42	Highway Bridge Station	1	1	1	1	1	1	1	1	1	3	3	3	2	2	3	3	3	2	3
43	Shehuikexueyuan	1	1	1	1	1	2	2	2	2	3	3	3	2	2	2	2	1	1	1
44	Baoyedasha	1	1	1	1	1	1	3	2	2	2	3	3	2	3	2	2	1	1	3
45	Shidishuiju	1	1	1	1	1	1	1	2	1	1	1	1	1	1	1	1	1	1	1
46	Shirenda	1	1	1	1	1	1	1	1	1	1	1	1	1	1	1	1	1	1	1
47	Fanghongjinianta(Youyilu)	1	1	1	1	1	1	1	1	1	1	1	1	1	1	1	1	1	1	1
48	Ertongyiyuan	1	1	1	1	1	1	1	1	1	1	1	1	1	1	1	1	1	1	1
49	Shilaoniandaxue	1	1	1	1	1	1	1	1	1	1	1	3	1	1	1	1	1	1	1
50	Daowaisandaojie	1	1	1	1	1	1	1	1	1	1	1	1	1	1	1	1	1	1	1
51	Daowaiqidaojie	1	1	1	1	1	1	1	1	1	1	1	1	1	1	1	1	1	1	1
52	Daowaishierdaojie	1	1	1	1	1	1	1	1	1	2	2	2	2	1	1	1	1	1	1
53	Daowaishiliudaojie	1	1	1	1	1	1	1	1	3	3	3	2	1	1	1	1	1	1	1
54	Daotaifu	1	1	1	1	1	1	1	1	1	1	1	1	1	1	1	1	1	1	1
55	Taocixiaoqu(temporary station)	1	1	1	1	1	1	1	1	1	1	1	1	1	1	1	1	1	1	1
56	Taocixiaoqu	1	1	1	1	1	1	1	1	1	1	1	1	1	1	1	1	1	1	1
57	Jingweishierdaojie	1	1	1	1	1	1	1	1	1	1	1	1	1	1	1	1	1	1	1
58	Gaoyijie	1	1	1	1	1	1	1	1	1	1	1	1	1	1	1	1	1	1	1
59	Dongfengjie	1	1	1	1	1	1	1	1	1	1	1	1	1	1	1	1	1	1	1

60	Jianzhuyishuguangchangzhan	1	1	1	1	1	1	1	1	1	1	1	1	1	1	1	1	1	1	1
61	Bowuguanzhan	1	1	1	1	1	1	1	1	1	1	1	1	1	1	1	1	1	1	1
62	Ertonggongyuan	1	1	1	1	1	1	1	1	2	4	3	2	1	2	1	1	1	1	1
63	Dachengjie	1	1	1	1	1	1	1	1	1	1	1	2	2	2	2	2	1	1	1
64	Xuanhuajie(Xianfenglu Junction)	1	1	1	1	1	1	1	1	2	1	1	1	2	1	1	1	1	1	1
65	Xianfengxiaoqu	1	1	1	1	1	1	1	1	1	2	1	1	1	1	1	1	1	1	1
66	Xianfenglu(Huashanlu Junction)	1	1	1	1	1	1	2	4	2	1	1	1	1	1	1	1	1	1	1
67	Songshanxiaoqu	1	1	1	1	1	1	2	4	2	1	1	1	1	1	1	1	1	1	1
68	Hongqidajie(Xianfenglu Junction)	1	1	1	1	1	1	1	1	1	1	1	1	1	1	1	1	1	1	1
69	Hongqidajie(Hongweilu Junction)	1	1	1	1	1	1	1	1	1	1	1	1	1	1	1	1	1	1	1
70	Shieryuan(temporary station)	1	1	1	1	1	1	1	1	1	1	1	1	1	1	1	1	1	1	1
71	Boyuexingcheng	1	1	1	1	1	1	1	1	1	1	1	1	1	1	1	1	1	1	1
72	Qianjinlu	1	1	1	1	1	1	1	1	1	1	1	1	3	3	3	3	3	2	2
73	Jianguojie	1	1	1	1	1	1	1	1	3	2	2	2	3	2	2	2	1	1	1
74	Anhong Street	1	1	1	1	1	2	2	2	2	2	2	1	2	2	2	2	1	1	1
75	Anshengjie	1	1	1	1	1	1	1	1	1	1	1	3	3	2	2	2	1	1	1
76	Beianjie	1	1	1	1	1	1	1	1	2	1	1	2	2	2	2	2	1	1	2
77	Jingweijie(temporary station)	1	1	1	1	1	1	1	1	1	3	2	2	3	1	1	2	1	1	1
78	Hazhanbeiguangchang(temporary station)	1	1	1	1	1	1	1	1	1	1	1	1	1	1	1	1	1	1	1
79	Bowuzhan	1	1	1	1	1	1	1	1	1	1	1	1	1	1	1	1	1	1	1
80	Hagongchengdaxue	1	1	1	1	1	1	2	3	3	2	2	1	1	1	1	1	1	1	1
81	Nantongdajie	1	1	1	1	1	1	1	3	3	3	3	3	3	1	1	1	1	2	1
82	Taipingqiao	1	1	1	1	1	2	3	4	2	4	1	4	3	4	4	4	4	4	4
83	Heilongjianggongchengxueyuan	1	1	1	1	1	1	1	1	1	1	1	1	1	1	1	1	1	1	1
84	Daxinxiecheng	1	1	1	1	1	1	1	1	1	1	1	1	1	1	1	1	1	1	1
85	Honghexiaoqu	1	1	1	1	1	1	1	1	1	1	1	1	1	1	1	1	1	1	1
86	Hongpingxiaoqu	1	1	1	1	1	1	1	1	1	1	1	1	1	1	1	1	1	1	1
87	Hongqijiajucheng	1	1	1	1	1	1	1	1	1	1	1	1	1	1	1	1	1	1	2
88	Hongweilu(Xianfenglu Junction)	1	1	1	1	1	1	1	1	1	1	1	1	1	1	1	1	1	1	1
89	Hepingxiaoqu	1	1	1	1	1	1	1	2	3	1	1	1	1	1	1	1	1	1	1
90	Xinhengguwancheng	1	1	1	1	1	2	2	3	2	1	1	2	1	1	1	1	1	1	1
91	Daolixierdaojie	1	1	1	1	1	1	1	1	1	1	1	1	1	1	1	1	1	1	1
92	Jingweixiao	1	1	1	1	1	1	1	1	1	1	1	1	1	1	1	1	1	1	1
93	Anfengjie	1	1	1	1	1	1	1	1	1	1	1	1	1	1	1	1	1	1	1
94	Anhejie	1	1	1	1	1	1	1	1	1	1	1	1	1	1	1	1	1	1	1
95	Jianguogongyuan	1	1	1	1	1	1	1	3	3	4	4	4	2	2	1	1	1	1	1
96	Shizhongyiyiyuan	1	1	1	1	1	1	2	2	1	1	1	3	3	4	3	3	1	1	3
97	Shengkangfuzhongxin	1	1	1	1	1	1	1	1	1	1	1	1	1	1	1	1	1	1	1
98	Nanzhilu(Hanshui Junction)	1	1	1	1	1	1	1	1	1	1	1	1	1	1	1	1	1	1	1
99	Jianchengchang	1	1	1	1	1	1	1	1	1	4	3	2	2	2	2	3	1	1	1
100	Shiyizhongxue	1	1	1	1	1	1	3	3	3	1	1	1	1	2	1	1	1	1	1
101	Jianbeilu	1	1	1	1	1	1	1	1	1	1	1	1	1	2	1	2	1	1	1
102	Zhujianglu	1	1	1	1	1	1	1	1	1	1	1	1	1	1	1	1	1	1	1
103	Shizhijianyuan	1	1	1	1	1	1	1	1	1	1	1	1	1	2	1	1	1	1	2
104	Shengfuerzhongxin	1	1	1	1	1	1	1	1	1	1	1	1	1	1	1	1	1	1	1
105	Liushunjiesitujiekou	1	1	1	1	1	1	1	1	1	1	1	1	1	1	1	1	1	1	1
106	Zuguoyiyaoyanjiusuo	1	1	1	1	1	1	1	1	1	2	3	3	4	4	3	3	2	1	1
107	Xiangbinlu	1	1	1	1	1	1	3	3	2	2	2	2	2	4	3	3	2	1	1
108	Yamachang	1	1	1	2	1	1	1	1	1	1	1	3	3	1	1	1	1	1	2
109	Liangjuchang	1	1	1	1	1	1	1	1	1	1	1	1	2	1	1	1	1	1	3
110	Minshenglu	1	1	1	1	1	1	1	1	1	1	1	1	1	1	1	1	1	1	1
111	Wanjiachengshangchang	1	1	2	2	3	3	3	3	1	1	1	1	1	3	3	3	3	3	3
112	Wenjingjie	1	1	1	2	1	1	1	1	1	1	1	2	1	1	1	1	1	1	1
113	Fadianchang	1	1	1	1	1	1	1	1	1	1	1	1	1	1	1	1	1	1	1
114	Shigonggongdianchezonggongsi	1	1	3	3	1	1	1	1	1	1	1	2	1	1	1	1	1	1	1
115	Shengshiyanzhongxue(Hegoujie)	1	1	1	1	1	1	1	1	1	1	1	1	1	1	1	1	1	1	1
116	Manzhouliljie	1	1	1	1	1	1	1	1	1	1	1	4	2	1	1	1	1	1	1
117	Hayibai	1	1	1	1	1	1	1	1	1	1	1	1	1	1	1	1	1	1	1
118	Zhongguorenshoubaoxiangongsi	1	1	1	1	1	1	1	1	1	1	1	1	1	1	1	1	1	1	1
119	Daolisandaojie	1	1	1	1	1	1	1	1	1	1	1	1	1	1	1	1	1	1	1

120	Tongjiangshidigongjiaoshuniuzhan	1	1	1	1	1	1	1	1	1	1	1	1	1	1	1	1	1	1	1
121	Gongbin Road	1	1	1	1	2	2	2	2	3	2	3	3	2	2	1	2	1	1	2
122	Xiangbinxiaoxue	1	1	1	1	2	2	3	3	3	2	3	3	2	2	2	2	1	1	2
123	Hongqidajie(Gongbin Junction)	1	1	1	1	1	2	3	3	3	3	3	3	3	2	2	2	1	1	1
124	Hengdaojie	1	1	1	1	1	1	1	1	1	1	1	1	1	1	1	1	1	1	1
125	Caiyijie	1	1	1	1	1	1	1	1	1	1	1	1	1	1	1	1	1	1	1
126	Xiangfangwandaguangchang	1	1	1	1	1	1	1	1	1	1	1	1	1	1	1	1	1	1	1
127	Hanshuilu	1	1	1	1	1	1	1	1	1	1	1	1	1	1	1	1	1	1	2
128	shengrencaishicang	1	1	1	1	2	1	1	1	1	1	1	1	1	1	1	1	1	1	1
129	Zhongxuanjie	1	1	1	1	1	1	1	1	1	2	2	1	1	1	1	1	1	1	1
130	Xuanhuajie	1	1	1	1	1	1	1	3	2	2	2	1	1	1	2	1	1	2	1
131	Xuanhuajie(Lizhijie)	1	1	1	1	1	1	1	1	1	1	1	1	1	1	1	1	1	1	1
132	Gexinjie	1	1	1	1	1	1	1	1	1	1	1	1	1	1	1	1	1	1	1
133	Wenchangjie(temporary station)	1	1	1	1	3	3	3	3	2	3	3	2	1	1	1	1	1	1	2
134	Dongbeiyiyuan(temporary station)	1	1	1	1	1	1	1	1	1	1	1	1	1	1	1	1	1	1	1
135	Wenmingjie	1	1	1	1	1	1	1	1	1	1	1	2	1	1	1	1	1	1	1
136	Gongsijie	1	1	1	1	1	1	1	1	1	1	1	1	1	1	1	1	1	1	1
137	Fuhuaxiaoqu(Fanrongjie)	1	1	1	1	1	1	1	1	1	1	1	1	1	1	1	1	1	1	1
138	Fuhuaxiaoqu	1	1	1	1	1	1	1	2	2	2	3	3	4	4	4	4	3	3	3
139	Fushunjie	1	1	1	1	1	3	3	3	3	3	3	2	3	3	2	3	3	2	3
140	Anfaqiao	1	1	1	1	1	1	3	2	3	3	3	3	3	2	2	1	1	3	2
141	Andejie	1	1	1	1	1	2	3	2	3	2	3	3	2	2	1	1	1	1	2
142	anjianxinchenggongjiaoshoumozhan	1	1	1	1	1	2	1	2	2	2	2	1	1	1	1	1	1	1	1
143	Shengzhongyiyaodaxue	1	1	1	1	1	1	1	1	3	4	2	2	2	3	2	2	1	1	1
144	Hepingqiao	1	1	1	4	3	3	2	3	3	4	4	4	4	3	4	3	2	2	3
145	Shengwenlian	1	1	1	1	2	1	1	1	3	1	1	1	1	1	1	1	1	1	1
146	Wenfujie	1	1	1	1	1	1	1	1	1	1	1	1	1	1	1	1	1	1	1
147	Shengdanganguan	1	1	1	1	3	3	3	3	2	2	2	1	1	2	1	1	1	1	1
148	Jianshejie	1	1	1	1	2	2	2	2	3	3	3	3	2	2	2	2	1	2	2
149	Xianfenglu	1	1	1	1	1	1	1	1	1	1	1	1	1	1	1	1	1	1	1
150	Shengyaocaigongsi	1	1	1	1	1	1	1	1	1	1	1	1	1	1	1	1	1	1	1
151	Nanzhishangchang	1	1	1	1	1	1	1	1	1	1	1	1	1	1	1	1	1	1	1
152	Huashujie	1	1	1	1	1	1	1	1	1	1	1	1	1	1	1	1	1	1	1
153	Wangtongdongzhiluyingyeting	1	1	1	1	1	2	1	1	1	1	1	1	1	1	2	2	2	2	2
154	Jingyangjie	1	1	1	1	1	1	1	1	1	1	1	1	1	1	1	1	1	1	1
155	Kaiyuanerdaojie	1	1	1	1	1	1	1	1	1	1	1	1	1	1	1	1	1	1	1
156	Shengzhengfu	1	1	1	1	1	1	1	1	1	1	1	1	1	2	2	1	1	2	2
157	Wenchangjie	1	1	1	1	1	3	3	3	2	3	3	2	1	2	2	1	2	1	2
158	Gongrenwenhuagong	1	1	1	1	1	1	1	1	1	1	1	2	2	2	2	2	1	1	1
159	Hazhanbeiguangchang(Haichengqiao)	1	1	1	1	1	1	1	1	1	1	1	1	1	1	1	1	1	1	1
160	Tianyilanwangongjiaoshoumozhan	1	1	1	1	1	1	1	1	1	1	1	1	1	1	1	1	1	1	1
161	Dafashichang	1	1	1	1	2	2	3	2	2	2	3	4	4	4	3	3	2	2	2
162	Kanganlu	1	1	1	1	1	3	3	3	3	1	1	1	1	2	2	2	1	1	1
163	Zhongxinyiyuan	1	1	1	1	1	1	1	2	2	2	2	1	1	1	2	2	2	2	2
164	Anshengjie(Xinyanglu Junction)	1	1	1	1	1	1	1	1	1	1	1	3	3	2	2	2	1	1	1
165	Jingweijie	1	1	1	1	1	1	1	1	1	1	1	2	1	1	1	1	1	1	1
166	Ertongyiyuanzhan	1	1	1	1	1	1	1	1	1	1	1	1	1	1	1	1	1	1	1
167	Jiuzhangongjiaoshoumozhan	1	1	1	1	1	1	1	1	1	1	1	1	1	1	1	1	1	1	1
168	Fanghongjinaintazhan	1	1	1	1	1	1	1	1	1	1	1	1	1	1	1	1	1	1	1
169	Ertongdianyingyuan	1	1	1	1	1	1	1	1	1	1	1	1	1	1	1	1	1	1	1
170	Hazhanbeiguangchang(Jihongjie)	1	1	1	1	1	1	1	1	1	2	2	1	1	1	1	1	1	1	1
171	Swan Hote	1	1	1	1	1	2	4	2	2	3	3	4	3	3	3	3	2	2	1
172	Shengyiyuan	1	1	1	1	1	1	2	2	1	2	2	1	2	1	2	2	2	2	2
173	Xixiangfang	1	1	1	1	1	1	1	1	1	3	3	3	3	2	2	2	1	1	1
174	Hongqidajie(Gongbinlu Junction)	1	1	1	1	1	2	3	3	3	3	3	3	3	2	2	2	1	1	1
175	Shichuanranbingyiyuan	1	1	1	1	1	2	3	3	3	2	2	3	3	2	2	2	1	2	1
176	Jingweiliudaojie	1	1	1	1	1	1	1	1	1	1	1	1	1	1	1	1	1	1	1
177	Jingweisandaojie	1	1	1	1	1	1	1	1	1	1	1	1	1	1	1	1	1	1	1
178	Lieshiguan	1	1	1	1	1	1	1	1	2	3	2	2	2	1	1	1	1	1	1
179	Hongtujie	1	1	1	1	1	2	3	3	1	1	1	1	1	1	1	1	1	1	1

180	Yaoliujiayuan	1	1	1	1	1	1	1	1	1	1	1	1	1	1	1	1	1	1	1
181	Shiyinhang	1	1	1	1	1	2	1	1	2	2	2	2	2	2	2	2	1	1	1
182	Wangfujingfanyuecheng	1	1	1	1	1	1	1	1	1	1	1	1	1	1	1	1	1	1	1
183	Shibaqutiyuzhongxin	1	1	1	1	1	1	1	1	1	1	1	1	1	1	1	1	1	1	1
184	Shizhufanggongjijinzhongxin	1	1	1	1	1	1	1	1	1	1	1	1	1	1	1	1	1	1	1
185	Nanjishichang	1	1	1	1	1	1	1	1	1	1	2	2	2	1	1	1	1	1	1
186	Nanjijie	1	1	1	1	1	2	2	2	3	3	3	3	3	1	1	1	1	1	2
187	Binjiangzhan	1	1	1	1	1	1	1	1	1	1	1	2	2	1	1	1	3	2	3
188	Binjiangxinchenggongjiaoshoumozhan	1	1	1	1	1	1	1	1	1	1	1	1	1	1	1	1	1	1	1
189	Longjiangguoluchang	1	1	1	1	1	1	1	1	1	1	1	1	1	1	1	1	1	1	1
190	Daowaishibadaojie	1	1	1	1	1	1	1	1	1	1	1	1	1	1	1	1	1	1	1
191	Shidisiyiyuan	1	1	1	1	1	1	1	1	1	1	1	1	1	1	1	1	1	1	1
192	Dongbeiyiyuanzhan	1	1	1	1	1	1	1	1	1	1	1	1	1	1	1	1	1	1	1
193	Wenhuajie	1	1	1	2	3	3	3	3	1	1	1	1	1	1	2	2	2	2	1
194	Jianguojie(Kanganlu Junction)	1	1	1	1	1	3	2	3	3	2	3	3	4	4	3	3	3	3	3
195	Wujing Hospital	1	1	1	1	2	3	3	3	3	3	3	3	4	4	3	3	3	3	2
196	Sishiqizhongxue	1	1	1	2	2	1	1	1	1	1	1	2	3	3	3	2	3	2	2
197	Hazhanbeiguangchang(Haichengqiao)	1	1	1	1	1	1	1	1	1	1	1	1	1	1	1	1	1	1	1
198	Boligangyanjiusuo	1	1	1	1	1	1	1	1	1	1	1	1	1	1	1	1	1	1	1
199	Jianbeixiaoqu	1	1	1	1	1	1	1	1	1	1	1	1	2	1	1	1	1	1	1
200	Xiaofangyiyuan(temporary station)	1	1	1	1	1	1	1	1	1	1	1	1	1	1	1	1	1	1	1
201	Hanyayinhang	1	1	1	1	1	1	1	1	1	1	1	1	2	1	1	1	1	1	1
202	Yushanlu	1	1	1	1	1	1	1	1	1	1	1	1	1	1	1	1	1	1	1
203	Longta	1	1	1	1	1	1	1	1	1	1	1	1	1	1	1	1	1	1	1
204	Heilongjiangdongbeiyiyuan	1	1	1	1	1	1	1	1	1	1	1	1	1	1	1	1	1	1	1
205	Xiaohongzhongxue	1	1	1	1	1	1	1	1	1	1	1	4	2	1	1	1	1	1	1
206	Songhuajiangjie	1	1	1	1	1	1	1	1	1	1	1	1	1	1	1	1	1	1	1
207	Jiaohuaguangchang	1	1	1	2	1	1	1	2	2	2	2	1	3	4	4	4	3	2	2
208	Fushunxiaoxue	1	1	1	1	1	1	1	1	1	1	1	1	1	1	1	1	1	1	1
209	Shirencaishichang	1	1	1	1	1	1	1	1	1	1	1	1	1	1	1	1	1	1	1
210	Renliziyuanshichang	1	1	1	1	1	1	1	1	1	1	1	1	1	1	1	1	1	1	1
211	Hazhanbeiguangchang	1	1	1	1	1	1	1	1	1	1	1	1	1	1	1	1	1	1	1
212	Anfajie	1	1	1	1	1	1	1	1	1	1	1	1	1	1	1	1	1	1	1
213	Xinfaxiaoqu	1	1	1	1	1	1	1	1	1	2	1	1	3	1	2	2	1	1	1
214	Kuanchengjie	1	1	1	1	1	1	1	1	1	2	3	3	3	2	2	2	1	1	1
215	Xuanhuajie(Xianfeng Junction)	1	1	1	1	1	1	1	1	2	1	1	1	2	1	1	1	1	1	1
216	Xianfengxiaoqu(Huashanlu Junction)	1	1	1	1	1	1	2	4	2	1	1	1	1	1	1	1	1	1	1
217	Hongqidajie(Xianfeng Junction)	1	1	1	1	1	1	1	1	1	1	1	1	1	1	1	1	1	1	1
218	Hongweilu(Xianfeng Junction)	1	1	1	1	1	1	1	1	1	1	1	1	1	1	1	1	1	1	1
219	Nanzhilu	1	1	1	1	1	1	1	1	1	1	1	1	1	1	1	1	1	1	1
220	Jianzhuyishuguangchang	1	1	1	1	1	1	1	1	1	1	1	1	1	1	1	1	1	1	1
221	Wuyezhongxin	1	1	1	1	1	1	1	1	1	1	1	1	1	1	1	1	1	1	1
222	Harbingongyegaojijigongxuexiao	1	1	1	1	1	1	1	1	1	1	1	1	1	1	1	1	1	1	1
223	Dafanglixiaoqu	1	1	1	1	1	1	1	1	1	1	1	1	1	1	1	1	1	1	1
224	Xinglongbadaojie	1	1	1	1	1	1	1	1	1	2	3	4	2	2	2	2	1	1	1
225	Shilaonianyiyuan	1	1	1	1	1	1	1	1	2	3	3	2	3	3	2	3	2	1	1
226	Daowaigonganfenju	1	1	1	1	1	1	1	1	1	1	1	1	1	1	1	1	1	1	1
227	Huashujie(Hongwei Junction)	1	1	1	1	1	1	1	1	1	1	1	1	1	1	1	1	1	1	1
228	Changjianglu(Nanzhilu Junction)	1	1	1	1	1	1	1	1	1	1	1	1	1	1	1	1	1	1	1
229	Nanzhilu(Hanshuilu Junction)	1	1	1	1	1	1	1	1	1	1	1	1	1	1	1	1	1	1	1
230	Jianguojie(Kangan Junction)	1	1	1	1	1	3	2	3	3	2	3	3	4	4	3	3	3	3	3
231	Hazhan(Tielujie)	1	1	1	1	1	1	1	1	1	1	1	1	1	1	1	1	1	1	1
232	Yiererzhongxue	1	2	1	1	1	1	2	2	3	2	3	3	3	3	2	2	1	1	1
233	Diantajie	1	1	1	2	1	1	1	1	1	1	1	3	3	3	3	2	2	2	1
234	Shengbolanzhongxin(Lianfajie)	1	1	1	1	1	1	1	1	1	1	1	2	1	1	1	1	1	1	1
235	Sankongqiao	1	1	1	1	1	1	1	1	1	1	1	1	1	1	1	1	1	2	1
236	Hashangda(South School District)	1	1	1	1	1	1	1	1	1	4	1	1	1	1	1	1	2	1	2
237	Minanjie	1	1	1	1	1	1	1	1	1	1	1	1	1	1	1	1	1	1	1
238	Xinyanglu	1	1	1	1	1	1	1	2	2	2	2	1	4	1	1	2	1	2	3
239	Alaboguangchang	1	1	1	1	1	1	1	1	1	1	1	1	1	1	1	1	1	1	1

240	Nanxunshisidaojie	1	1	1	1	1	1	1	1	1	1	1	1	1	1	1	1	1	1	1
241	Nanxunjie	1	1	1	1	1	1	1	1	1	1	1	1	1	1	1	1	1	1	1
242	Taiguliudaojie	1	1	1	1	1	1	1	1	1	1	1	1	1	1	1	1	1	1	1
243	Taigutoudaojie	1	1	1	1	1	1	1	1	1	1	1	1	1	1	1	1	1	1	1
244	Wenjunhuayuanxiaoqu	1	1	1	1	1	1	2	2	2	3	3	3	4	3	4	4	3	3	3
245	Wenkujie	1	1	1	1	1	1	1	2	1	1	1	2	2	2	2	2	3	2	2
246	Wenzhengjie	1	1	1	1	1	1	3	3	3	3	3	3	3	3	3	4	3	4	4
247	Shengfunvganbuxueyuan	1	1	1	1	1	3	3	3	3	3	3	3	3	3	3	4	3	4	3
248	Wenchengjie	1	1	1	1	1	1	1	1	1	1	1	1	1	1	1	1	1	1	1
249	Xuandejie	1	1	1	1	1	1	1	1	1	1	1	1	1	1	1	1	1	1	1
250	Xinjishangcheng	1	1	1	1	1	1	1	1	2	2	2	2	1	2	1	1	1	1	1
251	Dingxinsandaojie	1	1	1	1	1	1	1	1	1	3	1	1	3	2	2	2	1	1	1
252	Dafangliweishengfuwuzhan	1	1	1	1	1	1	1	1	1	1	1	1	1	1	1	1	1	1	1
253	ershangdian	1	1	1	1	1	1	1	1	1	1	1	1	1	1	1	1	1	1	1
254	Taiguershidaojie	1	1	1	1	1	1	2	3	1	1	1	1	1	3	1	1	1	1	1
255	Gangtiejie	1	1	1	1	1	1	1	1	3	3	4	4	4	3	3	3	3	3	3
256	Fanghongjiniantagongjiaoshoumozhan	1	1	1	1	1	1	1	1	1	1	1	1	1	1	1	1	1	1	1
257	Zhongyangdajie	1	1	1	1	1	1	1	1	1	1	1	1	1	1	1	1	1	1	1
258	Hongxiajie	1	1	1	1	1	1	1	1	1	1	1	1	1	1	1	1	1	1	1
259	Jingweishiyidaojie	1	1	1	1	1	1	1	1	1	1	1	1	1	1	1	1	1	1	1
260	Aijianlu	1	1	1	1	1	1	1	3	3	3	3	1	1	1	1	1	2	2	1
261	Hezhoujie	1	1	1	1	1	1	1	1	1	1	1	3	4	3	3	3	4	3	3
262	Tielujie	1	1	1	1	1	1	1	1	1	1	1	1	1	1	1	1	3	3	3
263	Wajie	1	1	1	1	1	1	1	1	1	1	1	1	1	1	1	1	1	1	1
264	Xidazhijie	1	1	1	1	1	1	1	1	1	1	1	1	1	1	1	1	1	1	1
265	Hanguangjie(temporary station)	1	1	1	1	1	1	1	1	1	1	1	1	1	1	1	1	1	1	1
266	Wuruijie	1	1	1	1	1	1	1	1	1	1	1	1	1	3	2	1	1	1	1
267	Yikuangjie	1	1	1	1	1	1	1	1	1	1	1	1	1	1	1	1	1	1	1
268	Wangzhaoxincun	1	1	1	1	1	1	2	2	1	1	1	1	1	1	1	1	1	1	1
269	Tielueryuan	1	1	1	1	1	1	1	1	1	1	1	1	1	1	1	1	1	1	1
270	Wangzhaohuochezhan	1	1	1	1	1	1	1	1	1	1	1	1	1	1	1	1	1	1	1
271	Huaqiaomingyuanxiaoqu	1	1	1	1	1	2	2	3	3	4	4	4	3	2	2	2	2	2	1
272	Xiangbinluqiaokou	1	1	1	1	1	1	1	1	1	1	1	1	1	1	1	1	1	1	1
273	Shangyoujie(Zhongyangdajie)	1	1	1	1	1	1	1	1	1	1	1	1	1	1	1	1	1	1	1
274	Jianguogongyuan(Tongdajie)	1	1	1	1	1	1	3	3	3	3	4	4	2	2	3	3	3	3	3
275	Nanpingjie	1	1	1	1	1	1	2	1	1	1	1	1	1	1	1	1	1	1	1
276	Anshengjie(Xinyang Junction)	1	1	1	1	1	1	1	1	1	1	3	3	3	4	2	2	1	1	1
277	Steel Residential Area	1	1	1	1	1	1	1	1	1	1	3	4	3	3	3	3	3	3	3
278	Shiyiyuan	1	2	2	1	1	1	1	1	1	1	1	1	1	1	1	1	1	1	1
279	Zhaolingongyuan	1	1	1	1	1	1	1	1	1	1	1	1	1	1	1	1	1	1	1
280	Jingweijiudaojie	1	1	1	1	1	1	1	1	1	1	1	1	1	1	1	1	1	1	1
281	Anlongjie	1	2	1	1	1	1	1	1	1	1	1	1	1	1	1	1	1	1	1
282	Ansongjie	1	1	1	1	1	1	1	1	1	1	1	1	1	1	1	1	1	1	1
283	Yangmingjie	1	1	1	1	1	1	3	2	4	3	3	1	1	3	3	3	2	2	1
284	Aijianxinchengshoumozhan	1	1	1	1	1	2	1	2	2	2	1	1	1	1	1	1	1	1	1
285	Tongjiangjie	1	1	1	1	1	1	1	1	1	1	1	1	1	1	1	1	1	1	1
286	Shangyoujie	1	1	1	1	1	1	1	1	1	1	1	1	1	1	1	1	1	1	1
287	Hasanzhong	1	1	1	1	1	1	1	3	2	1	1	2	2	1	1	1	1	1	1
288	Guoxinzhengquangongsi	1	1	1	1	1	1	1	1	1	1	1	1	1	1	1	1	1	1	1
289	Youyigong	1	1	1	1	1	1	1	1	1	1	1	1	1	1	1	1	1	1	1
290	Gongchengjie	1	1	1	1	1	1	1	1	1	1	1	1	1	1	1	1	1	1	1
291	Chuangyezhongxin	1	1	1	1	1	1	1	1	1	1	1	1	2	1	1	1	1	1	1
292	Dadaojie	1	1	1	1	1	1	1	1	1	1	1	1	1	1	1	1	1	1	1
293	Yierwuzhong	1	1	1	3	3	3	3	3	1	1	1	1	1	1	1	1	1	1	1
294	Shengdianliyiyuan	1	1	1	1	1	1	1	2	2	2	1	1	3	2	3	2	2	2	2
295	Xiangjianglu	1	1	1	1	1	1	1	1	2	1	1	1	3	2	2	2	2	3	2
296	Heilongjiangshanglv(Hanshuilu)	1	1	1	1	1	1	1	1	1	1	1	1	1	1	1	1	1	1	1
297	Changjianglu	1	1	1	1	1	1	1	1	1	1	1	2	2	2	2	2	1	1	1
298	Huanghelu	1	1	1	1	1	1	3	3	3	1	1	1	1	1	1	1	1	1	1
299	Longyundasha(Lujiajie)	1	1	1	1	1	1	3	3	3	3	3	3	3	3	3	3	3	3	3

300	Zhuozhangouwuerqi	1	1	1	1	1	1	1	3	3	3	3	1	1	1	1	1	1	1	1
301	Shanghaijie	1	1	1	1	1	1	1	1	2	1	1	1	1	1	1	2	2	1	1
302	Jingyuqidaojie(Jingyudajie)	1	1	1	1	1	1	1	1	1	1	1	1	1	1	1	1	1	1	1
303	Taigushisidaojie	1	1	1	1	1	1	1	1	1	1	1	1	1	1	1	1	1	1	1
304	Yuhongxiaoxue	1	1	1	1	1	1	1	1	1	1	1	1	1	1	1	1	1	1	1
305	Benmaqipeicheng(Xianfenglu)	1	1	1	1	1	1	1	1	1	1	1	1	1	1	1	1	1	1	1
306	Shisanzhongxue	1	1	1	1	1	1	1	1	1	1	1	1	1	1	1	1	1	1	1
307	Hatiyuxueyuan	1	1	1	1	1	1	1	1	1	1	1	1	1	1	1	1	1	1	1
308	Haihelu(Xuanqingjie)	1	1	1	1	1	1	1	1	1	1	1	1	1	1	1	1	1	1	1
309	Yixuanguangtongzhaobiaojituan	1	1	1	1	1	1	1	1	1	1	1	1	1	1	1	1	1	1	1
310	Jiusanjituan	1	1	1	1	1	1	1	1	1	1	1	1	1	1	1	2	1	1	1
311	Minjiangxiaoqu	1	1	1	1	1	1	1	1	1	1	1	1	1	1	2	1	1	1	1
312	Taishanxiaoqu	1	1	1	1	1	1	1	1	1	1	1	1	1	1	1	1	1	1	1
313	Hongqixiaoqu	1	1	1	1	1	1	1	1	1	1	1	1	1	1	1	1	1	1	1
314	Huaihelu	1	1	1	1	1	1	1	1	1	1	1	1	1	1	1	1	1	1	1
315	Liaohexiaoqu	1	1	1	1	1	1	1	1	1	1	1	2	1	1	1	1	1	1	1
316	Zhongzhifangzhouyuan	1	1	1	1	1	1	1	1	1	1	1	1	1	1	1	1	1	1	1
317	Haihelu(Xuanqingjie Junction)	1	1	1	1	1	1	1	1	1	1	1	1	1	1	1	1	1	1	1
318	Shizijie	1	1	1	1	1	1	1	1	1	1	1	1	1	1	1	1	1	1	1
319	Shenggonganting	1	1	1	1	1	2	2	1	1	3	3	2	1	1	1	1	1	2	1
320	Wenjunhuayuan	1	1	1	1	1	1	2	2	2	3	3	3	4	3	4	4	3	3	3
321	Xianfenglu(Huashanbei Junction)	1	1	1	1	1	1	2	4	2	1	1	1	1	1	1	1	1	1	1
322	Liaohelu	1	1	1	1	1	1	1	1	1	1	1	1	1	1	1	1	1	1	1
323	Huaihelu(Hongqidajie Junction)	1	1	1	1	1	1	1	1	1	1	1	1	1	1	1	1	1	1	1
324	Huizhanzhongxin	1	1	1	1	1	1	1	1	1	1	1	1	1	1	1	1	1	1	1
325	Hanshuilu(Hongqidajie Junction)	1	1	1	1	1	1	1	1	1	1	1	2	1	1	1	1	1	1	1
326	Hongnanjie	1	1	1	1	1	1	1	1	1	1	1	1	1	1	1	1	1	1	1
327	Hongweilu	1	1	1	1	1	1	1	1	1	1	1	1	1	1	1	1	1	1	1
328	Shieryuan	1	1	1	1	1	1	1	1	1	1	1	1	1	1	1	1	1	1	1
329	Liaohelu(Hongqidajie Junction)	1	1	1	1	1	1	1	1	1	1	1	1	1	1	1	1	1	1	1
330	Huaihelu(Hongqidajiekou)	1	1	1	1	1	1	1	1	1	1	1	1	1	1	1	1	1	1	1
331	Xinyuanxiaoqu	1	1	1	1	1	1	1	1	1	1	1	1	2	1	1	1	1	1	1
332	Hagongdadierxiaoqu	1	1	1	1	1	1	1	1	1	1	1	2	1	2	2	2	1	1	1
333	Hongxianglu	1	1	1	1	1	1	1	1	1	1	1	1	1	1	1	1	1	1	1
334	Sandadonglilu	1	1	1	1	1	1	1	1	3	4	4	4	3	3	3	3	1	1	1
335	Zhaolingongyuanzhan	1	1	1	1	1	1	1	1	1	1	1	1	1	1	1	1	1	1	1
336	Shiyiyuanzhan	1	2	2	1	1	1	1	1	1	1	1	1	1	1	1	1	1	1	1
337	Xiashigushangkeyiyuanzhan	1	1	1	1	1	1	1	1	1	1	1	1	1	1	1	1	1	1	1
338	Hazhanbeiguangchanggongjiaoshoumozhan	1	1	1	1	1	1	1	1	1	1	1	1	1	1	1	1	1	1	1
339	Taihaihuayuanxiaoqu	1	1	1	1	1	3	3	1	1	1	1	1	1	1	1	1	1	1	1
340	Hongboshijiguangchang	1	1	1	1	1	1	1	1	1	1	1	2	2	1	1	1	1	1	1
341	Taishanlu	1	1	1	1	1	1	1	1	1	1	1	1	3	2	2	2	2	3	2
342	Huayuanjie	1	1	1	1	1	1	1	1	2	1	1	1	1	1	1	1	1	1	1
343	Yiererzhong	1	2	1	1	1	1	2	2	3	2	3	3	3	3	2	2	1	1	1
344	Liushunzongheshichang	1	1	1	1	1	1	1	1	1	1	1	1	1	1	1	1	1	1	1
345	Huashanlu(Ganshui Junction)	1	1	1	1	1	1	1	1	1	1	1	1	1	1	1	1	1	1	1
346	Chuangyezhongxin(temporary station)	1	1	1	1	1	1	1	1	1	1	1	1	2	1	1	1	1	1	1
347	Huaihelu(Hongqidajiejiekou)	1	1	1	1	1	1	1	1	1	1	1	1	1	1	1	1	1	1	1
348	Liaohelu(Hongqidajiejiekou)	1	1	1	1	1	1	1	1	1	1	1	1	1	1	1	1	1	1	1
349	Shiguoshuijichaju	1	1	1	1	1	1	1	1	1	1	1	1	1	1	1	1	1	1	1
350	Yimianjie(Senlinjie Junction)	1	1	1	1	1	1	1	1	1	1	1	1	1	1	1	1	1	1	1
351	Alaboguangchang(temporary station)	1	1	1	1	1	1	1	1	1	1	1	1	1	1	1	1	1	1	1
352	Nanxinershidaojie	1	1	1	1	1	1	1	1	1	1	1	1	1	1	1	1	1	1	1
353	Songshanlu	1	1	1	1	1	1	1	1	1	1	1	1	1	1	1	1	1	1	1
354	Guoluchanglinshizhan	1	1	2	2	1	2	2	2	1	2	3	3	3	3	3	2	2	2	1
355	Renliziyuanshichang(Anningjie)	1	1	1	1	1	1	1	1	1	2	2	2	2	2	2	1	1	1	2
356	Wangtongluyingyeting(Ershangdian)	1	1	1	1	1	2	1	1	1	1	1	1	1	1	2	2	2	2	1
357	Kunlunshangcheng(Hanshuilu)	1	1	1	1	1	1	1	1	1	1	1	1	1	1	1	1	1	1	1
358	Haguangdiantai	1	1	1	1	1	1	1	1	1	1	1	1	1	1	1	1	1	1	1
359	Hengdaojie(Gongbinlu)	1	1	1	1	1	1	2	2	2	2	2	2	2	1	1	2	1	1	1

360	Litchi Street	1	1	1	1	1	3	3	3	3	3	3	3	3	3	3	3	3	3	3
361	Xianfengxiaoqu(Huashan Junction)	1	1	1	1	1	1	2	4	2	1	1	1	1	1	1	1	1	1	1
362	Hashangdananxuequ	1	1	1	1	1	1	1	1	1	1	1	1	1	1	1	1	2	1	2
363	Fushunjiedidelijiekou(Fushunxiaoxue)	1	1	1	1	1	1	1	1	1	1	1	1	1	1	1	1	1	1	1
364	Beifangjuchang	1	1	1	1	1	1	1	1	1	1	1	1	1	1	1	1	1	1	1
365	Huashanluxianfenglulukou	1	1	1	1	1	1	2	4	2	1	1	1	1	1	1	1	1	1	1
366	Shengzhijianyuan	1	1	1	1	1	1	1	1	1	1	1	1	1	2	2	1	1	1	2
367	Xinjiangqiaoshichang	1	1	1	1	1	1	1	1	1	1	1	1	1	1	1	1	1	1	1
368	Taipingdajie	1	1	1	1	1	1	1	1	1	1	1	1	1	1	1	1	1	1	1
369	Taipingbeisandaojie	1	1	1	1	1	1	2	2	1	1	1	1	1	1	1	1	1	2	1
370	Taipingnansidaojie	1	1	1	1	1	1	1	1	1	2	1	1	1	1	1	1	1	2	1
371	Baqujie	1	1	1	1	1	1	1	1	1	1	1	1	1	1	1	1	1	1	1
372	Baqujienanjijiekou	1	1	1	1	1	1	1	1	1	1	1	1	1	1	1	1	1	1	1
373	Songpudaqiaonan	1	1	1	1	1	1	1	1	1	1	1	1	1	1	1	1	1	1	1
374	Nanzhixiaoqu	1	1	1	1	1	1	1	1	1	1	1	1	1	1	1	1	1	1	1
375	Wangtongdongzhiluyingyeting(Dongzhilu)	1	1	1	1	1	1	1	1	1	1	1	1	1	1	3	2	1	1	1
376	Yanpingjie	1	1	1	1	1	1	1	1	1	1	1	3	3	1	1	1	1	1	1
377	Dongdazhijie	1	1	1	1	1	1	1	1	1	1	2	2	1	1	1	2	3	1	2
378	Longyundasha	1	1	1	1	1	1	3	3	3	3	3	3	3	3	3	3	3	3	3
379	Hashidananxuequ	1	3	2	3	2	3	3	2	2	2	3	2	3	3	4	4	3	3	2
380	Zhongbeichuncheng	1	1	1	1	1	1	3	3	3	3	3	3	2	4	4	3	1	1	1
381	Zhongxin Department Store	1	1	1	1	1	2	3	3	3	3	3	3	3	2	2	2	1	1	3
382	Xinjiangqiaojie	1	1	1	1	1	1	1	1	1	1	1	1	1	1	1	1	1	1	1
383	Jiashujie	1	1	1	1	1	1	1	1	1	1	1	1	1	1	1	1	1	1	1
384	Hazhantielujie	1	1	1	1	1	1	1	1	1	1	1	1	1	1	1	1	1	1	1
385	Laoshichang	1	1	1	1	2	2	3	3	3	2	3	3	2	2	2	2	1	1	2
386	Linshizhan	1	1	1	1	1	1	1	1	1	1	1	4	4	3	3	3	3	3	3
387	Herunjie	1	1	1	1	1	1	1	1	1	1	1	3	2	1	2	2	1	1	1
388	Daowaiershidaojie	1	1	1	1	1	1	1	1	1	1	1	1	1	1	1	1	1	1	1
389	Daowaikeyunzhan	1	1	1	1	1	1	1	1	1	1	1	1	1	1	1	1	1	1	1
390	Tongdajie(Jianguobeierdaojie)	1	1	1	1	1	1	3	3	3	3	3	4	2	2	3	3	3	3	3
391	Minzhongjie	1	1	1	1	1	1	3	3	3	4	4	2	2	1	1	1	2	1	2
392	Xuanqingxiaoqu	1	1	1	1	1	1	1	2	3	1	1	1	1	2	1	1	1	1	1
393	Shengshizihui	1	1	1	1	1	3	3	3	2	2	2	1	1	1	1	1	1	1	1
394	Wenchangjiezhan	1	1	1	1	3	3	3	3	2	3	2	2	1	1	1	1	1	1	2
395	Hashida(Nanxiaoqu)	1	4	2	3	2	3	2	2	2	2	2	2	3	3	4	4	2	3	2
396	Taiping Second Shop	1	1	1	1	1	1	1	1	1	1	1	1	1	1	1	1	1	2	1
397	Beijingjie(Manzhoulijiekou)	1	1	1	1	1	1	1	1	1	1	1	1	1	1	1	1	1	1	1
398	Hongqidajiegongbinlukou	1	1	1	1	1	2	3	3	3	3	3	3	3	2	2	2	1	1	1
399	Taipingsandaojie	1	1	1	1	1	1	2	2	1	1	1	1	1	1	1	1	1	2	1
400	Taipingsidaojie	1	1	1	1	1	1	1	1	1	2	1	1	1	1	1	1	1	2	1
401	Tongjiangshidigongjiaochuanchengshuniuzhan	1	1	1	1	1	1	1	1	1	1	1	1	1	1	1	1	1	1	1
402	Yimianjie	1	1	1	1	1	1	1	1	1	1	1	1	1	1	1	1	1	1	1
403	Taigushierdaojie	1	1	1	1	1	1	1	1	1	1	1	1	1	1	1	1	3	1	1
404	Taigushiliudaojie	1	1	1	1	1	1	1	1	1	1	1	2	2	1	1	1	1	1	1
405	Xianfenglu(Huashan Junction)	1	1	1	1	1	1	1	1	1	1	1	1	1	1	1	1	1	1	1
406	Harbintiyuxueyuan	1	1	1	1	1	1	1	1	1	1	1	1	1	1	1	1	1	1	1
407	Lvseshipinjiaoyizhongxin	1	1	1	1	1	1	1	1	1	1	1	1	1	1	1	1	1	1	1
408	Hanguangjie	1	1	1	1	1	1	1	1	1	1	1	1	1	1	1	1	1	1	1
409	Qizhengjie	1	1	1	1	1	1	1	1	1	1	1	1	1	1	1	1	1	1	1
410	Boyuexingchenggongjiaoshoumozhan	1	1	1	1	1	1	1	1	1	1	1	1	1	1	1	1	1	1	1
411	Zhaolinjie	1	1	1	1	1	1	1	1	1	1	1	1	1	1	1	1	1	1	1
412	Hongqixiaoqu(temporary station)	1	1	1	1	1	1	1	1	1	1	1	1	1	1	1	1	1	1	1
413	Sandadonglifulu	1	1	1	1	1	1	3	3	3	2	3	3	3	3	3	3	3	3	1
414	Hanshuilu(Hongqidajiekou)	1	1	1	1	1	1	1	1	1	1	1	1	1	1	1	1	1	1	1
415	Kunlunxiaoqu	1	1	1	1	1	1	1	1	1	1	1	1	1	1	1	1	1	1	1
416	Weixinglu	1	1	1	1	1	1	1	1	1	1	1	1	1	1	1	1	1	1	1
417	Wenhuadasha(temporary station)	1	1	1	1	1	1	1	1	1	1	1	1	1	1	1	1	1	1	1
418	Tiandijie	1	1	1	1	1	1	1	1	1	1	1	1	1	1	1	1	1	1	1
419	Yimianjie(temporary station)	1	1	1	1	1	1	1	1	1	1	1	1	1	1	1	1	1	1	1

420	Youyigong(temporary station)	1	1	1	1	1	1	1	1	1	1	1	1	1	1	1	1	1	1	1
421	Xixiangfang(Gongbinlu)	1	1	1	1	1	1	1	1	1	3	3	3	3	2	2	2	1	1	1
422	Huizhanzhongxintiyuchang	1	1	1	1	1	1	1	1	1	1	1	2	1	1	1	1	1	1	1
423	Hazhan(Haichengqiao)	1	1	1	1	1	1	1	1	1	1	1	1	1	1	1	1	1	1	1
424	Nanxunbadaojie	1	1	1	1	1	1	1	1	1	1	1	1	1	1	1	1	1	1	1
425	Jingyujie	1	1	1	1	1	1	1	1	1	1	1	1	1	1	1	1	1	1	1
426	Anfengshangchang	1	1	1	1	1	1	1	1	1	1	1	1	1	1	1	1	1	1	1
427	Jingweijie(Zhongyangdajie)	1	1	1	1	1	1	1	1	1	1	1	2	1	1	1	1	1	1	1
428	Heilongjianggongchengdaxue	1	1	1	1	1	1	1	1	1	1	1	1	1	1	1	1	1	1	1
429	Gexinjiezhan	1	1	1	1	1	1	1	1	1	1	1	1	1	1	1	1	1	1	1
430	Shengdanganshi	1	1	1	1	1	1	1	1	1	1	1	1	1	1	1	1	1	1	1
431	Shizijie(Dachengjiekou)	1	1	1	1	1	1	1	1	1	1	1	1	1	1	1	1	1	1	1
432	Maduanjie	1	1	1	1	1	1	1	1	1	1	1	1	1	1	1	1	1	1	1
433	Hatiyuxueyuandongmen	1	1	1	1	1	1	1	1	1	1	1	1	1	1	1	1	1	1	1
434	Jiangongxinqu	1	1	1	1	1	1	1	1	1	1	1	1	1	1	1	1	1	1	1
435	Hazhan(Haichengqiao)(temporary station)	1	1	1	1	1	1	1	1	1	1	1	1	1	1	1	1	1	1	1
436	Nanxinshisidaojie	1	1	1	1	1	1	1	1	1	1	1	1	1	1	2	2	1	1	1
437	Huangjiahuayuan	1	1	1	1	1	1	2	3	2	2	1	4	3	2	2	2	1	1	1
438	Gongludaqiao(Heluojie)	1	1	1	1	1	1	1	1	1	1	1	1	3	3	3	3	3	4	3
439	Nanjijie(Chengdejie)	1	1	1	1	1	1	1	1	1	1	1	1	1	1	1	1	1	1	1
440	Hongzhuanjie	1	1	1	1	1	1	1	1	1	1	1	1	1	1	1	1	1	1	1
441	Gongchengjie(temporary station)	1	1	1	1	1	1	1	1	1	1	1	1	1	1	1	1	1	1	1
442	Xiangjianglu(Hongqidajie Junction)	1	1	1	1	1	1	1	1	2	1	1	1	3	2	2	2	2	3	2
443	Xiangshunjie	1	1	1	1	1	1	1	1	1	1	1	1	1	1	1	1	1	1	1
444	Xiaofangyiyuan	1	1	1	1	1	1	1	1	1	1	1	1	1	1	1	1	1	1	1
445	Hasanzhong(temporary station)	1	1	1	1	1	1	1	3	2	2	2	2	2	1	1	1	1	1	1
446	Heilongjiangkejixueyuan(Songshanxiaoqu)	1	1	1	1	1	1	1	1	1	1	1	2	3	2	2	2	1	1	1
447	Benmaqipeicheng	1	1	1	1	1	1	1	1	1	1	1	1	1	1	1	1	1	1	1
448	Dongbeiyiyuan	1	1	1	1	1	1	1	1	1	1	1	1	1	1	1	1	1	1	1
449	Fanrongjie	1	1	1	1	1	1	1	1	1	1	1	1	1	1	1	1	1	1	1
450	Taigushisidaojie(temporary station)	1	1	1	1	1	1	1	1	1	1	1	1	1	1	1	1	1	1	1
451	Hongqidajie(Beihuanlu Junction)	1	1	1	1	1	1	1	1	1	1	1	1	1	1	1	1	1	1	1
452	Jingyudajie	1	1	1	1	1	1	1	1	1	1	1	1	1	1	1	1	1	1	1
453	Shengyiyuannangnagfenyuan	1	1	1	1	1	1	1	1	1	1	1	2	1	1	1	1	1	1	1
454	Quxianjie	1	1	1	3	3	3	3	3	3	2	2	2	3	4	4	4	3	3	3
455	Tieludiyixiaoxue	1	1	1	1	1	1	1	1	1	1	1	1	1	1	1	1	1	1	1
456	Wenhuadasha	1	1	1	1	1	1	1	1	1	1	1	1	1	1	1	1	1	1	1
457	Kunlunshangcheng	1	1	1	1	1	1	1	1	1	1	1	1	1	1	1	1	1	1	1
458	Hequjie	1	1	1	1	1	1	1	1	1	1	1	1	3	3	3	3	3	4	3
459	Changjianglunanzhilukou	1	1	1	1	1	1	1	1	1	1	1	1	1	1	1	1	1	1	1
460	Nanzhiluhanshuilukou	1	1	1	1	1	1	1	1	1	1	1	1	1	1	1	1	1	1	1
461	Liushunjie(Situjie Junction)	1	1	1	1	1	1	1	1	1	1	1	1	1	1	1	1	1	1	1
462	Tongdajie(Jianguogongyuan)	1	1	1	1	1	1	3	3	3	3	3	4	2	2	3	3	3	3	3
463	Anshunjie	1	1	1	1	1	1	1	1	1	1	1	1	1	1	1	1	1	1	1
464	Minjianglu(Nanzhilu Junction)	1	1	1	1	1	1	1	1	1	1	1	1	1	1	1	1	1	1	1
465	Xidazhijie(temporary station)	1	1	1	1	1	1	1	1	3	1	1	1	1	3	2	2	2	2	3
466	Shengzhengxie	1	1	1	1	1	2	4	2	2	3	3	4	3	3	3	3	2	2	1
467	Tianmuxiaoqu	1	1	1	1	1	1	3	1	1	1	1	1	1	1	1	1	1	1	1
468	Renlijie	1	1	1	1	1	1	1	1	1	1	1	1	1	1	1	1	1	1	1
469	'angtongdongzhiluyingyeting(temporary statio	1	1	1	1	1	2	1	1	1	1	1	1	1	1	2	2	2	2	1
470	Yaocaigongsi	1	1	1	1	1	1	1	1	1	1	1	1	1	1	1	1	1	1	1
471	Huaihelu(temporary station)	1	1	1	1	1	1	1	1	1	1	1	1	1	1	1	1	1	1	1
472	Qianjinlu(Hesongjiekou)	1	1	1	1	1	1	1	1	3	1	1	4	3	3	3	3	3	3	3
473	Xiashigushangyiyuan	1	1	1	1	1	1	1	1	1	1	1	1	1	1	1	1	1	1	1
474	Xiangdianjie	1	1	1	1	1	1	1	1	1	1	1	1	1	1	2	2	1	1	1
475	Huashanlu(Changjianglu Junction)	1	1	1	1	1	1	1	1	1	1	1	2	2	2	2	2	1	1	1
476	Shengbolanzhongxinzhan	1	1	1	1	1	1	1	1	1	2	3	3	2	1	1	1	1	1	1
477	Hongqidajie(Beihuanlukou)	1	1	1	1	1	1	1	1	1	1	1	1	1	1	1	1	1	1	1
478	Lujiajie	1	1	1	1	2	1	1	1	1	1	1	2	2	1	1	1	1	1	1
479	Renhejie	1	1	1	1	1	2	1	2	2	1	1	1	1	1	1	1	1	1	1

480	Huayuanshangdian	1	1	1	1	1	1	1	1	1	1	1	1	1	1	1	1	1	1	1
481	Liaoyangjie	1	1	1	1	1	1	1	1	1	2	2	3	2	1	1	1	1	1	1
482	Ankangjie	1	1	1	1	1	1	1	1	1	1	1	1	1	1	1	1	1	1	1
483	Jingweijie(Gaoyijie)	1	1	1	1	1	1	1	1	1	1	1	1	1	1	1	1	1	1	1
484	Jianguojie(Kanganlulukou)	1	1	1	1	1	3	2	3	3	2	3	3	4	4	3	3	3	3	3
485	Yaoliujiayuan(temporary station)	1	1	1	1	1	1	1	1	1	1	1	1	1	1	1	1	1	1	1
486	Shierzhongxue	1	2	2	1	1	1	1	1	1	1	1	1	1	1	1	1	1	1	1
487	Lujiajie(Shizijie Junction)	1	1	1	1	1	1	1	1	1	1	1	1	1	1	1	1	1	1	1
488	Renhejie(Jianxinjiekou)	1	1	1	1	1	1	1	1	1	1	1	1	1	1	1	1	1	1	1
489	Harbin Print Center	1	1	1	1	1	1	2	2	3	2	3	3	3	3	3	3	3	3	3
490	Miaopujie	1	1	1	1	1	1	1	1	1	1	1	1	1	1	1	1	1	1	1
491	Shierzhong	1	2	2	1	1	1	1	1	1	1	1	1	1	1	1	1	1	1	1
492	Bowuguanzhan(temporary station)	1	1	1	1	1	1	1	1	1	1	1	1	1	1	1	1	1	1	1
493	Hongqidajie	1	1	1	1	1	1	1	1	1	1	1	1	1	1	1	1	1	1	1
494	Tielujie(Shangfangjie)	1	1	1	1	1	1	1	1	1	1	1	1	1	1	1	1	1	1	1
495	Tielujie(temporary station)	1	1	1	1	1	1	1	1	1	1	1	1	1	1	1	1	3	1	3
496	Tielujieqiaobeijiekou	1	1	1	1	1	1	1	1	1	1	1	1	1	1	1	1	1	1	1
497	Tielujieqingmingerdaokou	1	1	1	1	1	1	1	1	1	1	1	1	1	1	1	1	1	1	1
498	Binjianghuozhan	1	1	1	1	1	1	1	1	1	1	1	1	1	1	1	1	3	1	1
499	Huochezhan	1	1	1	1	1	1	1	1	1	1	1	1	1	1	1	1	1	1	1
